# Supplementary material for: Astrocyte-Secreted Glypican 4 Regulates Release of Neuronal Pentraxin 1 from Axons to Induce Functional Synapse Formation
Source: Neuron. 2017 Oct 11;96(2):428–445.e13. doi: 10.1016/j.neuron.2017.09.053 (PMC5663462; doi:10.1016/j.neuron.2017.09.053)
Supplement: Document S1. Figures S1–S8 and Tables S2 and S3 [file mmc1.pdf]

Neuron, Volume *96*

Supplemental Information

Astrocyte-Secreted Glypican 4 Regulates Release of Neuronal Pentraxin 1 from Axons to Induce Functional Synapse Formation

Isabella Farhy-Tselnicker, Adriana C.M. van Casteren, Aletheia Lee, Veronica T. Chang, A. Radu Aricescu, and Nicola J. Allen

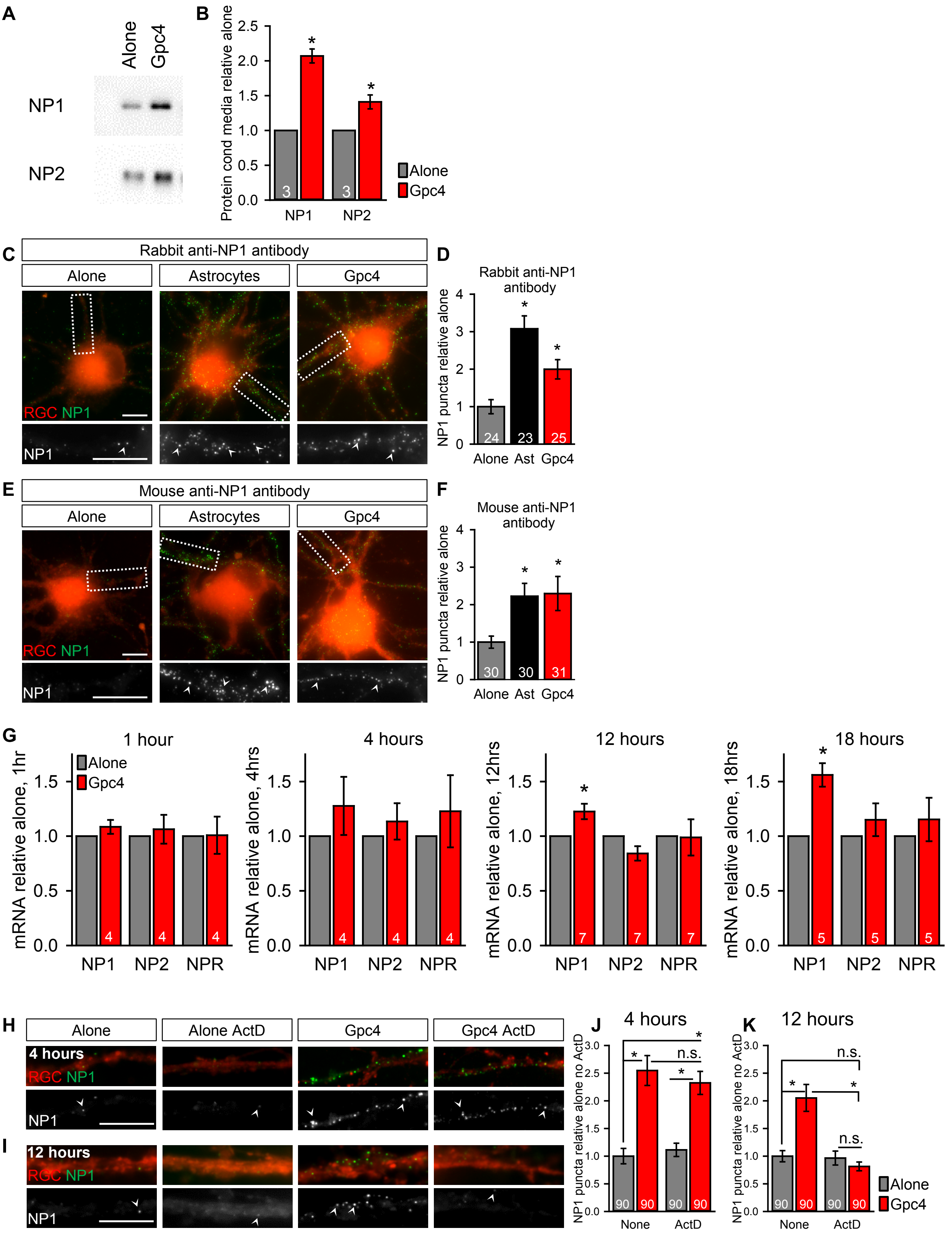
A
Alone
Gpc4
NP1
NP2
B
Protein cond media relative alone
Alone
Gpc4
C
Rabbit anti-NP1 antibody
Alone
Astrocytes
Gpc4
RGC NP1
NP1
D
Rabbit anti-NP1 antibody
NP1 puncta relative alone
Alone Ast Gpc4
E
Mouse anti-NP1 antibody
Alone
Astrocytes
Gpc4
RGC NP1
NP1
F
Mouse anti-NP1 antibody
NP1 puncta relative alone
Alone Ast Gpc4
G
1 hour
4 hours
12 hours
18 hours
mRNA relative alone, 1hr
mRNA relative alone, 4hrs
mRNA relative alone, 12hrs
mRNA relative alone, 18hrs
Alone
Gpc4
NP1 NP2 NPR
H
Alone
Alone ActD
Gpc4
Gpc4 ActD
4 hours
RGC NP1
NP1
I
12 hours
RGC NP1
NP1
J
4 hours
NP1 puncta relative alone no ActD
n.s.
None ActD
K
12 hours
NP1 puncta relative alone no ActD
n.s.
None ActD
Alone
Gpc4

Fig S1

Figure S1 (Relates to Figure 1). Gpc4 upregulates release of the AMPAR clustering factor NP1 from RGC neurons. A, B. RGCs treated with soluble Gpc4 for 4 days increase secretion of neuronal pentraxins into the conditioning media (CM). **A.** Western blot of RGC CM from neurons grown +/- Gpc4, blotted against NP1 or NP2. Approximate band size is 50 KDa. **B.** Quantification of Western blots in A, normalized to RGC alone. N=3 experiments. **C-F.** RGCs treated with an astrocyte feeder layer or soluble Gpc4 for 6 days show increased surface accumulation of NP1, demonstrated with two different anti-NP1 antibodies. The data in the main figure is with mouse anti-NP1 from BD Transduction Labs. **C,D** Rabbit anti-NP1. **E,F** Mouse anti-NP1. **C,E.** Example images: green surface NP1, red labels whole cell. Inset shows enlarged dendritic region from box, surface NP1 white. **D,F.** Quantification of C,E, number of surface NP1 puncta per RGC normalized to alone condition, from one representative experiment. For **C-F** Scale bar = 10μm. Arrowheads mark example puncta of NP1. Graphs show mean±s.e.m., number of cells per group inside the bar. **G.** qRT-PCR analysis of mRNA levels for NP1, NP2 and NPR in RGCs treated with soluble Gpc4 for varying times (1 hour, 4 hours, 12 hours, 18 hours). Graphs show mean±s.e.m., number of experiments inside the bar. **H-K.** RGCs treated with the transcription inhibitor Actinomycin D (ActD) show increased surface accumulation of NP1 in response to soluble Gpc4 after 4 hours (**H,J**) but not after 12 hours treatment (**I,K**). **H,I** Example dendrite images. Top: green surface NP1, red labels whole process. Bottom: single channel NP1, white. Scale bar = 10μm. Arrowheads mark example puncta of NP1. **J,K** Quantification of H and I respectively, number of surface NP1 puncta per RGC normalized to alone condition. N=3 experiments for each treatment time. Graphs show mean±s.e.m. number of cells per group inside the bar. * $p \leq 0.05$, by t-test when comparing 2 groups or one-way ANOVA when comparing 3 or more groups.

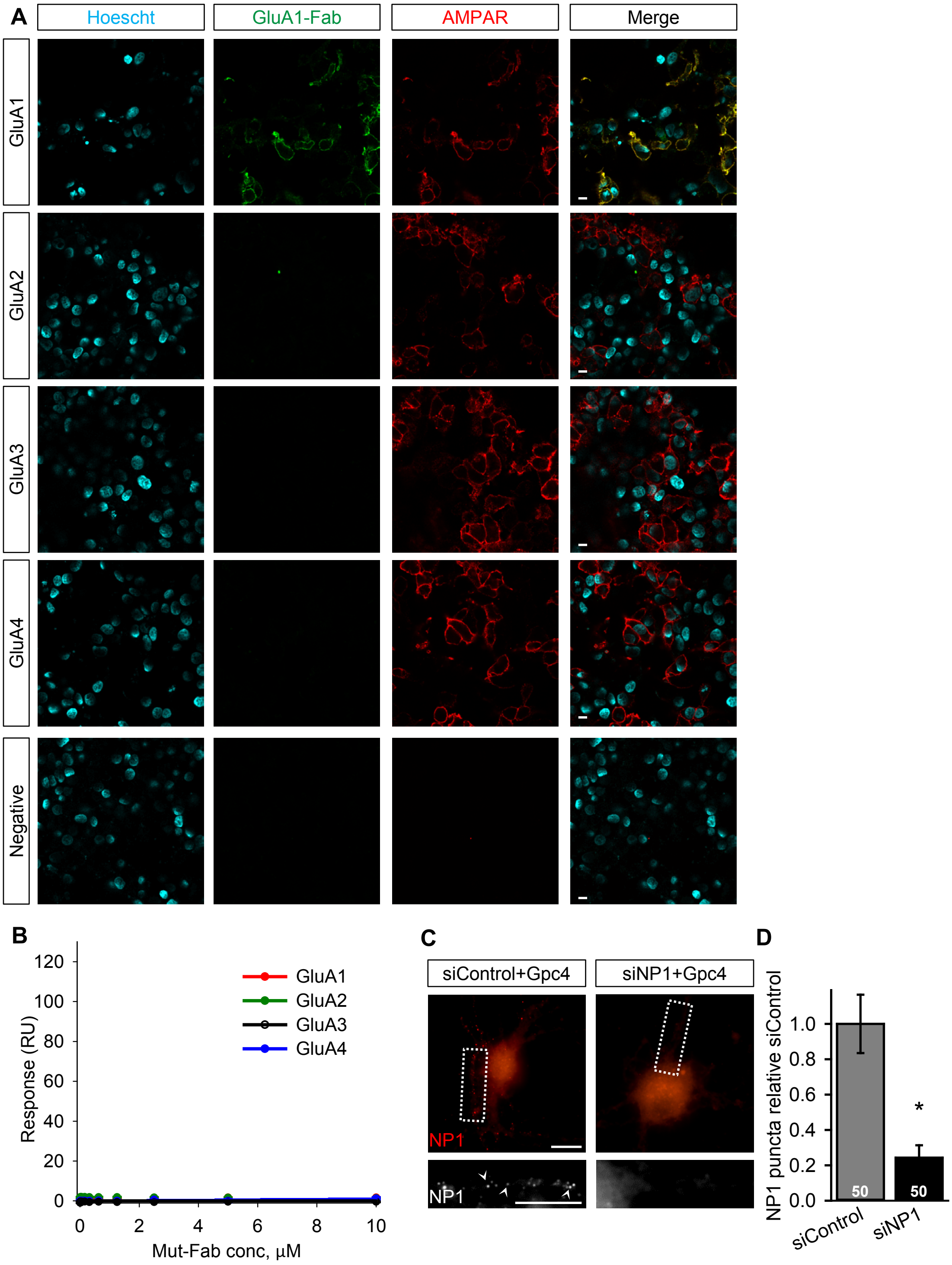
A
Hoescht
GluA1-Fab
AMPAR
Merge
GluA1
GluA2
GluA3
GluA4
Negative
B
Response (RU)
120
100
80
60
40
20
0
GluA1
GluA2
GluA3
GluA4
0
2
4
6
8
10
Mut-Fab conc, µM
C
siControl+Gpc4
siNP1+Gpc4
NP1
NP1
D
NP1 puncta relative siControl
1.0
0.8
0.6
0.4
0.2
0.0
50
50
*
siControl
siNP1

Fig S2

Figure S2 (Relates to Figure 2). **NP1-GluA1 interaction is necessary for Gpc4 to induce structural synapse formation. A,B.** The GluA1-Fab is specific for the N-terminal domain of GluA1 over other AMPAR subunits. **A.** Example images of HEK293T cells transfected with HA-tagged (GluA1, GluA3, GluA4) or FLAG-tagged (GluA2) AMPAR and co-immunostained for surface expression of the receptor and GluA1-Fab. Cell nuclei labeled with Hoescht (cyan) are shown on the left panels, followed by GluA1-Fab (green), AMPAR (red) and the merged image shown on the right. Note GluA1-Fab staining only visible in cells expressing the GluA1 subunit (top row). Negative control shows no signal (bottom row of images). Scale bar = 10μm. **B.** Surface plasmon resonance analysis of Mut-Fab binding. The Mut-Fab does not bind GluA1,2,3 or 4 AMPARs. **C-D.** siRNA against NP1 causes a significant decrease in surface NP1 accumulation in RGCs. **C**. Example images red: surface NP1. Scale bar = 10μm. Inset shows enlarged dendritic region from box, surface NP1 white. Scale bar = 10μm. Arrowheads mark example puncta of NP1. **D**. Quantification of C, number of surface NP1 puncta per RGC normalized to siControl+Gpc4 condition. N=2 experiments. Graphs show mean±s.e.m., number of cells per group inside the bar. * $p \leq 0.05$, by t-test.

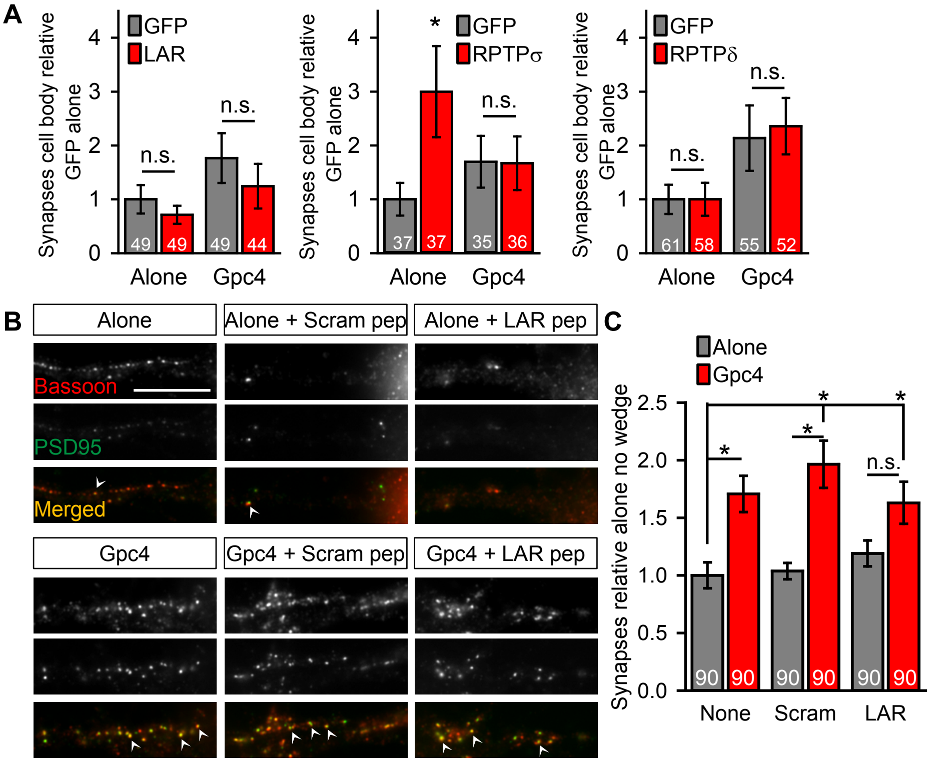
A
Synapses cell body relative GFP alone
GFP
LAR
RPTPσ
RPTPδ
n.s.
*
Alone
Gpc4
B
Alone
Alone + Scram pep
Alone + LAR pep
Bassoon
PSD95
Merged
Gpc4
Gpc4 + Scram pep
Gpc4 + LAR pep
C
Synapses relative alone no wedge
None
Scram
LAR

Fig S3

Figure S3 (Relates to Figure 3). Glypican 4 acts through presynaptic RPTPs to increase synapse formation. A. Expression of RPTP δ, σ or LAR in RGC dendrites does not alter the number of synapses formed in response to Gpc4. N = 3 experiments for each RPTP. **B-C.** Blocking LAR function with cell permeant wedge peptides does not alter Gpc4-mediated synapse formation. **B.** Example dendrite images, Bassoon red, PSD95 green, scale bar = 10μm. Arrowheads mark example synapses. **C.** Quantification of B, synapse number per cell normalized to alone no peptide condition, N = 3 experiments. Graphs show mean±s.e.m., number of cells per group inside the bar. * $p \leq 0.05$, by one-way ANOVA.

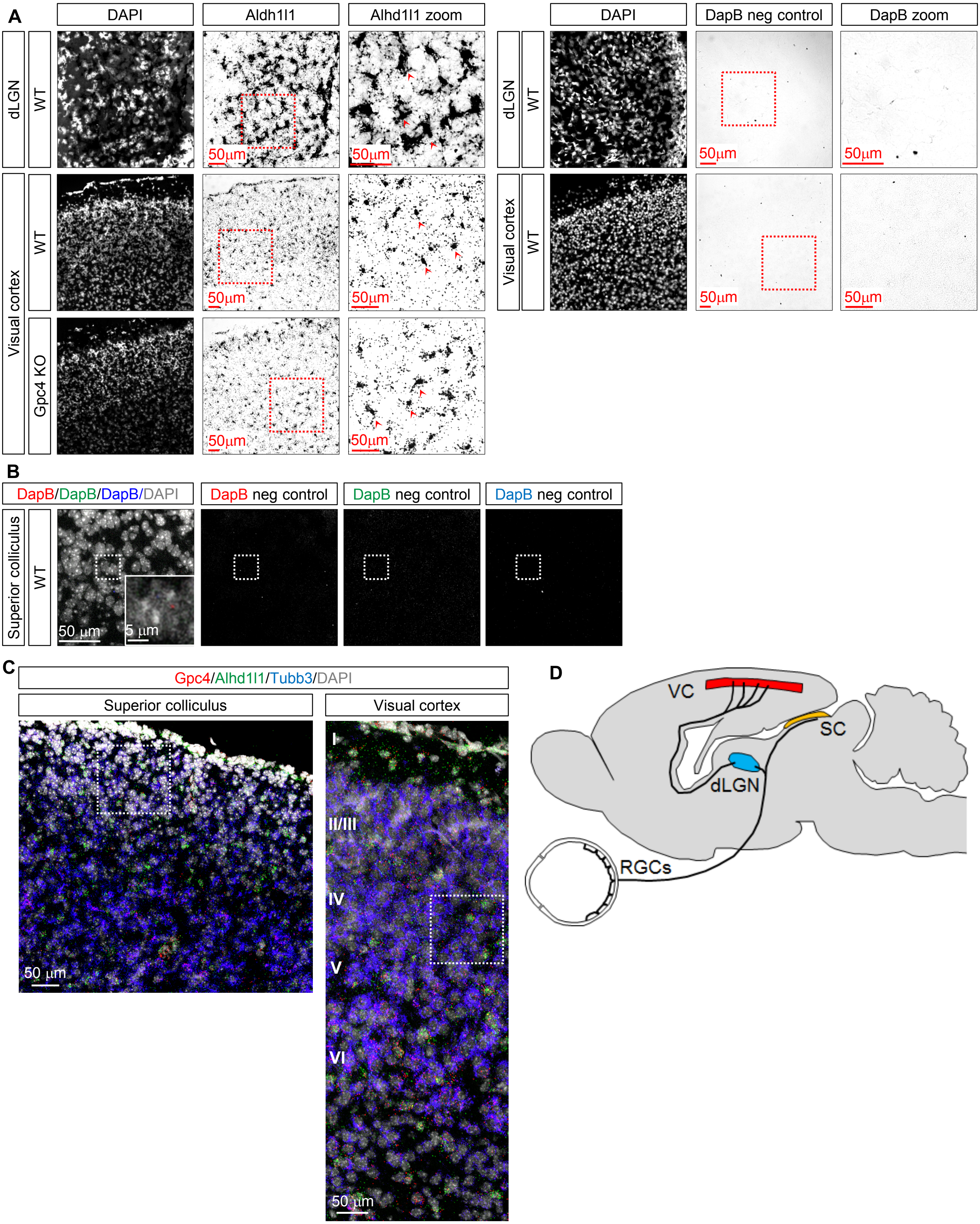
A
DAPI
Aldh1l1
Alhd1l1 zoom
DapB neg control
DapB zoom
dLGN
WT
Visual cortex
Gpc4 KO
50µm
B
DapB/DapB/DapB/DAPI
DapB neg control
Superior colliculus
50 µm
5 µm
C
Gpc4/Alhd1l1/Tubb3/DAPI
Superior colliculus
Visual cortex
I
II/III
IV
V
VI
50 µm
D
VC
SC
dLGN
RGCs

Fig S4

Figure S4 (Relates to Figure 5). Expression of Gpc4 in the developing visual system. A. Left side, ISH in the developing visual system at P6 shows expression of mRNA for the astrocyte marker Aldh1l1, demonstrating astrocytes are present at the time when synapse formation is initiated – in the dLGN (top), and visual cortex of WT (middle) and Gpc4 KO (bottom). Right side, negative control probe, showing absence of signal in the dLGN (top) and visual cortex (bottom). In each set, left panels show DAPI to mark cell nuclei, middle panels are low power images of the mRNA probe, scale bar = 50µm, right panels are high power images of the region outlined by the red box in the low power image, red arrowheads mark positive cells, scale bar = 50µm. Note the different image size for VC and dLGN. **B.** Triple ISH using the negative control probes, showing absence of signal in each channel. DAPI (grey) marks cell nuclei. Inset shows zoom in of boxed region. Scale bar = 50µm, zoomed in box scale bar = 5µm. **C.** Overview of triple ISH images of superior colliculus (left) and visual cortex (right). DAPI (grey, nuclei), Gpc4 (red), Aldh1l1 (green, astrocytes), Tubb3 (blue, neurons). Box shows the area used for quantification described in Fig 5C-F. **D.** Diagram of the mouse visual system showing RGC axons project from the retina to the superior colliculus (SC) and dorsal lateral geniculate nucleus of the thalamus (dLGN). Visual cortex (VC) is innervated by axons projecting from dLGN neurons. Adapted from (Berson, 2003).

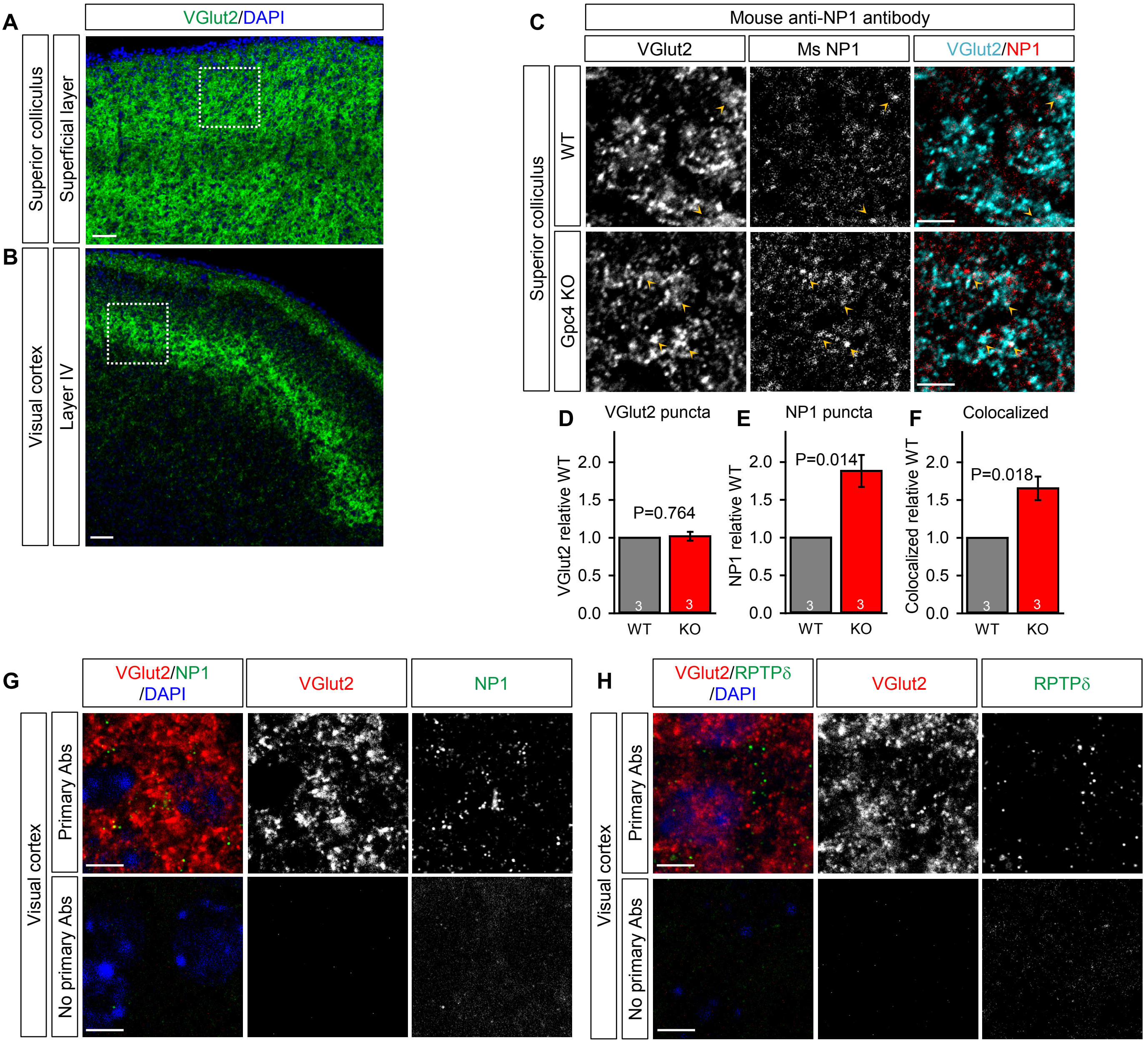
A
VGlut2/DAPI
Superior colliculus
Superficial layer
B
Visual cortex
Layer IV
C
Mouse anti-NP1 antibody
VGlut2
Ms NP1
VGlut2/NP1
Superior colliculus
WT
Gpc4 KO
D
VGlut2 puncta
VGlut2 relative WT
P=0.764
E
NP1 puncta
NP1 relative WT
P=0.014
F
Colocalized
Colocalized relative WT
P=0.018
2.0
1.5
1.0
0.5
0.0
3
WT
KO
G
VGlut2/NP1 /DAPI
VGlut2
NP1
Visual cortex
Primary Abs
No primary Abs
H
VGlut2/RPTPδ /DAPI
VGlut2
RPTPδ

Fig S5

Figure S5 (Relates to Figure 6). Gpc4 KO mice have increased overlap of NP1 with presynaptic terminals in the developing visual system *in vivo*. A-B. Low power confocal images of VGlut2 staining in superficial layers of the superior colliculus (A, SC) and layer 4 of the visual cortex (B, VC). White boxes mark example areas from which high power images in Fig 6-8 were taken. **C-F.** Increased colocalization between NP1 and the presynaptic marker VGlut2 in Gpc4 KO mice in the SC at P6, suggests reduced release of NP1 in the absence of Gpc4. Experiment carried out with mouse anti-NP1 from BD Transduction Labs. NP1 staining in the main figure is conducted with rabbit anti-NP1 from Thermo Fisher. The same result is seen with both anti-NP1 antibodies. **C.** Example images from the SC of WT (top) and Gpc4 KO (bottom), VGlut2 in cyan and NP1 in red. Representative colocalized puncta marked with arrowheads. Scale bar = 5 μm. **D,E,F.** Quantification of VGlut2, NP1 and colocalized puncta respectively, normalized to WT. Graphs show mean±s.e.m., number of mice inside the bar. Statistical analysis by t-test, P value shown on each graph. **G-H.** Representative images taken from the visual cortex with (top) and without (bottom) primary antibodies. VGlut2 red, NP1 green (G), RPTPδ green (H); DAPI in blue. Imaging the secondary antibody only condition with the same settings used for the primary antibody condition revealed no signal. Scale bar = 5 μm.

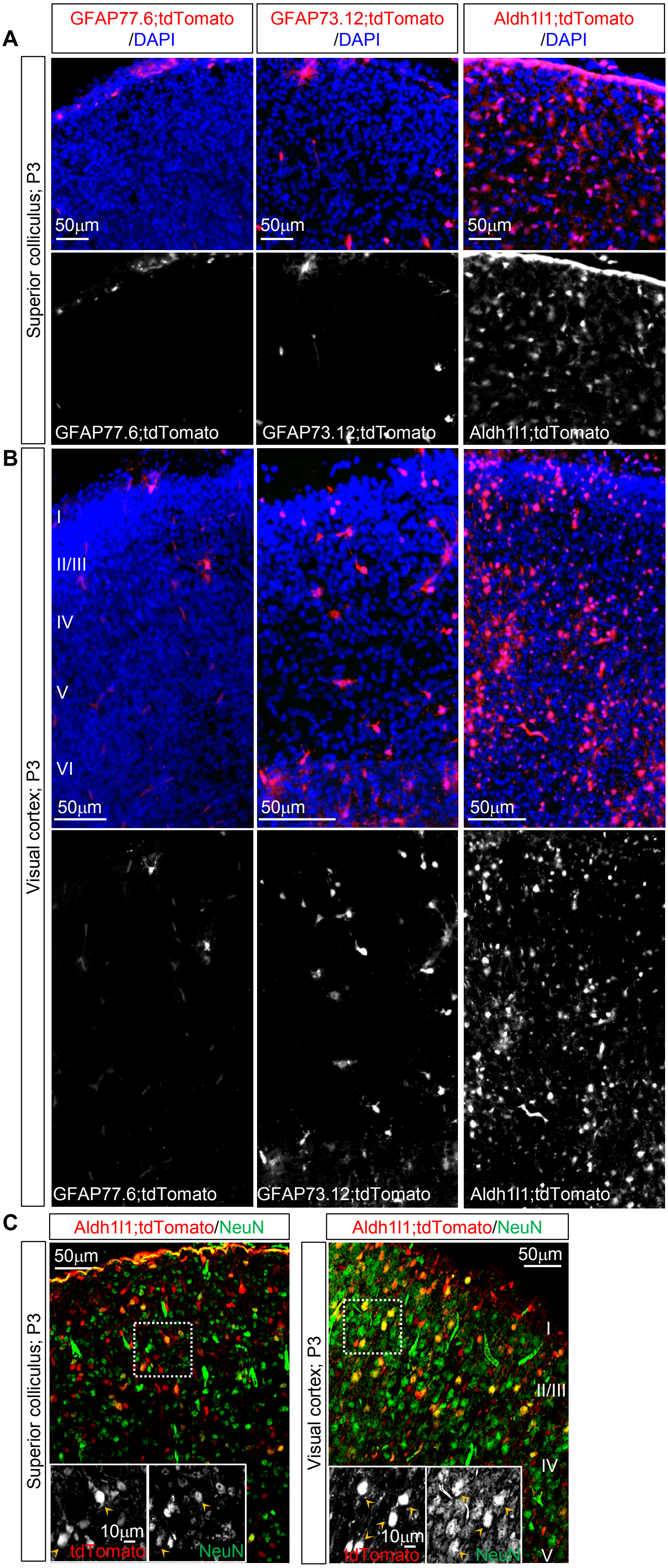
A
GFAP77.6;tdTomato/DAPI
GFAP73.12;tdTomato/DAPI
Aldh1l1;tdTomato/DAPI
Superior colliculus; P3
50µm
GFAP77.6;tdTomato
GFAP73.12;tdTomato
Aldh1l1;tdTomato
B
Visual cortex; P3
I
II/III
IV
V
VI
C
Aldh1l1;tdTomato/NeuN
10µm
tdTomato
NeuN

Fig. S6

Figure S6 (Relates to Figure 6). Characterization of astrocyte specific cre mouse lines. A-B. 3 different astrocyte specific cre lines GFAPcre77.6 (left); GFAPcre73.12 (middle) and Aldh1l1cre (right) were crossed to a tdTomato reporter line to visualize regions of cre expression in the SC (**A**) and VC (**B**) at P3. No substantial tdTomato expression was detected in GFAPcre77.6 in either brain region (left). No tdTomato expression was detected in GFAPcre73.12 SC (middle), whereas several tdTomato positive cells were detected in the VC. Robust tdTomato expression was detected in Aldh1l1cre line in both brain regions (right). DAPI (blue) marks cell nuclei, red marks tdTomato expressing cre positive cells. Below each merged image is a single channel tdTomato image. **C.** Immunostaining for the neuronal marker NeuN in Aldh1l1cre;tdTomato mouse brain, in the SC (left) and VC (right). Insets show zoom in box for single channel tdTomato (left) and NeuN (right). Scale bar = 50 μm, zoomed in box scale bar = 10 μm. Limited overlap of tdTomato with NeuN was observed in SC, while greater overlap was detected in VC. Arrowheads mark example overlapped cells.

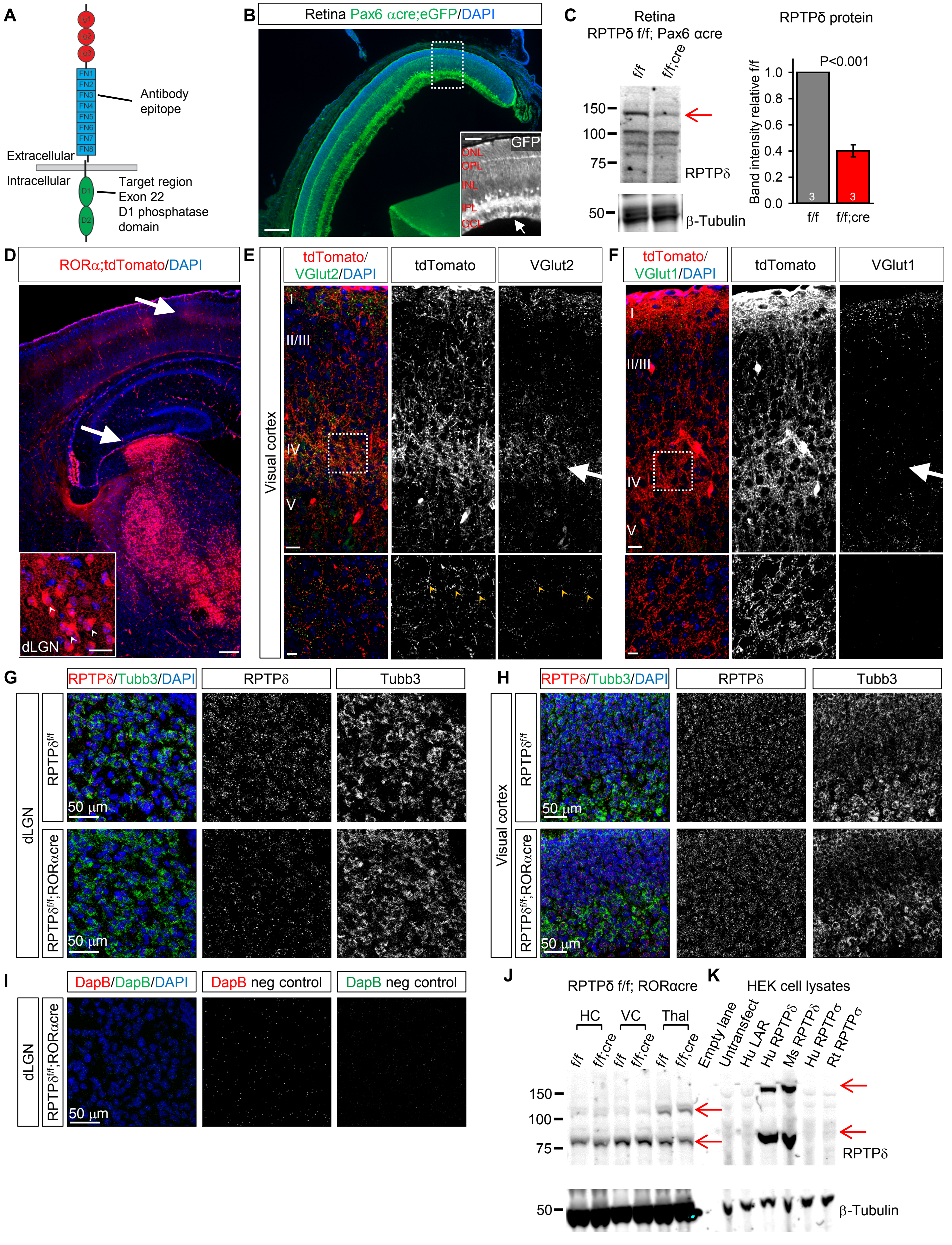
A
Ig1
Ig2
Ig3
FN1
FN2
FN3
FN4
FN5
FN6
FN7
FN8
Antibody epitope
Extracellular
Intracellular
D1
D2
Target region
Exon 22
D1 phosphatase domain
B
Retina Pax6 αcre;eGFP/DAPI
GFP
ONL
OPL
INL
IPL
GCL
C
Retina
RPTPδ f/f; Pax6 αcre
f/f
f/f;cre
150
100
75
50
RPTPδ
β-Tubulin
RPTPδ protein
P<0.001
Band intensity relative f/f
1.0
0.8
0.6
0.4
0.2
0.0
3
3
f/f
f/f;cre
D
RORα;tdTomato/DAPI
dLGN
E
Visual cortex
tdTomato/VGlut2/DAPI
tdTomato
VGlut2
I
II/III
IV
V
F
tdTomato/VGlut1/DAPI
tdTomato
VGlut1
I
II/III
IV
V
G
dLGN
RPTPδ/Tubb3/DAPI
RPTPδ
Tubb3
RPTPδf/f
RPTPδf/f;RORαcre
50 μm
50 μm
H
Visual cortex
RPTPδ/Tubb3/DAPI
RPTPδ
Tubb3
RPTPδf/f
RPTPδf/f;RORαcre
50 μm
50 μm
I
dLGN
DapB/DapB/DAPI
DapB neg control
DapB neg control
RPTPδf/f;RORαcre
50 μm
J
RPTPδ f/f; RORαcre
HC
VC
Thal
f/f
f/f;cre
f/f
f/f;cre
f/f
f/f;cre
150
100
75
50
K
HEK cell lysates
Empty lane
Untransfect
Hu LAR
Hu RPTPδ
Ms RPTPδ
Hu RPTPσ
Rt RPTPσ
RPTPδ
β-Tubulin

Fig. S7

Figure S7 (Relates to Figure 7). RPTPδ KO mice have increased overlap of NP1 with presynaptic terminals in the developing visual system *in vivo*. A. Diagram: structure of RPTPδ adapted from (Takahashi and Craig, 2013) showing the flox target region (D1 phosphatase domain), as well as the FN3 domain (target of the antibody). **B-C.** Validation of Pax6-a-cre line, demonstrating expression of cre recombinase in the developing retina. **B.** Pax6-a-cre was crossed to an eGFP reporter line to visualize regions of cre expression in the retina. GFP positive signal was observed in the ganglion cell layer (GCL), inner plexiform layer (IPL), a subset of cells in the inner nuclear layer (INL) and in the outer plexiform layer (OPL). Scale bar = 200 μm. GFP single channel inset shows a zoomed in image of the box. Arrow points to the ganglion cell layer neurons positive for GFP expression. Scale bar = 50 μm. **C.** Decrease in the amount of RPTPδ protein in retina lysates from RPTPδ fl/fl Pax6-α-cre +ve mice detected by western blot using the specific anti-RPTPδ antibody. Only the C isoform (~150 KDa bands) is expressed in the retina (Shishikura et al., 2016). 20μg retinal protein lysate was loaded in each lane. An example blot is shown on the left and quantification of band intensities is on the right. β-Tubulin antibody was used as loading control (bottom panel) shown in a separate blot. Graph shows mean±s.e.m., number of mice per genotype inside the bar. Statistical analysis by t-test, P value shown on the graph. **D-F.** Validation of RORα cre line, demonstrating expression of cre recombinase in the developing thalamus including thalamocortical projection neurons. RORα cre was crossed to a tdTomato reporter line to visualize regions of cre expression. **D.** Low power image of RORα cre +ve; tdTomato +ve forebrain at P6, tdTomato red and DAPI to mark nuclei in blue. Arrows mark tdTomato expression in the dLGN and thalamocortical axon projections in the visual cortex. Scale bar = 200μm. Inset shows zoomed in region of dLGN neurons expressing tdTomato. Scale bar = 20μm. **E-F.** Colabeling of thalamocortical projections in RORα cre mouse marked with tdTomato expression, along with presynaptic excitatory markers VGlut2 and VGlut1. Low power image shows all cortical layers. Scale bar = 20μm, inset shows boxed region in layer 4. Scale bar = 10μm. Arrows show thalamoroctical projections to layer 4. **E.** Extensive overlap of tdTomato expressing axon terminals with the thalamocortical presynaptic marker VGlut2. TdTomato in red, VGlut2 in green, DAPI in blue. Right panels show single channel images as labeled. Arrowheads mark example colocalized puncta. **F.** No overlap of tdTomato expressing axon terminals with the intracortical presynaptic marker VGlut1. TdTomato in red, VGlut1 in green, DAPI in blue. Right panels show single channel images as labeled. **G-I** RPTPδ mRNA is expressed in the developing visual system, and the mRNA levels are reduced in the thalamus but not in the cortex when crossing RPTPδ fl/fl mice to RORα cre line. Double ISH for RPTPδ mRNA along with the neuronal marker Tubb3 in the developing visual system at P6. **G.** RPTPδ expression in RPTPδ fl/fl cre –ve (top) and RPTPδ fl/fl RORα cre +ve (bottom) in dLGN. **H.** RPTPδ expression in RPTPδ fl/fl cre –ve (top) and RPTPδ fl/fl RORα cre +ve (bottom) in the VC. RPTPδ in red; Tubb3 in green, and DAPI to mark cell nuclei in blue. Scale bar = 50μm. **I.** No signal was detected using the negative control probe. **J.** Decrease of the amount of RPTPδ protein in thalamus lysates of RPTPδ fl/fl RORα cre +ve mice detected by western blot using the specific anti-RPTPδ antibody (compare lanes marked Thal ~80KDa bands; D isoform). No change in the amount of RPTPδ in hippocampal or cortical lysates (compare HC and VC lanes ~80KDa bands). C and D isoforms are detected in the thalamus, only D isoforms are observed in HC and VC. Note decrease only in D isoform level in the thalamus. **K.** To confirm the specificity of the anti-RPTPδ antibody for RPTPδ over σ and LAR, HEK cells were transfected with different RPTPs and lysates probed by western blot. 10μg lysate was loaded in each lane, β-Tubulin antibody was used as loading control (bottom panel) shown in a separate blot. Anti RPTPδ antibody successfully detected mouse (Ms) and human (Hu) RPTPδ in HEK cell lysate and did not detect the other family members LAR or RPTPs, human or rat (Rt). Samples shown in **J** and **K** were probed on the same blot.

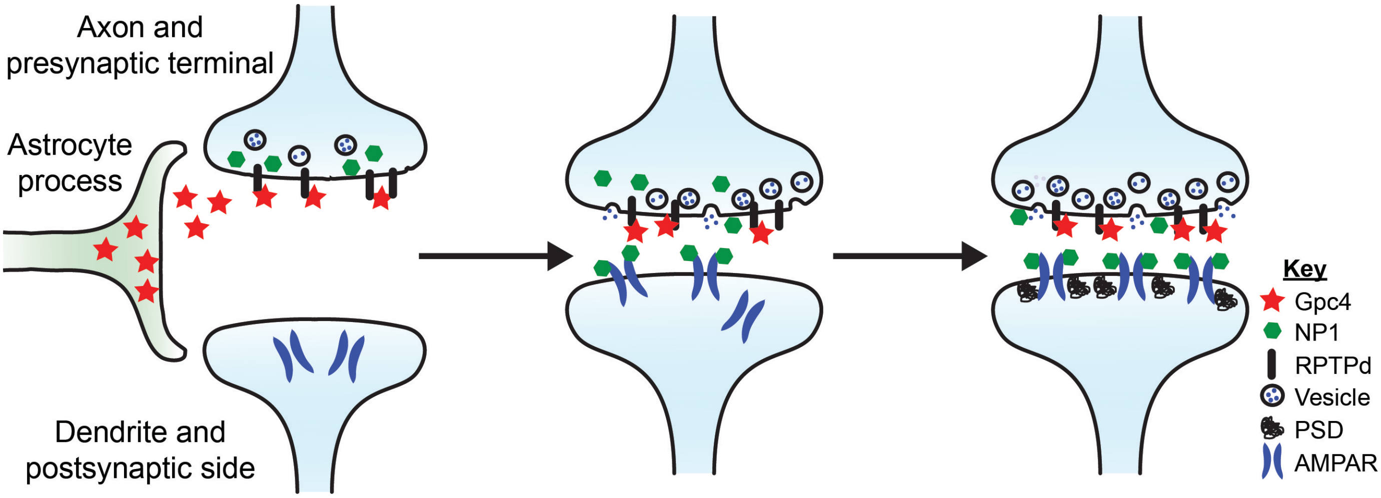
Axon and
presynaptic terminal
Astrocyte
process
Dendrite and
postsynaptic side
Key
Gpc4
NP1
RPTPd
Vesicle
PSD
AMPAR

Fig. S8

Figure S8. Model of Gpc4 mechanism of action. Astrocyte-secreted glypican 4 (Gpc4) binds to RPTPδ on axons, leading to release of the AMPA receptor clustering factor neuronal pentraxin 1 (NP1) from presynaptic terminals. NP1 binds to the extracellular region of GluA1 AMPARs on nearby dendrites, stabilizing them on the dendritic surface and inducing nascent synapse formation.

Gene Symbol	Description	Alone (log2)	Gpc4 (log2)	TSP1 (log2)	Gpc4 vs Alone Fold	Gpc4 vs Alone p	TSP1 vs Alone Fold	TSP1 vs Alone p
Gria1	Glutamate receptor, ionotropic, AMPA 1	7.24	7.26	7.20	1.01	0.85	-1.03	0.75
Gria2	Glutamate receptor, ionotropic, AMPA 2	10.63	10.38	10.54	-1.19	0.14	-1.06	0.12
Gria3	Glutamate receptor, ionotropic, AMPA 3	6.77	6.65	6.72	-1.09	0.36	-1.04	0.75
Gria4	Glutamate receptor, ionotropic, AMPA 4	8.95	8.97	9.05	1.01	0.92	1.07	0.62
Cacng2	Stargazin	9.06	8.72	8.83	-1.26	0.25	-1.17	0.16
**Cacng3**	TARP gamma 3	7.52	6.99	7.21	***-1.44***	***0.047***	-1.25	0.20
Cacng4	TARP gamma 4	5.61	5.59	5.50	-1.02	0.90	-1.08	0.65
**Cacng5**	TARP gamma 5	4.37	4.63	4.50	***1.20***	***0.041***	1.10	0.40
Cacng7	TARP gamma 7	5.49	5.64	5.39	1.11	0.08	-1.07	0.22
Cacng8	TARP gamma 8	4.51	4.40	4.33	-1.08	0.66	-1.13	0.47
Cdh2	N cadherin	10.53	10.44	10.59	-1.06	0.26	1.04	0.46
Cnih	Cornichon	11.03	11.10	11.05	1.05	0.45	1.01	0.52
Cnih2	Cornichon 2	9.98	9.95	9.91	-1.02	0.40	-1.05	0.54
Cnih4	Cornichon 4	8.33	8.36	8.47	1.02	0.78	1.10	0.23
Dlg1	SAP97	6.56	6.64	6.46	1.06	0.31	-1.07	0.38
Dlg3	SAP102	5.02	5.05	5.22	1.02	0.80	1.15	0.35
Dlg4	PSD95	9.15	9.14	9.10	-1.01	0.21	-1.04	0.49
Epb41l1	4.1N	9.89	9.84	9.85	-1.03	0.67	-1.02	0.72
Grip1	Glutamate receptor interacting protein 1	9.19	9.12	9.32	-1.05	0.07	1.09	0.43
**Grip2**	Glutamate receptor interacting protein 2	6.15	6.18	6.18	1.02	0.81	***1.03***	***0.040***
**Nptx1**	Neuronal pentraxin I	11.31	12.18	11.23	***1.83***	***0.00029***	-1.06	0.69
Nptxr	Neuronal pentraxin receptor	10.72	10.74	10.76	1.02	0.11	1.03	0.30
Nsf	N-ethylmaleimide-sensitive factor	11.67	11.67	11.60	-1.00	0.98	-1.05	0.42
Pick1	Protein interacting with PRKCA 1	9.02	8.98	8.95	-1.03	0.53	-1.05	0.33
**Syndig1**	Synapse differentiation inducing 1	6.81	7.65	6.74	***1.79***	***0.010***	-1.06	0.53
**Tspan7**	Tetraspanin 7	12.17	12.20	12.20	1.02	0.10	**1.02**	**0.05**

Table S2 (Relates to Figure 1). Expression level of genes related to AMPAR trafficking and clustering in RGC neurons that are untreated or treated with Gpc4 or TSP1 for 12 hours, taken from full Affymetrix microarray data set in Table S1. Candidate genes significantly altered ($P \leq 0.05$) by Gpc4 or TSP1 treatment are shown in bold.

Receptor	Probe Set ID	Alone	Gpc4	TSP1
RPTPδ	1374591_at	564	522	590
LAR	1368036_at	1194	1088	1126
RPTPσ	1387901_at	3236	2945	2880

Table S3 (Relates to Figure 3). Expression level of type 2a RPTP family members in rat RGC neurons *in vitro*, taken from full Affymetrix microarray data set in Table S1.
