# Supplementary material for: Astrocyte-Secreted Glypican 4 Regulates Release of Neuronal Pentraxin 1 from Axons to Induce Functional Synapse Formation
Source: Neuron. 2017 Oct 11;96(2):428–445.e13. doi: 10.1016/j.neuron.2017.09.053 (PMC5663462; doi:10.1016/j.neuron.2017.09.053)
Supplement: Document S2. Article plus Supplemental Information [file mmc3.pdf]

Neuron

Article

Astrocyte-Secreted Glypican 4 Regulates Release of Neuronal Pentraxin 1 from Axons to Induce Functional Synapse Formation

Highlights

- Astrocyte-secreted Gpc4 induces release of NP1 from neurons
- Release of NP1 is mediated through Gpc4 interaction with presynaptic RPTPδ
- Gpc4 or RPTPδ KO causes presynaptic NP1 retention and decreased synapse number
- Astrocytic release of Gpc4 provides spatial and temporal cues for synaptogenesis

Authors

Isabella Farhy-Tselnicker,
Adriana C.M. van Casteren,
Aletheia Lee, Veronica T. Chang,
A. Radu Aricescu, Nicola J. Allen

Correspondence

nallen@salk.edu

In Brief

Astrocytes are important regulators of synapse formation. Farhy-Tselnicker et al. identify the molecular pathway of active neuronal synapse formation induced by astrocyte-secreted glypican 4. These data functionally link astrocytes to signaling cascades activated in both pre- and postsynaptic neurons.

Farhy-Tselnicker et al., 2017, Neuron *96*, 428–445
October 11, 2017 © 2017 MRC Laboratory of Molecular Biology.
Published by Elsevier Inc.
https://doi.org/10.1016/j.neuron.2017.09.053

Neuron
Article

Astrocyte-Secreted Glypican 4 Regulates Release of Neuronal Pentraxin 1 from Axons to Induce Functional Synapse Formation

Isabella Farhy-Tselnicker,[1] Adriana C.M. van Casteren,[1] Aletheia Lee,[2] Veronica T. Chang,[3] A. Radu Aricescu,[2,3] and Nicola J. Allen[1,4,*]
[1]Salk Institute for Biological Studies, Molecular Neurobiology Laboratory, 10010 North Torrey Pines Rd, La Jolla, CA 92037, USA
[2]University of Oxford, Wellcome Trust Centre for Human Genetics, Division of Structural Biology, Roosevelt Drive, Oxford OX3 7BN, UK
[3]MRC Laboratory of Molecular Biology, Neurobiology Division, Francis Crick Avenue, Cambridge Biomedical Campus, Cambridge CB2 0QH, UK
[4]Lead Contact
*Correspondence: nallen@salk.edu
https://doi.org/10.1016/j.neuron.2017.09.053

SUMMARY

The generation of precise synaptic connections between developing neurons is critical to the formation of functional neural circuits. Astrocyte-secreted glypican 4 induces formation of active excitatory synapses by recruiting AMPA glutamate receptors to the postsynaptic cell surface. We now identify the molecular mechanism of how glypican 4 exerts its effect. Glypican 4 induces release of the AMPA receptor clustering factor neuronal pentraxin 1 from presynaptic terminals by signaling through presynaptic protein tyrosine phosphatase receptor δ. Pentraxin then accumulates AMPA receptors on the postsynaptic terminal forming functional synapses. Our findings reveal a signaling pathway that regulates synaptic activity during central nervous system development and demonstrates a role for astrocytes as organizers of active synaptic connections by coordinating both pre and post synaptic neurons. As mutations in glypicans are associated with neurological disorders, such as autism and schizophrenia, this signaling cascade offers new avenues to modulate synaptic function in disease.

INTRODUCTION

During central nervous system (CNS) development, the formation of neuronal synapses at the right time, in the right place, and of the right strength is crucial to the ongoing function of the brain throughout life. Work in recent years has shown that secreted signals from neighboring astrocytes strongly regulate synapse formation, affecting not only when and where synapses would form, but also whether an active or a silent synapse will develop (Allen, 2013; Ullian et al., 2001). Silent synapses are induced by astrocyte-secreted thrombospondins and hevin, each regulating distinct molecular pathways in neurons (Christopherson et al., 2005; Eroglu et al., 2009; Kucukdereli et al., 2011; Singh et al., 2016). We previously showed that astrocyte-secreted glypicans 4 and 6 (Gpc4 and Gpc6) induce active synapse formation by recruiting dendritic GluA1-containing AMPA glutamate receptors (AMPARs) (Allen et al., 2012); however, the neuronal signaling pathways that underlie this effect have not been determined. Here we identify the signaling pathway induced in neurons by astrocyte-secreted Gpc4, leading to functional synapse formation.

GluA1, the AMPAR subunit regulated by Gpc4 and Gpc6, is a major component of AMPARs at developing synapses, suggesting an important role for Gpc4 in synapse initiation (Allen et al., 2012). It has been proposed that, in developing neurons, AMPARs are present at nascent synaptic contacts but in an unstable state and that the stabilization of AMPARs is crucial for the maintenance and maturation of these synapses (Groc et al., 2006). A major way of regulating synaptic AMPAR stability and function is via AMPAR-interacting proteins that can act both intra- and extracellularly (Bassani et al., 2013; Henley and Wilkinson, 2016). An important family of extracellular regulators are the neuronal pentraxins (NP): NP1, NP2 (also known as NARP), and NPR (Lee et al., 2017; O'Brien et al., 1999; Sia et al., 2007; Xiao et al., 2017; Xu et al., 2003). These secreted glycoproteins bind to AMPARs and stabilize them on dendritic surfaces (O'Brien et al., 1999). Removal of all NPs from developing neurons leads to severe delays in active synapse formation *in vitro*, and loss of neuronal pentraxins *in vivo* causes defects in synapse maturation and in the activity-dependent refinement of circuits (Bjartmar et al., 2006; Koch and Ullian, 2010; Sia et al., 2007). NP1 is most highly expressed in the developing brain when synapses are first forming (Bjartmar et al., 2006). In contrast to NP2, which is an immediate early gene whose mRNA is known to be regulated by neuronal activity (Xu et al., 2003), the mechanisms that regulate release of NP1 from neurons are not known.

Type 2a receptor protein tyrosine phosphatases (RPTPs) and leucine-rich repeat transmembrane proteins (LRRTMs) can act as neuronal receptors for glypican family members, but whether they mediate the effects of astrocyte-secreted Gpc4 is unknown (Coles et al., 2011; de Wit et al., 2013; Johnson et al., 2006; Ko et al., 2015; Siddiqui et al., 2013; Takahashi and Craig, 2013).

 © 2017 MRC Laboratory of Molecular Biology. Published by Elsevier Inc.
This is an open access article under the CC BY license (http://creativecommons.org/licenses/by/4.0/).

Postsynaptic LRRTM3 and LRRTM4 interact with a neuronal membrane-tethered Gpc4 via *trans*-synaptic binding to regulate synapse formation (de Wit et al., 2013; Ko et al., 2015; Siddiqui et al., 2013). Presynaptic RPTPs interact with glypicans to regulate axon extension (Coles et al., 2011) and promote synapse formation via *trans*-synaptic interaction with surface receptors and cell adhesion molecules (Coles et al., 2014; Dunah et al., 2005; Takahashi et al., 2011; Takahashi and Craig, 2013). Mice lacking RPTPs or LRRTMs show deficits in synapse formation, making them strong candidates for mediating the actions of astrocyte-secreted Gpc4 (Horn et al., 2012; Meathrel et al., 2002; Siddiqui et al., 2013; Uetani et al., 2000). Importantly, the mechanism of how synapses are formed downstream of Gpc4-receptor interaction is not known.

Here we identify the pathway of astrocyte-secreted Gpc4 in inducing functional synapse formation. We found that Gpc4 induces release of the AMPAR-clustering factor NP1 from axons by interacting with presynaptic RPTPδ. The secreted NP1 binds and recruits postsynaptic GluA1-containing AMPARs, promoting active synapses to form. Our findings reveal a functional link between pre- and postsynaptic signaling pathways and provide important mechanistic insight into the role of astrocytes in neuronal synaptogenesis. Mutations in glypican family members have been implicated in multiple neurological disorders that have underlying synaptic dysfunction, including autism spectrum disorder (ASD), attention-deficit hyperactivity disorder (ADHD), and schizophrenia (Doan et al., 2016; Lesch et al., 2008; Potkin et al., 2010). Identification of the molecular mechanism of how glypicans regulate synapse formation, and their unexpected functional link with NP1, opens new avenues to explore the mechanisms of these disorders.

RESULTS

Glypican 4 Regulates Neuronal Expression of Genes Related to AMPAR Synaptic Localization

Due to the relatively slow effect of Gpc4 on GluA1 synaptic expression (Allen et al., 2012), we hypothesized that Gpc4 regulates neuronal signaling pathways that mediate AMPAR dendritic clustering. To test this, we took an unbiased approach—gene expression profiling of neurons treated with soluble Gpc4 protein to mimic the astrocyte-secreted form, using pure cultures of retinal ganglion cell neurons (RGCs) (Ullian et al., 2001). RGCs are ideal for these studies as they form few synapses when cultured in isolation but profoundly increase synapse number and function when cultured with astrocytes or astrocyte-secreted proteins, including Gpc4 (Allen et al., 2012). RGCs were treated for 12 hr (preceding the surface GluA1 increase; Allen et al., 2012) with Gpc4 to induce active synapse formation or thrombospondin 1 (TSP1) to induce silent synapse formation (Figure 1A), and mRNA levels were measured using microarrays. TSP1 is an additional control as it induces structurally intact, but functionally silent, AMPAR-lacking synapses by signaling through the α2δ1 receptor (Christopherson et al., 2005; Eroglu et al., 2009), so it is not predicted to use the same mechanism as Gpc4. There was no overlap in gene expression changes induced by Gpc4 (49 genes) and TSP1 (3 genes), highlighting that they induce synapse formation via distinct pathways (Table S1). We focused on factors known to regulate synaptic clustering of AMPARs and found that, interestingly, mRNA for neuronal pentraxin 1 (NP1) is significantly upregulated by Gpc4 treatment (1.83-fold, Table S2).

Glypican 4 Upregulates Release of the AMPAR Clustering Factor NP1 from Neurons

NP1 binds the extracellular region of AMPARs and clusters them on dendritic surfaces increasing synapse number (O'Brien et al., 1999; Xu et al., 2003). This same effect is seen in neurons treated with soluble Gpc4 (Allen et al., 2012), making regulation of NP1 an ideal candidate for Gpc4-induced synaptogenesis. Since NP1 is a secreted protein that acts in the extracellular space, we first asked whether Gpc4 would increase NP1 protein secretion (Figure 1B). RGCs were treated with soluble Gpc4 for 4 days, and levels of NP1 in the neuron culture media were assayed by western blot. Gpc4 treatment caused a significant increase in the extracellular levels of NP1 (2.07-fold ± 0.10-fold), and to a lesser extent NP2 (1.41-fold ± 0.10-fold; Figures S1A and S1B), showing that Gpc4 is sufficient to upregulate NP1 release from neurons. To determine whether secreted NP1 is binding to the surface of neuronal processes, which is necessary for it to cluster AMPARs (Xu et al., 2003), we examined the levels of NP1 using a surface immunostaining assay. Treating RGCs with astrocyte conditioned media (ACM) or purified soluble Gpc4 for 6 days increased the amount of NP1 accumulated on the surface of neuronal processes by 1.54-fold ± 0.16-fold and 1.84-fold ± 0.22-fold for ACM and Gpc4, respectively (Figures 1C and 1D). The same results were obtained using two different antibodies against NP1 (Figures S1C–S1F). To test the specificity of this effect, we treated RGCs with TSP1 to induce silent synapses that lack AMPARs (Figure 1A). TSP1 treatment did not increase surface levels of NP1 over untreated controls (1.18-fold ± 0.11-fold, ns; Figures 1E and 1F), showing that NP1 release is specific to the Gpc4 pathway.

To determine when an increase in NP1 surface accumulation can first be detected, we performed a time course experiment. At all times tested, both ACM and Gpc4 caused an increase in surface NP1 compared to untreated neurons. Measurements were performed at 4 hr (ACM: 1.72-fold ± 0.17-fold; Gpc4: 1.64-fold ± 0.15-fold), 12 hr (ACM: 2.10-fold ± 0.22-fold; Gpc4: 1.87-fold ± 0.26-fold), and 6 days (above) (Figures 1C, 1D, and 1G–1J). Because increases in NP1 surface accumulation preceded our 12-hr microarray time point, we used qRT-PCR to determine when a significant change in NP1 mRNA is detected. We found that NP1 mRNA was beginning to be upregulated compared to untreated RGCs after 4 hr (1.28-fold ± 0.27-fold, ns) and significantly upregulated after 12 hr (1.23-fold ± 0.07-fold) and 18 hr (1.56-fold ± 0.11-fold), but not after 1 hr (1.09-fold ± 0.06-fold, ns) (Figure S1G). Gpc4 treatment did not alter the mRNA expression levels of the other NPs (NP2 and NPR) at any time point assayed (Figure S1G). To determine whether an increase in NP1 mRNA is necessary for the increase in surface accumulation of NP1 protein (Figures 1C, 1D, and 1G–1J), we treated RGCs with actinomycin D (ActD) to block mRNA synthesis, and asked whether Gpc4 was still able to increase surface NP1, assayed by immunostaining. ActD had no effect on the ability of Gpc4 to increase surface NP1 after 4 hr (Gpc4: 2.54-fold ± 0.27-fold; Gpc4+ActD: 2.32-fold ± 0.21-fold)

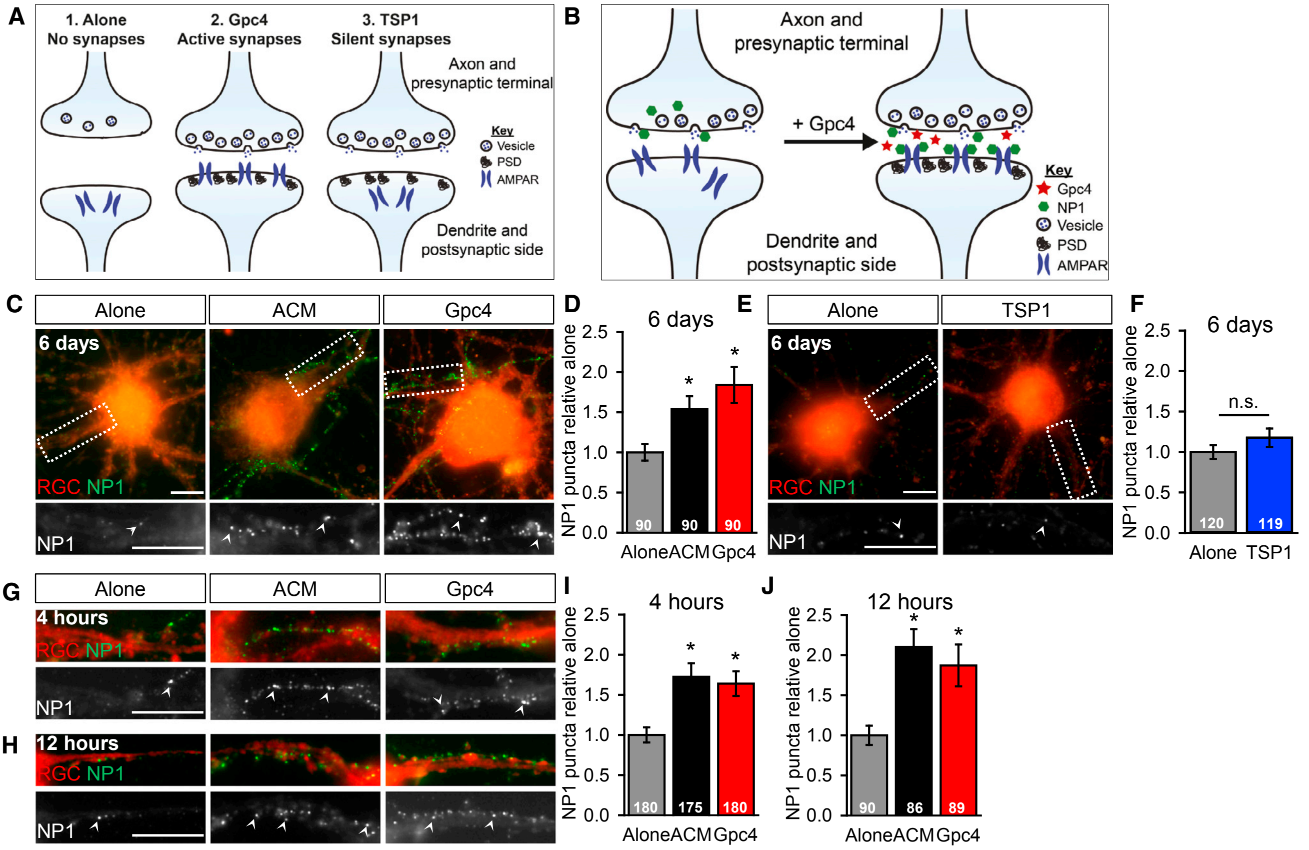

Figure 1. Gpc4 Upregulates Release of the AMPAR Clustering Factor NP1 from RGC Neurons

(A) Model of experiment: RGCs were cultured alone or with Gpc4 to induce AMPARs containing active synapses or with TSP1 to induce AMPARs lacking silent synapses.

(B) Diagram: Gpc4-induces presynaptic release of NP1, with NP1 binding to postsynaptic AMPARs and inducing synapse formation.

(C and D) RGCs treated with ACM or Gpc4 for 6 days show increased surface accumulation of NP1. (C) Example images: green surface NP1, red labels whole cell. Inset shows enlarged dendritic region from box, surface NP1 white. (D) Quantification of (C), number of surface NP1 puncta per RGC normalized to Alone condition, n = 3 experiments.

(E and F) TSP1 does not increase surface NP1. (E) Example images: as in (C). (F) Quantification of (E), number of surface NP1 puncta per RGC normalized to Alone condition, n = 4 experiments.

(G–J) Time course of surface accumulation of NP1 in response to ACM or Gpc4 treatment for 4 or 12 hr. (G and H) Example dendrite images, green surface NP1, red labels whole process; below single channel NP1, white. (I and J) Quantification of (G) and (H), number of surface NP1 puncta per RGC normalized to Alone condition, n = 6 experiments 4 hr, 3 experiments 12 hr. Scale bars, 10 μm. Arrowheads mark example puncta of NP1. Graphs show mean ± SEM, number of cells per group inside the bar. *p ≤ 0.05, by t test when comparing two groups or one-way ANOVA when comparing three or more groups.

See also Figure S1.

(Figures S1H and S1J), but it blocked the increase in NP1 following 12 hr of Gpc4 treatment compared to untreated RGCs (Gpc4: 2.05-fold ± 0.24-fold; Gpc4+ActD: 0.81-fold ± 0.07-fold; Figures S1I and S1K). These results demonstrate that the initial step of Gpc4-induced synaptogenesis is increasing secretion and surface accumulation of NP1 protein, rather than regulation of NP1 mRNA levels, suggesting that a local synaptic signaling mechanism may initiate NP1 protein secretion. This possibility is explored in the following experiments.

Interaction between NP1 and GluA1 Is Necessary for Glypican 4 to Induce Synapse Formation

We previously demonstrated that surface clustering of GluA1 is necessary for Gpc4 to induce synapse formation (Allen et al., 2012). NP1 induces synapse formation by binding to AMPARs (Lee et al., 2017; Xu et al., 2003), so we next asked whether blocking the NP1-GluA1 interaction would prevent Gpc4-induced synaptogenesis. To do this, we generated a Fab antibody fragment that specifically binds the amino-terminal domain (ATD) of GluA1 (GluA1-Fab), the NP1 binding site, but not to other AMPAR subunits (Figure 2A). Specific binding was confirmed by surface plasmon resonance analysis (Figure 2B) and by immunostaining HEK293T cells overexpressing different AMPAR subunits with GluA1-Fab, showing binding to GluA1, but not GluA2, GluA3, or GluA4, (Figure S2A). As a control, we mutated the GluA1-Fab CDR region, generating the Mut-Fab variant, which does not bind GluA1 (Figure S2B). To determine whether the GluA1-Fab can block NP1 binding to RGC

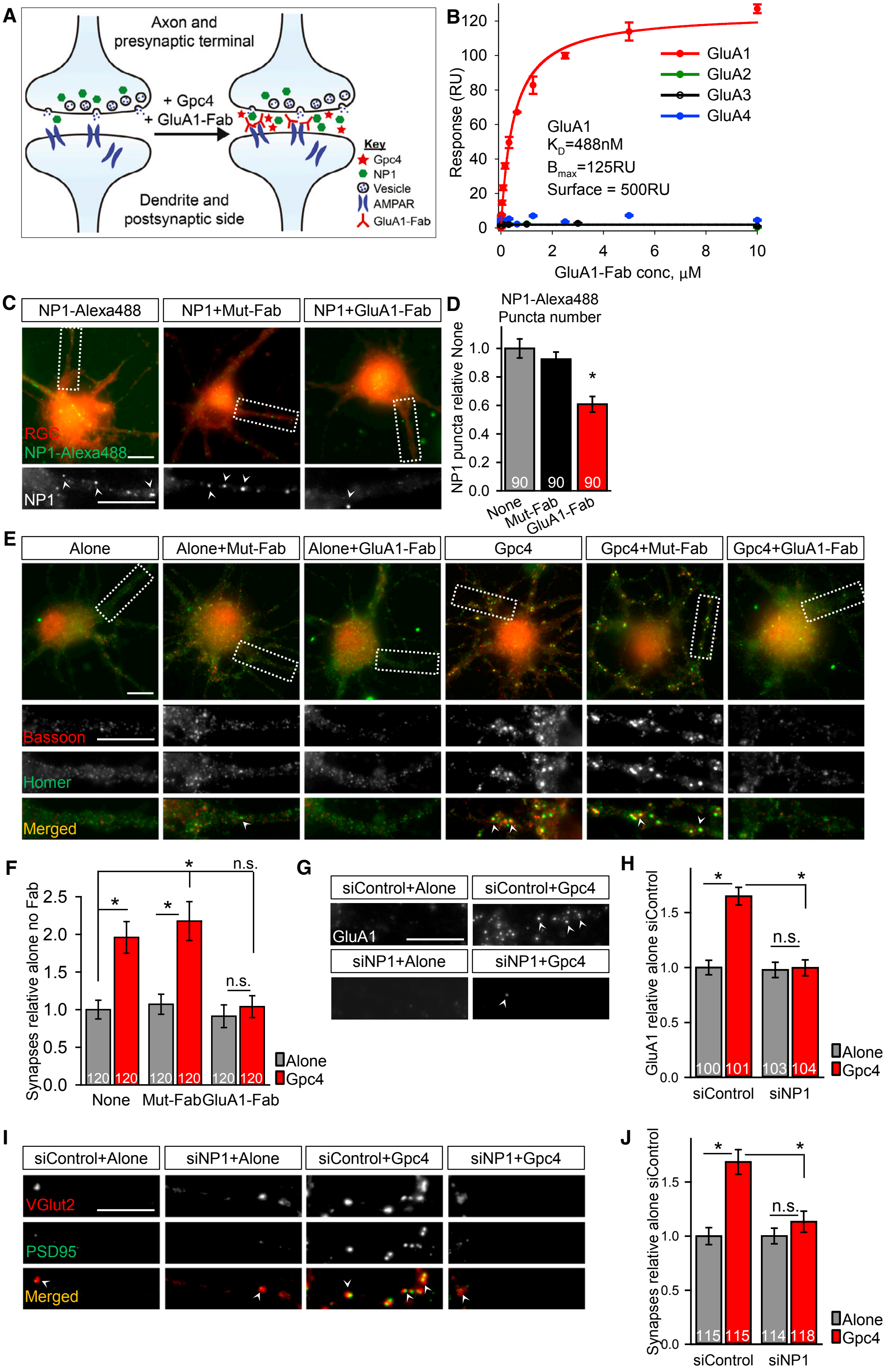

(legend on next page)

processes, we applied recombinant NP1 labeled with Alexa 488 to RGCs *in vitro* for 24 hr, either by itself or in the presence of the Mut-Fab or GluA1-Fab. NP1-Alexa 488 showed robust binding to RGC processes (Figure 2C), and this was significantly decreased by the presence of the GluA1-Fab, whereas the Mut-Fab had no effect (Mut-Fab: 0.92-fold ± 0.05-fold; GluA1-Fab: 0.61-fold ± 0.05-fold; compared to NP1-Alexa 488: no treatment; Figures 2C and 2D). We next asked whether the GluA1-Fab could inhibit Gpc4-mediated synapse formation. RGCs were treated for 6 days with soluble Gpc4 along with the GluA1-Fab or the Mut-Fab (Figure 2A). Synapse formation was assayed by immunostaining for Bassoon (presynaptic active zone) and Homer (postsynaptic density), with colocalization of these markers counted as structural synapses (Allen et al., 2012; Eroglu et al., 2009). The presence of the GluA1-Fab prevented synapse formation in response to Gpc4 (1.04-fold ± 0.15-fold; ns), whereas the Mut-Fab had no effect (2.18-fold ± 0.26-fold; Figures 2E and 2F).

As an alternative approach to determine necessity of NP1 for Gpc4-induced synapse formation, we used small interfering RNA (siRNA) to knock down NP1 expression in RGCs. Transfection of RGCs with siNP1 caused a significant 76% decrease in surface accumulation of NP1 protein compared to a non-targeting siControl (Figures S2C and S2D), confirming the efficacy of the siRNA. We then asked whether soluble Gpc4 was able to increase surface clustering of GluA1 in RGCs transfected with siNP1 compared to siControl by immunostaining using an antibody specific for the extracellular region of GluA1 (Allen et al., 2012). Transfection of RGCs with siNP1 prevented Gpc4-mediated increase in surface GluA1 (0.99-fold ± 0.07-fold), whereas siControl did not (1.65-fold ± 0.08-fold; Figures 2G and 2H). Next, we asked whether RGCs transfected with siNP1 could form structural synapses in response to Gpc4 by immunostaining RGCs for vesicular glutamate transporter type 2 (VGlut2 - presynaptic vesicles) and PSD95 (postsynaptic density). Transfection of RGCs with siNP1 prevented Gpc4-mediated synapse formation (1.13-fold ± 0.09-fold), whereas the siControl did not (1.68-fold ± 0.11-fold; Figures 2I and 2J). These data demonstrate that soluble Gpc4 regulates synaptic recruitment of GluA1 and synaptogenesis by increasing the release of NP1, which binds GluA1 AMPARs to induce synapse formation. NP1 is released from presynaptic terminals (Sia et al., 2007), suggesting that Gpc4 acts by interacting with a presynaptic receptor, an unexpected finding for an astrocyte-secreted factor that increases surface clustering of postsynaptic GluA1 AMPARs.

Presynaptic RPTPδ Mediates Glypican 4-Induced Synapse Formation

Previous work has identified type 2a RPTPs as potential presynaptic receptors for glypicans—specifically RPTPσ and LAR (Coles et al., 2011; Johnson et al., 2006; Ko et al., 2015). Rodent RGCs express mRNA for all three type 2a RPTPs: σ, δ, and LAR (Table S3; Brooks et al., 2013), making them strong candidates for mediating the effect of astrocyte-secreted Gpc4 (Figure 3A). Due to potential functional redundancy between the RPTPs, we first asked whether overexpression of RPTPs *in vitro* would increase synapse formation in response to the putative ligand Gpc4. We first tested whether overexpression of RPTPs in the presynaptic compartment (i.e., axons) could enhance Gpc4-induced synapse formation (Figure 3B). Axons expressing each RPTP were identified by immunostaining for the hemagglutinin (HA)-tag (in the receptor), and synapses were stained with Bassoon and PSD95 (Figure 3C). Overexpression of LAR or RPTPσ in the axon did not enhance Gpc4-mediated synapse formation (Figures 3D–3G), although overexpression of RPTPσ was sufficient to increase synapse formation by itself in the absence of Gpc4 (1.65-fold ± 0.22-fold; Figures 3F and 3G). Overexpression of RPTPδ significantly increased synapse formation by itself (1.84-fold ± 0.25-fold; Figures 3H and 3I) and, importantly, further enhanced synapse formation in response to Gpc4 by 4.70-fold ± 0.56-fold (Figures 3H and 3I). We then asked whether overexpression of these receptors in the postsynaptic compartment (i.e., dendrites) could enhance Gpc4-induced synaptogenesis and found no significant effect (Figure S3A). These data demonstrate that axonal RPTPδ, but not RPTPσ or LAR, is sufficient to mediate the synaptogenic effect of Gpc4.

Blocking RPTP Interactions Prevents Glypican 4-Induced Synapse Formation

We next asked whether RPTP function is necessary for Gpc4-induced synapse formation. To test this, we used cell-permeant wedge domain-blocking peptides to alter RPTPs' interactions with their downstream targets (Xie et al., 2006). RPTPs are receptors for heparan sulfate proteoglycans (HSPGs), such as glypicans and chondroitin sulfate proteoglycans (CSPGs). CSPG-RPTP interactions inhibit axon outgrowth and block

Figure 2. NP1-GluA1 Interaction Is Necessary for Gpc4 to Induce Structural Synapse Formation

(A) Diagram: Fab against the N-terminal domain of GluA1 (GluA1-Fab) prevents Gpc4-induced synapse formation.

(B) Surface plasmon resonance analysis of GluA1-Fab specificity. GluA1-Fab binds GluA1 AMPAR N-terminal domain, but not GluA2, GluA3, or GluA4.

(C and D) Recombinant NP1-Alexa 488 binds RGC dendrites, and GluA1-Fab disrupts this binding, Mut-Fab has no effect. (C) Example images of RGCs treated with NP1-Alexa 488 (green), red labels whole cell. Inset shows enlarged dendritic region from box, surface NP1 white. Arrowheads mark example puncta of NP1. (D) Quantification of (C), number of surface NP1-Alexa 488 puncta normalized to NP1-Alexa 488 no Fab group (None). n = 3 experiments.

(E and F) Treating RGCs with GluA1-Fab blocks Gpc4-mediated synapse formation. (E) Example images of RGCs, red Bassoon, green Homer. Inset shows enlarged dendritic region from box. Arrowheads mark example synapses (colocalized Bassoon-Homer puncta). (F) Quantification of (E), number of synapses per RGC normalized to Alone, n = 4 experiments.

(G–J) Knockdown of NP1 by siRNA blocks Gpc4 effect on GluA1 surface clustering (G and H) and synapse formation (I and J). (G) Example images of RGC dendrites showing single-channel GluA1 puncta. Arrowheads mark example GluA1 surface puncta. (H) Quantification of (G), number of GluA1 puncta normalized to siControl+Alone. n = 4 experiments. (I) Example images of RGC processes stained for VGlut2 red, PSD95 green. Arrowheads mark example synapses (co-localized VGlut2-PSD95). (J) Quantification of (I), number of synapses per RGC normalized to siControl+Alone, n = 4 experiments. Scale bars, 10 μm. Graphs show mean ± SEM, number of cells per group inside the bar. *p ≤ 0.05, by one-way ANOVA.

See also Figure S2.

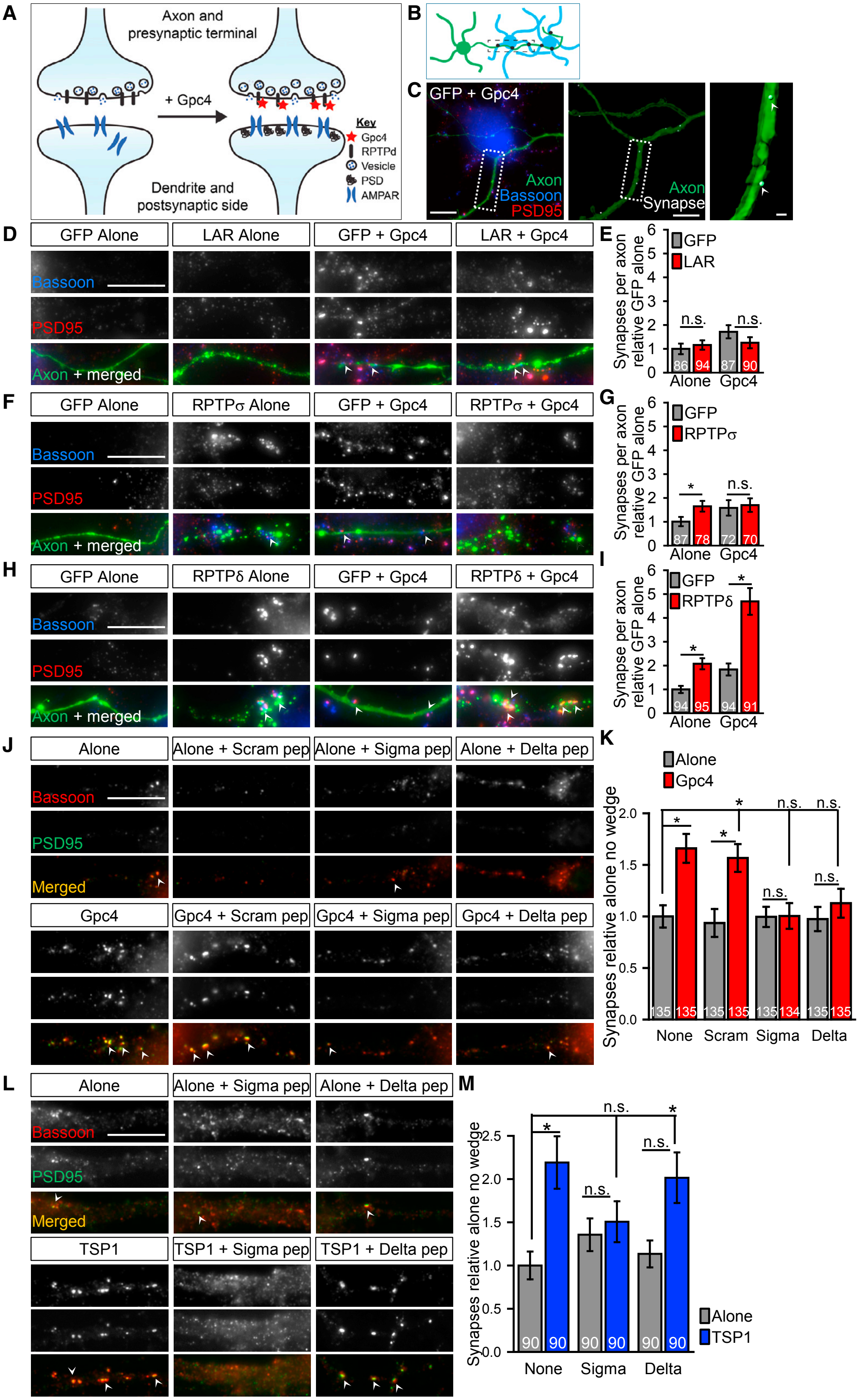

(legend on next page)

axon regeneration (Coles et al., 2011). Wedge peptides block the RPTP-mediated inhibitory effect of CSPGs on axon growth, demonstrating their functional efficacy (Lang et al., 2015). We asked whether peptides that specifically target RPTPσ, RPTPδ, or LAR, as well as a scrambled RPTPσ peptide, could block the synaptogenic actions of Gpc4. RGCs were treated with soluble Gpc4 for 6 days in the presence or absence of wedge domain-blocking peptides, and structural synapses were assayed by immunostaining for Bassoon and PSD95. The scrambled σ and LAR peptides did not affect Gpc4-induced synaptogenesis (Figures S3B and S3C). However, treating RGCs with peptides against RPTPσ or RPTPδ prevented Gpc4-mediated synaptogenesis (Gpc4: 1.66-fold ± 0.14-fold; Gpc4 + scrambled peptide: 1.57-fold ± 0.14-fold; Gpc4 + δ peptide: 1.13-fold ± 0.14-fold; Gpc4 + σ peptide: 1.01-fold ± 0.13-fold compared to no Gpc4 treatment; Figures 3J and 3K). To determine the specificity of RPTP function for Gpc4-mediated synaptogenesis, we treated RGCs with TSP1 to induce silent synapse formation in the presence or absence of the blocking peptides. Blocking RPTPσ reduced synapse formation in response to TSP1 (TSP1: 2.19-fold ± 0.30-fold; TSP1 + σ peptide: 1.51-fold ± 0.24-fold compared to no TSP1 treatment), whereas blocking RPTPδ had no effect (TSP1 + δ peptide: 2.01-fold ± 0.29-fold; Figures 3L and 3M). This shows that RPTPδ is necessary for Gpc4-mediated synaptogenesis and is specific to this pathway, whereas RPTPσ is necessary for synaptogenesis induced by multiple astrocyte-secreted factors.

Blocking RPTP Interactions Prevents Glypican 4-Induced GluA1 Clustering

A crucial step in Gpc4-mediated synaptogenesis is the surface clustering of GluA1 AMPARs (Allen et al., 2012), so we next asked whether RPTPδ or RPTPσ are involved in Gpc4-mediated GluA1 clustering (Figure 4A). RGCs were treated with Gpc4 and each blocking peptide, and surface clustering of GluA1 was measured by immunostaining. Blocking either RPTPσ or RPTPδ prevented the Gpc4-mediated increase in surface GluA1 (Gpc4: 2.30-fold ± 0.26-fold; Gpc4 + scrambled peptide: 1.97-fold ± 0.20-fold; Gpc4 + δ peptide: 1.13-fold ± 0.15-fold; Gpc4 + σ peptide: 1.34-fold ± 0.19-fold; each compared to no Gpc4 treatment; Figures 4B and 4C). These results demonstrate that presynaptic RPTPδ and RPTPσ are necessary for Gpc4 induction of GluA1 clustering in RGCs.

Blocking RPTP Interactions Prevents Glypican 4-Induced NP1 Surface Accumulation

We have demonstrated that soluble Gpc4 increases the release and surface accumulation of NP1 from neurons (Figures 1 and 2) and that Gpc4 acts through presynaptic RPTPδ and RPTPσ receptors to induce GluA1 clustering and synapse formation (Figures 3 and 4). To determine whether there is a functional link between these two pathways, we asked whether blocking RPTPδ or RPTPσ *in vitro* would prevent Gpc4-induced increases in NP1 surface accumulation (Figure 4A). RGCs were treated with Gpc4 and each blocking peptide, and surface levels of NP1 were measured using immunostaining. Inhibiting RPTPδ or RPTPσ prevented the Gpc4-mediated increase in NP1 surface accumulation (Figures 4D and 4E), linking together the RPTP and NP1 pathways.

Glypican 4 Is Expressed by Astrocytes throughout the Developing Visual System

What is the importance of this astrocyte-regulated presynaptic signaling cascade to synapse formation *in vivo*? We examined this question in the developing mouse visual system, as our *in vitro* work was conducted on retinal ganglion cell neurons (Figure S4D). We first confirmed that astrocytes are present in the visual system at postnatal day 6 (P6), when synapses are beginning to form (Ullian et al., 2001), using *in situ* hybridization (ISH) to detect mRNA for the astrocyte marker Aldh1l1 (Cahoy et al., 2008). A strong signal for Aldh1l1 was observed in the superior colliculus (SC) and dorsal lateral geniculate nucleus (dLGN), target regions of RGC axons *in vivo* (Figure 5A; Figure S4A). A similar signal was detected in the visual cortex (VC), a target of axons from the dLGN (Figure S4A). No signal was detected using the negative control probe (Figure 5A; Figure S4A, right). We then asked whether Gpc4 mRNA is expressed in these brain regions at P6. Gpc4 expression was detected in the SC, dLGN, and VC, and no signal for Gpc4 was detected in Gpc4 knockout (KO) mice (Figure 5B). To determine which cells express Gpc4 in these regions, we performed triple fluorescent ISH, combining Gpc4, an astrocyte marker (Aldh1l1), and a neuronal marker (Tubb3) (Figures 5C–5F; Figures S4B and S4C). We focused on the superficial layers of the SC, a target region of RGCs, and layer 4 of the VC, a target region of dLGN projections (Figures S4C and S4D). In the SC, we detected strong overlap between Gpc4 and Aldh1l1 and little overlap between Gpc4 and Tubb3

Figure 3. Glypican 4 Acts through Presynaptic RPTPs to Increase Synapse Formation

(A) Diagram: Gpc4 is acting through presynaptic RPTPs to induce synapse formation.

(B and C) Schematic of experiment performed in (D)–(I). (B) RPTPs are overexpressed in the axon (green cell), and synapses (black dots) formed by that axon onto neighboring dendrites (blue cells) are analyzed. (C) Example experiment with Imaris rendering to demonstrate analysis of synapses formed by the transfected axon. Left: raw image—transfected axon green, Bassoon blue, PSD95 red. Scale bar, 10 μm. Middle: Imaris rendering of green axon with synapses within that axon (white spheres represent colocalized pre- and postsynaptic puncta). Right: zoom in of rendered axon from box. Scale bar, 2 μm.

(D–I) Overexpression of RPTPδ in RGC axons enhances Gpc4-mediated synapse formation, no effect of RPTPσ, or LAR. (D, F, and H) Example images, axon green, Bassoon blue, PSD95 red. (E, G, and I) Quantification of (D), (F), and (H), respectively, synapse number per expressing axon normalized to GFP Alone group, n = 3 experiments each RPTP.

(J–K) Altering RPTPσ or RPTPδ function with wedge peptides blocks Gpc4-mediated synapse formation. (J) Example process images, Bassoon red, PSD95 green. (K) Quantification of (J), synapse number per cell normalized to Alone no peptide group, n = 5 experiments.

(L and M) Blocking RPTPσ with wedge peptides prevents TSP1-induced synapse formation, blocking RPTPδ has no effect. (L) Example images, Bassoon red, PSD95 green. (M) Quantification of (L), synapse number per cell normalized to Alone no peptide group, n = 3 experiments. Scale bars for (D)–(M), 10 μm. Arrowheads mark example synapses. Graphs show mean ± SEM, number of cells per group inside the bar. $^*p \leq 0.05$, by one-way ANOVA.

See also Figure S3.

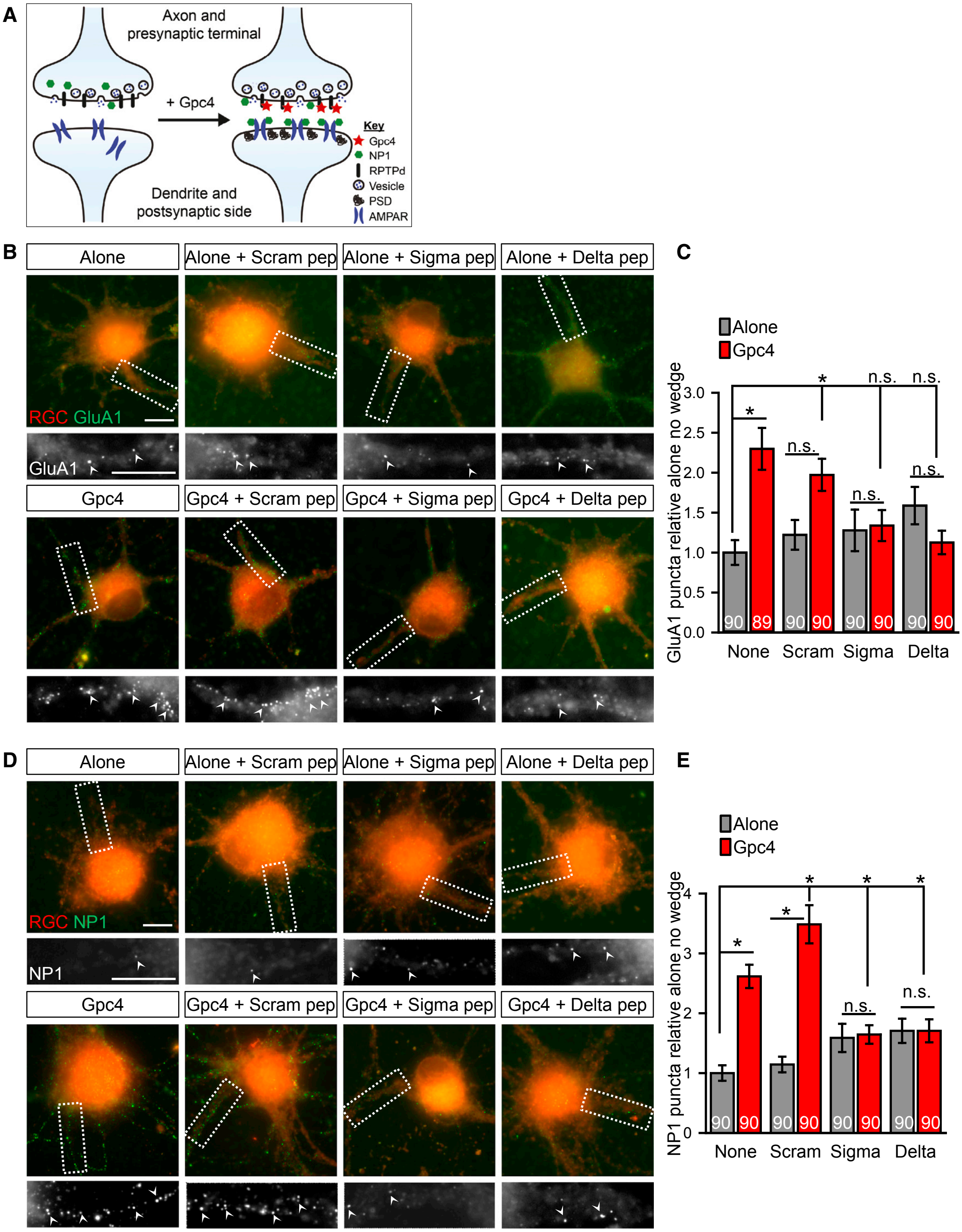

Figure 4. Glypican 4 Acts through Presynaptic RPTPs to Induce the Release of NP1 and to Cluster GluA1 AMPA Receptors

(A) Diagram: Gpc4 binds to presynaptic RPTPδ to induce the release of NP1, which binds postsynaptic AMPARs to induce synapse formation.

(B and C) Blocking RPTPσ or RPTPδ with wedge peptides prevents Gpc4-mediated surface clustering of GluA1. (B) Example images, GluA1 green, red labels whole cell. Inset shows enlarged dendritic region from box, surface GluA1 white. Arrowheads mark example clusters of GluA1. (C) Quantification of (B), number of GluA1 puncta per cell normalized to RGC alone no peptide group, n = 3 experiments.

(D and E) Blocking RPTPσ or RPTPδ with wedge peptides prevents Gpc4-mediated surface accumulation of NP1. (D) Example images, NP1 green, red labels whole cell. Inset shows enlarged dendritic region from box, surface NP1 white. Arrowheads mark example puncta of NP1. (E) Quantification of (D), number of NP1 puncta per cell normalized to RGC alone no peptide condition, n = 3 experiments. Scale bars, 10 μm. Graphs show mean ± SEM, number of cells per group inside the bar. *$p \leq 0.05$, by one-way ANOVA.

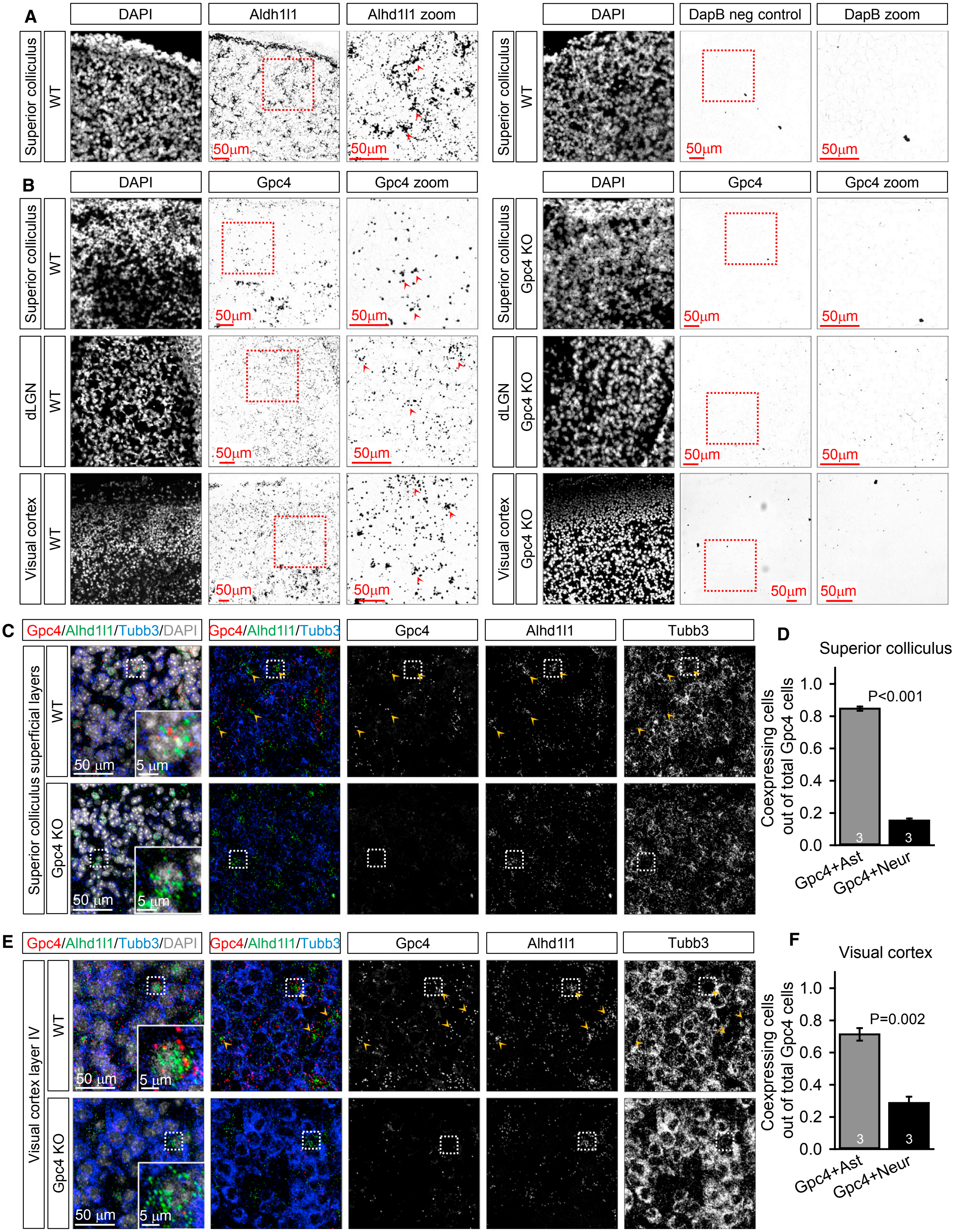

(legend on next page)

(fraction Gpc4+Aldh1l1 cells: 0.85 ± 0.01; Gpc4+Tubb3 cells: 0.15 ± 0.01; Figures 5C and 5D), with minimal signal detected for the negative control probe (Figure S4B). KO of Gpc4 resulted in minimal Gpc4 signal and no change in Aldh1l1 or Tubb3 signals, suggesting that KO of Gpc4 does not disrupt neuron or astrocyte formation (Figure 5C, bottom). Similar triple ISH results were obtained in the VC, showing higher overlap of Gpc4 with Aldh1l1 than with Tubb3 (Gpc4+Aldh1l1 cells: 0.71 ± 0.03; Gpc4+Tubb3 cells: 0.29 ± 0.04; Figures 5E and 5F). This confirms previous work from other brain regions showing elevated levels of Gpc4 expression in developing astrocytes compared to neurons (Allen et al., 2012; Cahoy et al., 2008) and shows that astrocytes express Gpc4 in the visual system at the time of synapse initiation.

Glypican 4 KO Mice Show Increased Retention of NP1 in Presynaptic Terminals *In Vivo*

Given that soluble Gpc4 induces release of NP1 from neurons *in vitro*, we next asked whether the absence of Gpc4 would affect NP1 sub-cellular localization *in vivo*. We predicted that if Gpc4 is necessary to induce release of NP1 from axons, then there would be increased levels of NP1 within presynaptic terminals in Gpc4 KO mice due to a failure of release. To address this question, we performed immunohistochemistry against NP1 in brain sections of the SC from Gpc4 KO and WT littermates at P6, along with VGlut2 to mark RGC presynaptic terminals that form synapses in this region (Figures 6A–6D; Figure S5A). In Gpc4 KO mice, there was a 1.48-fold ± 0.14-fold increase in NP1 puncta number (Figure 6C) and increased overlap between NP1 and VGlut2 by 1.51-fold ± 0.03-fold compared to WT (Figure 6D). This suggests that in the absence of Gpc4, less NP1 is secreted and is instead retained in the presynaptic terminal. There was no difference in the number of VGlut2 puncta (0.92-fold ± 0.05-fold; Figure 6B), demonstrating that the absence of Gpc4 does not prevent axons from reaching their target or inhibit the formation of presynaptic specializations. The same results were obtained using a second antibody against NP1 (Figures S5C–S5F), and no signal was detected when the primary antibody was omitted (Figure S5G). We then asked whether this effect was specific to RGC projections or present in other parts of the visual system by examining colocalization of NP1 with VGlut2-positive projections from the dLGN to layer 4 of the VC at P6 (Figures 6E–6H; Figure S5B). As in the SC, in the Gpc4 KO VC, there was a significant increase in NP1 puncta number (1.88-fold ± 0.16-fold; Figure 6G) and in the number of colocalized NP1-VGlut2 puncta (2.3-fold ± 0.3-fold; Figure 6H), with no change in VGlut2 (0.95-fold ± 0.06-fold; Figure 6F).

To determine whether astrocytes are the major source of Gpc4 responsible for regulating release of NP1 in the visual system at P6, we crossed Gpc4 flox mice to an astrocyte-specific cre line to remove Gpc4 specifically from astrocytes. We screened three different astrocyte-specific cre lines for expression at P3 by crossing them to a tdTomato reporter: GFAPcre77.6, GFAPcre73.12, and Aldh1l1cre (Garcia et al., 2004; Gregorian et al., 2009; Tien et al., 2012). Only the Aldh1l1cre showed extensive expression of tdTomato in the SC and VC at P3, so we continued experiments with this line (Figures S6A and S6B). As Aldh1l1cre can be expressed in some radial glia and give recombination in late-born neurons (Foo and Dougherty, 2013; Tien et al., 2012), we immunostained for the neuronal marker NeuN in the Aldh1l1cre;tdTomato brain. This showed little overlap between NeuN and tdTomato in the SC but frequent overlap in the VC (Figure S6C). Based on these regional differences in astrocyte timing and specificity of cre expression, we chose to use Gpc4f/y;Aldh1l1cre mice and analyze the SC at P6. We carried out triple fluorescent ISH for Gpc4, Aldh1l1 (astrocytes), and Tubb3 (neurons). In the Gpc4f/y, we observed overlapping expression of Gpc4 mRNA with Aldh1l1, with little overlap with Tubb3, whereas in the Gpc4f/y;Aldh1l1cre +ve, there was an absence of Gpc4 mRNA in Aldh1l1-positive cells (Figure 6I). As in the global Gpc4 KO, we carried out immunostaining for VGlut2 and NP1 in the P6 SC and quantified colocalization between them (Figures 6J–6M). There was a significant increase in NP1 puncta number in the Gpc4f/y;Aldh1l1cre +ve compared to the Gpc4f/y littermates (1.66-fold ± 0.05-fold; Figure 6L) and an increase in the overlap of NP1 with VGlut2 (1.91-fold ± 0.53-fold; Figure 6M), with no change in VGlut2 (0.98-fold ± 0.01-fold; Figure 6K). These results demonstrate that in the absence of astrocyte Gpc4, increased levels of NP1 are present within presynaptic terminals throughout the developing visual system.

RPTPδ KO Mice Show Increased Retention of NP1 in Presynaptic Terminals *In Vivo*

Our *in vitro* findings show that Gpc4 signaling through RPTPδ is both necessary and sufficient to induce release of NP1 from RGCs. If RPTPδ is the presynaptic receptor for Gpc4 *in vivo*,

Figure 5. Gpc4 Is Expressed by Astrocytes throughout the Developing Visual System

(A and B) *In situ* hybridization (ISH) in the developing visual system at P6 shows presence of astrocytes and expression of Gpc4 mRNA. (A) Left: ISH for astrocyte marker Aldh1l1 in the SC at P6. Right: negative control probe, showing absence of signal. (B) ISH for Gpc4 in the visual system at P6 in Gpc4 WT (left) and KO (right)—SC (top), dLGN (middle), and VC (bottom). Gpc4 is expressed in all regions, and there is no signal in the Gpc4 KO. In each set, left, DAPI to mark cell nuclei; middle, low-power image of mRNA probe; and right high-power image of region outlined by red box in low-power image, arrowheads mark positive cells. Note the different image size for VC versus SC and dLGN. Scale bars for (A) and (B), 50 μm.

(C–F) Triple fluorescent ISH for Gpc4 and cell-specific markers in the SC (C and D) and VC (E and F) at P6 shows colocalization of Gpc4 with Aldh1l1 and limited overlap with the neuronal marker Tubb3. (C) Gpc4 mRNA (red) colocalizes with Aldh1l1 mRNA (green) and rarely with Tubb3 (blue) in the superficial layers of the SC at P6 in WT (top), absence of Gpc4 signal in Gpc4 KO (bottom). (D) Quantification of (C), fraction of Gpc4+Aldh1l1-positive cells and Gpc4+Tubb3-positive cells. n = 3 mice. (E) Gpc4 (red) mainly colocalizes with Aldh1l1 (green) with limited overlap with Tubb3 (blue) in layer 4 of VC at P6 in WT (top); absence of Gpc4 signal in Gpc4 KO (bottom). In (C) and (E), left panels show merge plus DAPI (inset shows zoom in of boxed region), followed by merge minus DAPI, then single-channel panels as labeled: Gpc4, Aldh1l1, and Tubb3. Arrowheads mark example cells positive for Gpc4 and Aldh1l1. (F) Quantification of (E), fraction of Gpc4+Aldh1l1-positive cells and Gpc4+Tubb3-positive cells. n = 3 mice. Scale bars for (C) and (E), 50 μm, zoomed-in box scale bar, 5 μm. Graphs show mean ± SEM, statistical analysis by t test, p value on graph.

See also Figure S4.

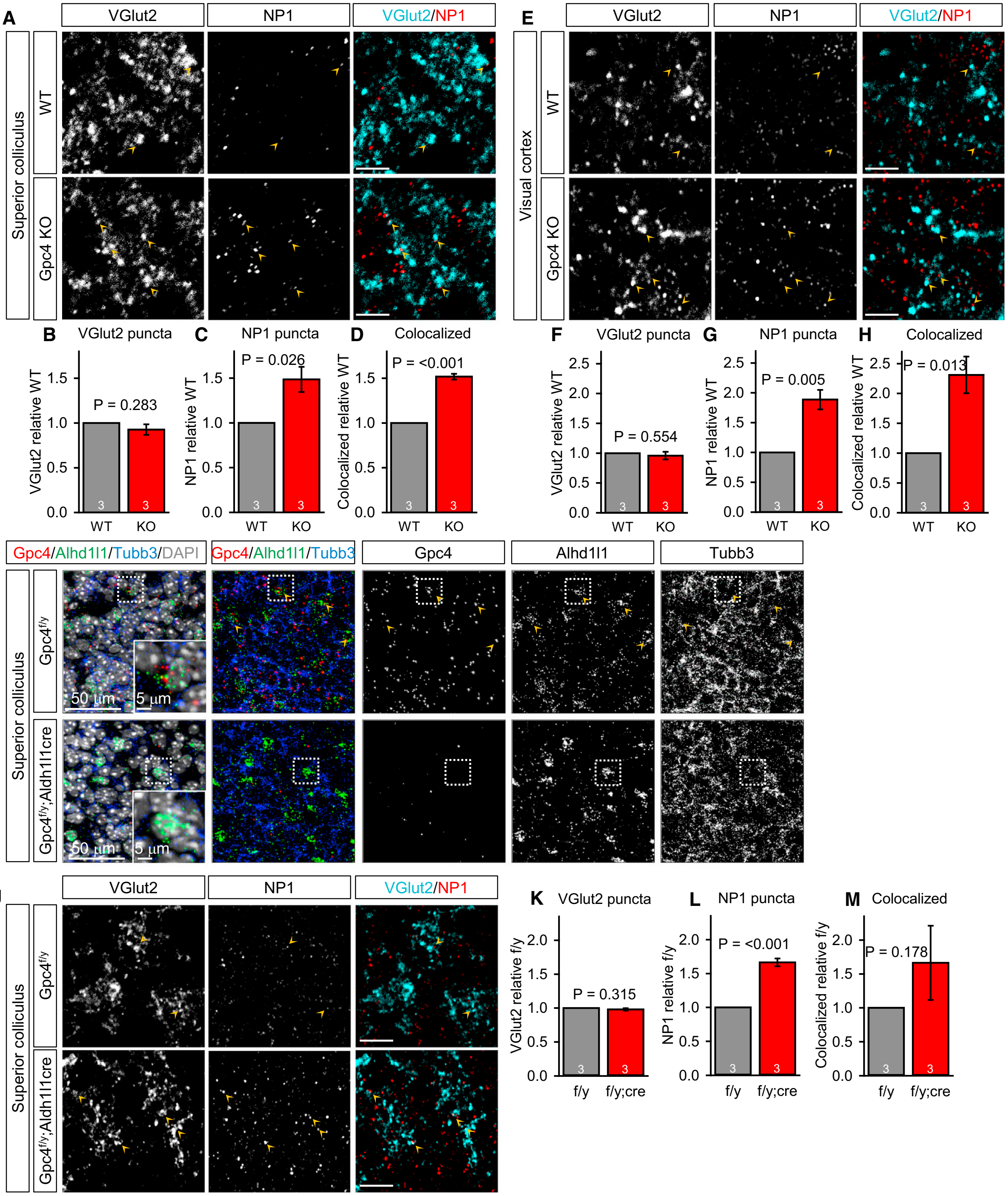

Figure 6. Gpc4 KO Mice Have Increased Overlap of NP1 with Presynaptic Terminals in the Developing Visual System *In Vivo*

(A–D) Increased colocalization between NP1 and VGlut2 in Gpc4 KO mice in the SC at P6 suggests reduced presynaptic release of NP1 in the absence of Gpc4. (A) Example images from the SC of WT (top) and Gpc4 KO (bottom), VGlut2 in cyan and NP1 in red. (B–D) Quantification of VGlut2, NP1, and colocalized puncta, respectively, normalized to WT.

(legend continued on next page)

then we predict that its removal will phenocopy Gpc4 KO mice, i.e., there will be an increase in the amount of NP1 present within the RPTPδ-lacking presynaptic terminals. To test this, we crossed RPTPδ flox mice to cre lines (see below) to remove RPTPδ from specific subsets of projection neurons in the visual system. In this way, neurons that project axons to form synapses in the target region (presynaptic) have reduced levels of RPTPδ, and neurons in the target zone (i.e., postsynaptic) still expressed RPTPδ, allowing us to differentiate between a pre- and postsynaptic necessity of RPTPδ to synaptic NP1 release. We first examined the *in vivo* localization of RPTPδ mRNA and protein in the developing visual system. RPTPδ mRNA is expressed in the dLGN and VC at P3 and P6 (Figure 7J, top; Figures S7G and S7H, top). We also examined protein expression of RPTPδ in the retina at P6 using a specific antibody (Figure S7K; Shishikura et al., 2016) and found that it is present in the retinal ganglion cell layer (GCL) and inner plexiform layer (IPL; Figure 7A). To determine whether RPTPδ is present in presynaptic terminals of RGC and dLGN neurons *in vivo*, we carried out immunostaining at P6 in the SC and VC, respectively, against VGlut2 and RPTPδ and observed punctate staining for RPTPδ that colocalized with VGlut2 (Figures 7B and 7K, top). Thus, RPTPδ is present in neurons of the visual system during development and is localized to presynaptic terminals, placing it in the right place at the right time to mediate Gpc4-induced release of NP1.

To delete RPTPδ from RGCs, we crossed RPTPδf/f mice to a Pax6-α-cre line (Marquardt et al., 2001). To visualize the recombination pattern, we crossed the Pax6-α-cre to an EGFP reporter line (Sapir et al., 2004) and observed robust GFP expression in multiple layers of the retina, including the GCL, IPL, and a subset of cells in the INL at P6 (Figure S7B). A reduction of RPTPδ in RPTPδ fl/fl;Pax6-α-cre +ve mice was confirmed by a decrease in RPTPδ protein in the retina shown by immunostaining and western blot, compared to cre –ve control (Figure 7A; Figure S7C). There was also a significant decrease in the overlap of RPTPδ puncta with VGlut2-positive presynaptic terminals in the SC at P6, showing that less receptor is present at the synapse (Figures 7B–7E). We next examined whether these terminals had increased colocalization between NP1 and VGlut2 (via immunostaining) (Figures 7F–7I). As in the Gpc4 KO, we observed an increase in NP1 puncta number (1.57-fold ± 0.08-fold; Figure 7H) and increased colocalization between VGlut2 and NP1 (1.56-fold ± 0.09-fold; Figure 7I) in the RPTPδ fl/fl;Pax6-α-cre +ve mice compared to cre –ve controls. There was no change in VGlut2 puncta number (0.95-fold ± 0.03-fold; Figure 7G), demonstrating that removal of RPTPδ did not alter the targeting of axons or the formation of presynaptic terminals.

To further examine the role of RPTPδ in Gpc4-regulated release of NP1, we removed RPTPδ from dLGN axons that project to the VC by crossing RPTPδf/f mice to an RORα cre line (Chou et al., 2013). To visualize the recombination pattern, we crossed RORα cre mice to a tdTomato reporter line (Figures S7D–S7F). Strong expression for tdTomato was observed in the dLGN at P6 (Figure S7D) and in axons in the VC, identified by overlapping staining with the thalamo-cortical presynaptic marker VGlut2 (Figure S7E) and lack of overlap with the intracortical presynaptic marker VGlut1 (Figure S7F). Reduction of RPTPδ in RPTPδ fl/fl;RORα cre +ve mice was confirmed by a decrease in RPTPδ mRNA in the thalamus compared to cre –ve controls (shown by ISH), with no alteration in mRNA level in the cortex (Figure 7J; Figures S7G–S7I). At the protein level, we observed a decrease in RPTPδ in tissue lysates from the thalamus of cre +ve mice compared to –ve by western blot, while the amount of RPTPδ in the hippocampus and VC remained unchanged (Figure S7J). Finally, there was a significant decrease in RPTPδ-VGlut2 colocalization in layer 4 of the VC at P6, showing that less receptor is present at the thalamo-cortical synapse (Figures 7K–7N). We then examined whether these terminals had increased colocalization between NP1 and VGlut2 (via immunostaining) (Figures 7O–7R). As above, we observed an increase in NP1 puncta number (1.7-fold ± 0.2-fold; Figure 7Q), an increase in NP1-VGlut2 colocalization (1.96-fold ± 0.5-fold; Figure 7R), and no change in VGlut2 puncta number (0.97-fold ± 0.05-fold; Figure 7P) in the RPTPδ fl/fl;RORα cre +ve mice compared to cre –ve controls. These results demonstrate that when RPTPδ is reduced in the presynaptic terminals, there is an increase in the amount of NP1 present within them, phenocopying the result seen in Gpc4 KO mice.

Gpc4 KO and RPTPδ KO Mice Have Decreased Recruitment of GluA1 to Synapses and Decreased Synapse Number in the Developing Superior Colliculus

Given that mice lacking Gpc4 and RPTPδ show increased presynaptic retention of NP1 *in vivo* (Figures 6 and 7), we next asked whether this pathway is necessary for recruitment of GluA1 to synapses or synapse formation in Gpc4 KO and RPTPδ fl/fl;Pax6-α-cre +ve mice. We carried out immunostaining for VGlut2 and GluA1 in the P6 SC of Gpc4 KO and WT littermates and assayed colocalization of these markers to determine synaptic recruitment of GluA1 (Figures 8A–8D). We observed a significant decrease in VGlut2-GluA1 colocalization

(E–H) Increased colocalization between NP1 and VGlut2 in Gpc4 KO mice in the VC at P6. (E) Example images from the VC of WT (top) and Gpc4 KO (bottom), VGlut2 in cyan and NP1 in red. (F–H) Quantification of VGlut2, NP1, and colocalized puncta, respectively, normalized to WT. In (A) and (E), arrowheads mark representative colocalized puncta. Scale bar, 5 μm.

(I) Triple fluorescent ISH for Gpc4- and cell-specific markers in the SC of astrocyte specific Gpc4 KO. Gpc4 (red) colocalizes with Aldh1l1 (green) and rarely with Tubb3 (blue) in the SC at P6 in Gpc4f/y;cre −ve (top), decrease of Gpc4 signal in Gpc4f/y;Aldh1l1cre +ve (bottom). Left panels merge plus DAPI (inset shows zoom in of boxed region), followed by merge minus DAPI, then single-channel panels as labeled: Gpc4, Aldh1l1, and Tubb3. Arrowheads mark example cells positive for Gpc4 and Aldh1l1. Scale bar, 50 μm, zoomed-in box scale bar, 5 μm.

(J–M) Increased colocalization between NP1 and VGlut2 in Gpc4f/y;Aldh1l1cre +ve mice in SC at P6, suggests reduced presynaptic release of NP1 in the absence of astrocyte Gpc4. (J) Example images from the SC of Gpc4f/y;cre −ve (top) and Gpc4f/y;cre +ve (bottom), VGlut2 in cyan and NP1 in red. Arrowheads mark representative colocalized puncta. Scale bar, 5 μm. (K–M) Quantification of VGlut2, NP1, and colocalized puncta, respectively, normalized to Gpc4f/y;cre −ve. Graphs show mean ± SEM, number of mice inside the bar. Statistical analysis by t test, p value on graph.

See also Figures S5 and S6.

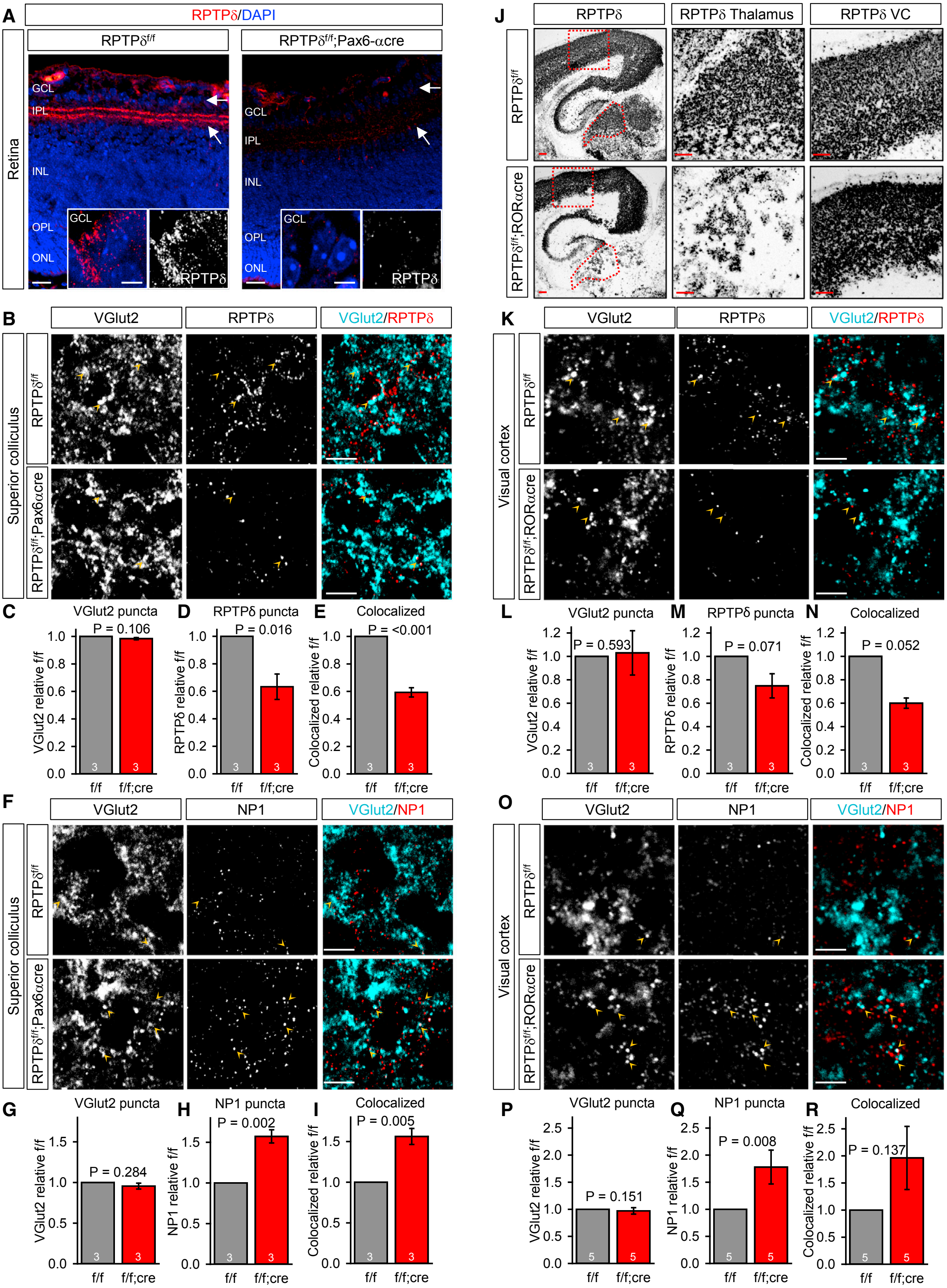

(legend on next page)

of 0.84-fold ± 0.03-fold (Figure 8D), with no change in VGlut2 (1.01-fold ± 0.07-fold; Figure 8B) and a decrease in total GluA1 (0.85-fold ± 0.06-fold; Figure 8C) in Gpc4 KO mice compared with WT. We then asked whether RPTPδ fl/fl;Pax6-α-cre +ve mice also have decreased synaptic recruitment of GluA1 by immunostaining for the same markers and comparing them to cre −ve littermates (Figures 8E–8H). There was a significant 0.75-fold ± 0.06-fold decrease in the colocalization of VGlut2 with GluA1 (Figure 8H), with no change in VGlut2 (1.04-fold ± 0.09-fold; Figure 8F) and a significant decrease in total GluA1 (0.78-fold ± 0.03-fold; Figure 8G) in RPTPδ fl/fl;Pax6-α-cre +ve mice. To ask whether structural synapse formation is also affected, we immunostained for VGlut2 and PSD95 in the P6 SC in the same mouse lines, and assayed colocalization of VGlut2 and PSD95 (Figures 8I–8P). In both the Gpc4 KO and RPTPδ fl/fl;Pax6-α-cre +ve mice, there was a significant decrease in the colocalization of PSD95 with VGlut2, compared to matched controls, of 0.67-fold ± 0.04-fold for Gpc4 KO (Figure 8L) and 0.83-fold ± 0.04-fold for RPTPδ fl/fl;Pax6-α-cre +ve (Figure 8P). There was no change in VGlut2 (0.96-fold ± 0.04-fold in Gpc4KO; 1.01-fold ± 0.03-fold in RPTPδ fl/fl;Pax6-α-cre +ve; Figures 8J and 8N) and a significant decrease in total PSD95 of 0.72-fold ± 0.03-fold in the Gpc4 KO (Figure 8K) and 0.75-fold ± 0.02-fold in the RPTPδ fl/fl;Pax6-α-cre +ve (Figure 8O). Taken together, these results demonstrate that astrocyte-secreted Gpc4 acting through presynaptic RPTPδ regulates secretion of NP1 from presynaptic terminals and controls synaptic recruitment of GluA1 and synapse formation in the developing SC.

DISCUSSION

How do neurons know when and where to initiate synapse formation? Previous work showed a correlation between the timing of synaptogenesis and the appearance of astrocytes in the developing brain (Ullian et al., 2001). We now identify a molecular mechanism for how this occurs: astrocytes signal to presynaptic axon terminals to increase the release of the AMPAR clustering factor NP1, which interacts with AMPARs on nearby dendrites and initiates nascent synapse formation (Figure S8). We demonstrate that: (1) Gpc4 signals through RPTPs in the presynaptic terminal; (2) Gpc4 upregulates release of the AMPAR-clustering factor NP1 from neurons; (3) binding of NP1 to postsynaptic GluA1 is necessary for Gpc4 to induce AMPAR clustering and synapse formation; and (4) mice lacking either Gpc4 or RPTPδ show increased retention of NP1 in presynaptic terminals, decreased synaptic GluA1, and decreased synapse number *in vivo*.

Astrocytes regulate AMPAR clustering and synapse formation in an indirect fashion by signaling through presynaptic receptors. One advantage of this pathway is that AMPARs will only be clustered once the axon and the target dendrite are both mature enough to undergo synaptogenesis. In this way, astrocytes provide spatial and temporal cues to regulate synapse formation by determining when and where axons release NP1. Neuronal pentraxins are clearly important for clustering AMPARs in development and plasticity (Sia et al., 2007), but the factors regulating NP1 release had not been previously characterized. Here we show that astrocytes, via the release of Gpc4, induce the release of NP1 from presynaptic terminals. Importantly, this effect is specific to the Gpc4 pathway, as the astrocyte-secreted protein TSP1, which induces formation of AMPAR-lacking silent synapses, does not affect NP1 release, highlighting the complexity of the role astrocytes play in synaptogenesis. In future experiments, it will be interesting to determine whether neuronal expressed Gpc4, present in the presynaptic terminals of subsets of neurons, is also able to regulate release of NP1.

Presynaptic RPTPs have been shown to regulate synaptogenesis via *trans*-synaptic binding to postsynaptic receptors, including Slitrks, NGL-3, TrkC, and Il1RAcP; however, little is known about the cellular pathways downstream of receptor-effector interaction (Coles et al., 2014; Takahashi and Craig, 2013). Interestingly, in some cases, the RPTP GAG-binding sites overlap with those used for *trans*-synaptic interactions (Coles et al., 2014; Yamagata et al., 2015). Heparan sulfate and heparin oligomers compete with TrkC for RPTPσ binding *in vitro* and

Figure 7. RPTPδ cKO Mice Have Increased Overlap of NP1 with Presynaptic Terminals in the Developing Visual System *In Vivo*

(A) RPTPδ protein is present in the retina at P6 and is reduced in RPTPδ fl/fl;Pax6-α-cre line (right). RPTPδ immunostaining red, DAPI blue. Arrows mark Ganglion cell layer (GCL) and inner plexiform layer (IPL), showing positive signal for RPTPδ. Scale bar, 20 μm. Boxes show zoomed-in image of GCL neurons; left, RPTPδ and DAPI channels; right, single-channel RPTPδ signal. Scale bar, 5 μm.

(B–E) Decreased colocalization between RPTPδ and VGlut2 in RPTPδ f/f;Pax6-α-cre mice in the SC at P6, demonstrating a decrease in the amount of RPTPδ receptor in RGC-SC synapse. (B) Example images from the SC of f/f (top) and f/f;Pax6-α-cre (bottom), VGlut2 in cyan, RPTPδ in red. (C–E) Quantification of VGlut2, RPTPδ, and colocalized puncta, respectively, normalized to f/f.

(F–I) Increased colocalization between NP1 and VGlut2 in retina-specific RPTPδ KO mice in SC at P6, suggesting reduced release of NP1 in the absence of presynaptic RPTPδ. (F) Example images from the SC of f/f (top) and f/f;Pax6-α-cre (bottom), VGlut2 in cyan and NP1 in red. (G–I) Quantification of VGlut2, NP1, and colocalized puncta, respectively, normalized to f/f. For (B) and (F), arrowheads mark representative colocalized puncta. Scale bar, 5 μm.

(J) RPTPδ mRNA is expressed throughout the developing visual system, and mRNA levels are reduced in the thalamus in RPTPδ f/f;RORα cre line. ISH for RPTPδ mRNA at P3, in f/f (top) and f/f;RORα cre +ve (bottom). Left: low-power images of thalamus and cortex, red box marks VC and red outline marks dLGN, scale bar, 100 μm. Middle is zoom in of dLGN, and right is zoom in of cortex. Scale bar, 100 μm.

(K–N) Decreased colocalization between RPTPδ protein and VGlut2 in RPTPδ f/f;RORα-cre +ve VC at P6, demonstrating decreased amount of RPTPδ in dLGN-VC synapse. (K) Example images from the VC of f/f (top) and f/f;RORαcre (bottom), VGlut2 in cyan, RPTPδ in red. (L–N) Quantification of VGlut2, RPTPδ, and colocalized puncta, respectively, normalized to f/f.

(O–R) Increased colocalization between NP1 and VGlut2 in thalamus-specific RPTPδ KO mice in the VC at P6. (O) Example images from the VC of f/f (top) and f/f;RORαcre (bottom), VGlut2 in cyan and NP1 in red. (P–R) Quantification of VGlut2, NP1, and colocalized puncta, respectively, normalized to f/f. In (K) and (O), arrowheads mark representative colocalized puncta. Scale bar, 5 μm. Graphs show mean ± SEM, number of mice inside the bar. Statistical analysis by t test, p value on graph.

See also Figure S7.

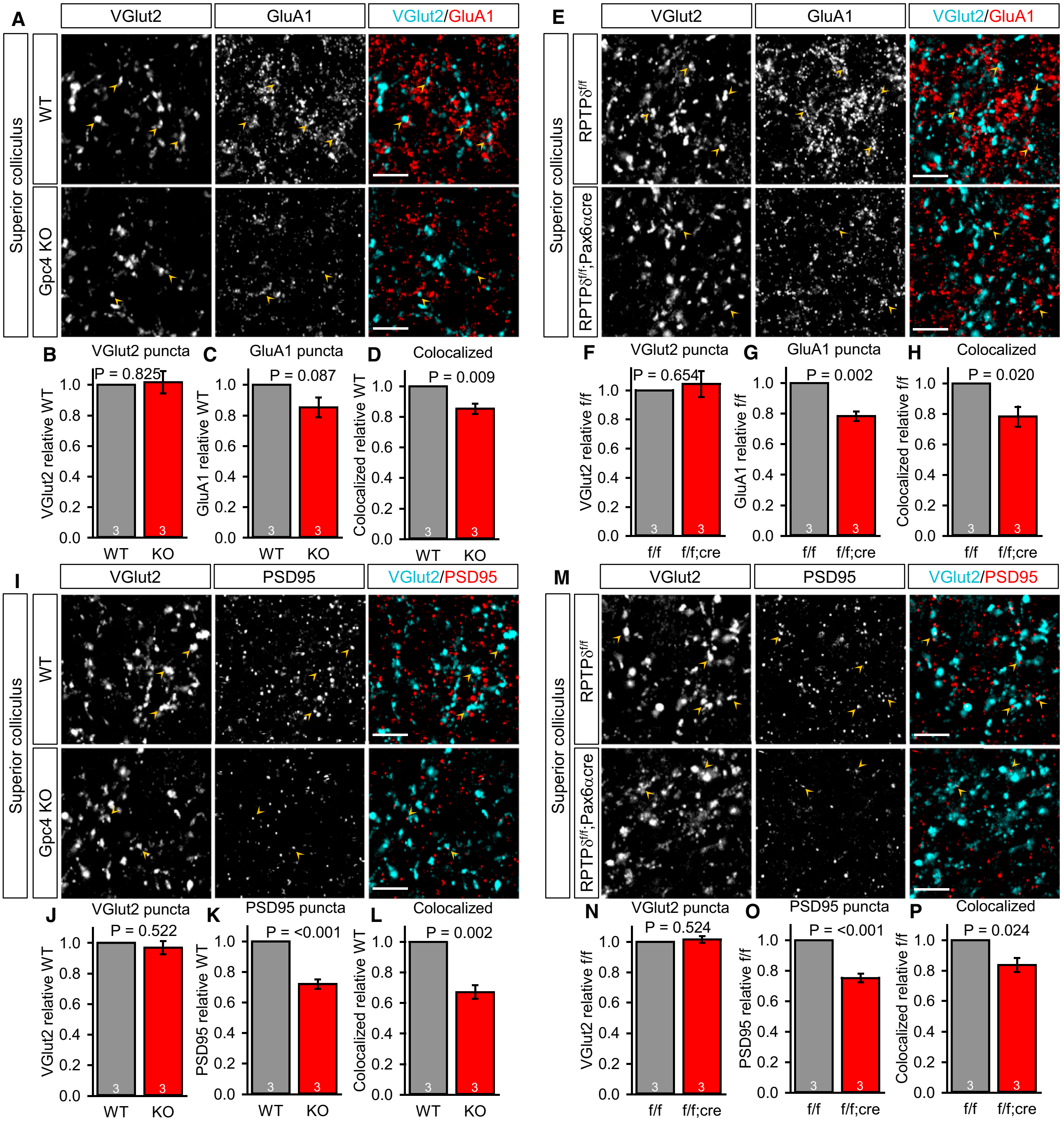

Figure 8. Gpc4 KO and RPTPδ cKO Mice Show Decreased Colocalization of GluA1 and PSD95 with VGlut2-Positive Presynaptic Terminals in the Developing Superior Colliculus

(A–D) Decreased colocalization between clusters of GluA1 and VGlut2 in Gpc4 KO mice in the SC at P6 shows reduced recruitment of GluA1 to the synapse in the absence of Gpc4. (A) Example images from the SC of WT (top) and Gpc4 KO (bottom), VGlut2 in cyan and GluA1 in red. (B–D) Quantification of VGlut2, GluA1, and colocalized puncta, respectively, normalized to WT.

(E–H) Decreased colocalization between clusters of GluA1 and VGlut2 in retina-specific RPTPδ KO (RPTPδ f/f;Pax6-α-cre) mice in the SC at P6 shows reduced recruitment of GluA1 to the synapse in the absence of presynaptic RPTPδ. (E) Example images from the SC of f/f (top) and f/f;Pax6-α-cre (bottom), VGlut2 in cyan and GluA1 in red. (F–H) Quantification of VGlut2, GluA1, and colocalized puncta, respectively, normalized to f/f.

(I–L) Decreased colocalization between PSD95 and VGlut2 in Gpc4 KO mice in the SC at P6 shows reduced formation of structural synapses in the absence of Gpc4. (I) Example images from the SC of WT (top) and Gpc4 KO (bottom), VGlut2 in cyan and PSD95 in red. (J–L) Quantification of VGlut2, PSD95, and colocalized puncta, respectively, normalized to WT.

(legend continued on next page)

disrupt TrkC-dependent synaptic differentiation in neuronal co-culture assays (Coles et al., 2014). Binding of Gpc4, an HSPG, to RPTPσ and RPTPδ may also trigger remodeling of such *trans*-synaptic complexes, which likely impacts downstream signaling through RPTPs. We now identify that interaction of soluble Gpc4 with RPTPδ regulates a pathway in the presynaptic terminal that leads to the release of NP1. How interaction between Gpc4 and RPTPδ induces the release of NP1 is still an open question. RPTPs are known to interact with syd-2/liprin-α via their intracellular D2 domain, which affects presynaptic differentiation, so one possibility is that this also regulates NP1 release (Kaufmann et al., 2002; Zhen and Jin, 1999). We found that both RPTPδ and RPTPσ are necessary for Gpc4 to induce synaptogenesis, but only RPTPδ was specific to the Gpc4 pathway, as RPTPσ also regulated TSP1-induced synapse formation. One interesting hypothesis is that these two receptors are working together to regulate synaptogenesis. RPTPδ has been reported to regulate the function of RPTPσ via interactions between their D2 domains, so one explanation is that Gpc4 interacting with RPTPδ is upstream of effects through RPTPσ (Wallace et al., 1998). Moreover, it has been shown that knocking out both RPTPσ and RPTPδ leads to early lethality, while knockout of individual receptors does not, suggesting that together these receptors are essential for neuronal development (Uetani et al., 2006).

What are the functional implications of understanding how astrocyte-secreted Gpc4 induces excitatory synapse formation? A strong correlation has been found between alterations in genes that encode synaptic proteins and neurological disorders, suggesting synaptic dysfunction as an underlying mechanism in disorders such as ASD (Parikshak et al., 2013). Alterations in astrocyte function have also been shown in a number of neurodevelopmental disorders, including ASD and schizophrenia (Allen, 2013). Moreover, alterations in glypicans (Calboli et al., 2010; Doan et al., 2016; Lesch et al., 2008; Pinto et al., 2010; Potkin et al., 2010) and RPTPδ (Choucair et al., 2015; Elia et al., 2010; Pinto et al., 2010) have been linked to the pathophysiology of disorders, including ASD, schizophrenia, ADHD, and neuroticism, and more recently, neuronal pentraxins have been linked to cognitive dysfunction in Alzheimer's disease (Xiao et al., 2017). A recent study identified genomic regions known as human accelerated regions (HARs) that are hypothesized to contribute to human evolution and cognition. Interestingly, regions within both Gpc4 and RPTPδ have been identified as potential HARs (Doan et al., 2016), and two cases of ASD with intellectual disability (ID) were found that have point mutations in HARs of Gpc4 causing a significant decrease in Gpc4 expression (Doan et al., 2016). These results strongly suggest that the levels of Gpc4 expressed in the human brain are crucial for brain development and synaptogenesis, with too little Gpc4 leading to ASD and ID. To conclude, we have identified the molecular pathway that astrocyte-secreted Gpc4 acts through to regulate excitatory synaptogenesis, providing novel targets to manipulate to alter synaptic function in health and disease.

STAR★METHODS

Detailed methods are provided in the online version of this paper and include the following:

- KEY RESOURCES TABLE
- CONTACT FOR REAGENT AND RESOURCE SHARING
- EXPERIMENTAL MODELS
  - Animals
  - Cell Culture
- METHOD DETAILS
  - cDNA Constructs
  - Recombinant Proteins
  - GluA1-Fab Generation
  - GluA1-Fab Fragment Specificity Analysis
  - Preparation of Astrocyte Conditioned Media (ACM)
  - Western Blot
  - RNA Analysis from RGCs *In Vitro*
  - Immunostaining RGCs *In Vitro*
  - Mouse Brain Tissue Preparation and Immunostaining
- QUANTIFICATION AND STATISTICAL ANALYSIS
- DATA AND SOFTWARE AVAILABILITY

SUPPLEMENTAL INFORMATION

Supplemental Information includes eight figures and three tables and can be found with this article online at https://doi.org/10.1016/j.neuron.2017.09.053.

AUTHOR CONTRIBUTIONS

I.F.-T. and N.J.A. conceived the project, designed the experiments, and wrote the manuscript with input from authors. I.F.-T. performed all experiments except as noted. A.C.M.v.C. performed qRT-PCR and contributed to Gpc4-NP1 time course immunostaining experiments. A.L., V.T.C., and A.R.A. designed the Fab and recombinant NP1 reagents. These were produced and validated by A.L. and V.T.C. in surface plasmon resonance and cell-binding experiments.

ACKNOWLEDGMENTS

We thank Cari Dowling, Angeline Ngo, Alex Kopelevich, and Benjamin Bishop for technical assistance. We thank members of the Allen lab and the Molecular Neurobiology Laboratory for helpful discussions. We thank F. Nakamura for the RPTPδ antibody, D. O'Leary for the RORαcre and Pax6αcre lines, and C. Coles and N. Mitakidis for full-length RPTPσ, RPTPδ, LAR constructs. We thank the Biophotonics Core at the Salk Institute for advice with image acquisition and analysis. Work in the lab of N.J.A. is supported by NIH-NINDS R01 NS089791, the Hearst, Pew, Ellison, Dana, and Whitehall Foundations and Larry and Carol Greenfield. I.F.-T. was supported by a Postdoctoral Fellowship from the Catarina Foundation and A.L. by a Wellcome Trust DPhil studentship. V.T.C. and A.R.A. were funded by the UK Medical Research Council (MRC) (grants L009609 and MC_UP_1201/15 to A.R.A.). This work was supported by Core Facilities of the Salk Institute: (Peptide Synthesis, Functional Genomics, Transgenic: NIH-NCI CCSG P30 014195), (Biophotonics: NIH-NINDS

(M–P) Decreased colocalization between PSD95 and VGlut2 in retina-specific RPTPδ KO (RPTPδ f/f;Pax6-α-cre) mice in the SC at P6 shows reduced formation of structural synapses in the absence of presynaptic RPTPδ. (M) Example images from the SC of f/f (top) and f/f;Pax6-α-cre (bottom), VGlut2 in cyan and PSD95 in red. (N–P) Quantification of VGlut2, PSD95, and colocalized puncta, respectively, normalized to f/f. Arrowheads mark representative colocalized puncta. Scale bars, 5 μm. Graphs show mean ± SEM, number of mice inside the bar. Statistical analysis by t test, p value on graph.

P30 NS072031, NIH-NCI CCSG P30 014195, Waitt Foundation) and by the Wellcome Trust Centre for Human Genetics (grant 090532/Z/09/Z).

Received: April 28, 2017
Revised: August 31, 2017
Accepted: September 27, 2017
Published: October 11, 2017

REFERENCES

Allen, N.J. (2013). Role of glia in developmental synapse formation. Curr. Opin. Neurobiol. *23*, 1027–1033.

Allen, N.J., Bennett, M.L., Foo, L.C., Wang, G.X., Chakraborty, C., Smith, S.J., and Barres, B.A. (2012). Astrocyte glypicans 4 and 6 promote formation of excitatory synapses via GluA1 AMPA receptors. Nature *486*, 410–414.

Aricescu, A.R., Lu, W., and Jones, E.Y. (2006). A time- and cost-efficient system for high-level protein production in mammalian cells. Acta Crystallogr. D Biol. Crystallogr. *62*, 1243–1250.

Bassani, S., Folci, A., Zapata, J., and Passafaro, M. (2013). AMPAR trafficking in synapse maturation and plasticity. Cell. Mol. Life Sci. *70*, 4411–4430.

Bjartmar, L., Huberman, A.D., Ullian, E.M., Rentería, R.C., Liu, X., Xu, W., Prezioso, J., Susman, M.W., Stellwagen, D., Stokes, C.C., et al. (2006). Neuronal pentraxins mediate synaptic refinement in the developing visual system. J. Neurosci. *26*, 6269–6281.

Brooks, J.M., Su, J., Levy, C., Wang, J.S., Seabrook, T.A., Guido, W., and Fox, M.A. (2013). A molecular mechanism regulating the timing of corticogeniculate innervation. Cell Rep. *5*, 573–581.

Cahoy, J.D., Emery, B., Kaushal, A., Foo, L.C., Zamanian, J.L., Christopherson, K.S., Xing, Y., Lubischer, J.L., Krieg, P.A., Krupenko, S.A., et al. (2008). A transcriptome database for astrocytes, neurons, and oligodendrocytes: a new resource for understanding brain development and function. J. Neurosci. *28*, 264–278.

Calboli, F.C., Tozzi, F., Galwey, N.W., Antoniades, A., Mooser, V., Preisig, M., Vollenweider, P., Waterworth, D., Waeber, G., Johnson, M.R., et al. (2010). A genome-wide association study of neuroticism in a population-based sample. PLoS ONE *5*, e11504.

Chou, S.J., Babot, Z., Leingärtner, A., Studer, M., Nakagawa, Y., and O'Leary, D.D. (2013). Geniculocortical input drives genetic distinctions between primary and higher-order visual areas. Science *340*, 1239–1242.

Choucair, N., Mignon-Ravix, C., Cacciagli, P., Abou Ghoch, J., Fawaz, A., Mégarbané, A., Villard, L., and Chouery, E. (2015). Evidence that homozygous PTPRD gene microdeletion causes trigonocephaly, hearing loss, and intellectual disability. Mol. Cytogenet. *8*, 39.

Christopherson, K.S., Ullian, E.M., Stokes, C.C., Mullowney, C.E., Hell, J.W., Agah, A., Lawler, J., Mosher, D.F., Bornstein, P., and Barres, B.A. (2005). Thrombospondins are astrocyte-secreted proteins that promote CNS synaptogenesis. Cell *120*, 421–433.

Coles, C.H., Shen, Y., Tenney, A.P., Siebold, C., Sutton, G.C., Lu, W., Gallagher, J.T., Jones, E.Y., Flanagan, J.G., and Aricescu, A.R. (2011). Proteoglycan-specific molecular switch for RPTPσ clustering and neuronal extension. Science *332*, 484–488.

Coles, C.H., Mitakidis, N., Zhang, P., Elegheert, J., Lu, W., Stoker, A.W., Nakagawa, T., Craig, A.M., Jones, E.Y., and Aricescu, A.R. (2014). Structural basis for extracellular cis and transRPTPσ signal competition in synaptogenesis. Nat. Commun. *5*, 5209.

de Wit, J., O'Sullivan, M.L., Savas, J.N., Condomitti, G., Caccese, M.C., Vennekens, K.M., Yates, J.R., 3rd, and Ghosh, A. (2013). Unbiased discovery of glypican as a receptor for LRRTM4 in regulating excitatory synapse development. Neuron *79*, 696–711.

Doan, R.N., Bae, B.-I., Cubelos, B., Chang, C., Hossain, A.A., Al-Saad, S., Mukaddes, N.M., Oner, O., Al-Saffar, M., Balkhy, S., et al.; Homozygosity Mapping Consortium for Autism (2016). Mutations in human accelerated regions disrupt cognition and Ssocial behavior. Cell *167*, 341–354.e12.

Dunah, A.W., Hueske, E., Wyszynski, M., Hoogenraad, C.C., Jaworski, J., Pak, D.T., Simonetta, A., Liu, G., and Sheng, M. (2005). LAR receptor protein tyrosine phosphatases in the development and maintenance of excitatory synapses. Nat. Neurosci. *8*, 458–467.

Elia, J., Gai, X., Xie, H.M., Perin, J.C., Geiger, E., Glessner, J.T., D'arcy, M., deBerardinis, R., Frackelton, E., Kim, C., et al. (2010). Rare structural variants found in attention-deficit hyperactivity disorder are preferentially associated with neurodevelopmental genes. Mol. Psychiatry *15*, 637–646.

Eroglu, C., Allen, N.J., Susman, M.W., O'Rourke, N.A., Park, C.Y., Özkan, E., Chakraborty, C., Mulinyawe, S.B., Annis, D.S., Huberman, A.D., et al. (2009). Gabapentin receptor α2δ-1 is a neuronal thrombospondin receptor responsible for excitatory CNS synaptogenesis. Cell *139*, 380–392.

Foo, L.C., and Dougherty, J.D. (2013). Aldh1L1 is expressed by postnatal neural stem cells in vivo. Glia *61*, 1533–1541.

Garcia, A.D.R., Doan, N.B., Imura, T., Bush, T.G., and Sofroniew, M.V. (2004). GFAP-expressing progenitors are the principal source of constitutive neurogenesis in adult mouse forebrain. Nat. Neurosci. *7*, 1233–1241.

Gregorian, C., Nakashima, J., Le Belle, J., Ohab, J., Kim, R., Liu, A., Smith, K.B., Groszer, M., Garcia, A.D., Sofroniew, M.V., et al. (2009). Pten deletion in adult neural stem/progenitor cells enhances constitutive neurogenesis. J. Neurosci. *29*, 1874–1886.

Groc, L., Gustafsson, B., and Hanse, E. (2006). AMPA signalling in nascent glutamatergic synapses: there and not there! Trends Neurosci. *29*, 132–139.

Henley, J.M., and Wilkinson, K.A. (2016). Synaptic AMPA receptor composition in development, plasticity and disease. Nat. Rev. Neurosci. *17*, 337–350.

Horn, K.E., Xu, B., Gobert, D., Hamam, B.N., Thompson, K.M., Wu, C.-L., Bouchard, J.-F., Uetani, N., Racine, R.J., Tremblay, M.L., et al. (2012). Receptor protein tyrosine phosphatase sigma regulates synapse structure, function and plasticity. J. Neurochem. *122*, 147–161.

Howarth, M., and Ting, A.Y. (2008). Imaging proteins in live mammalian cells with biotin ligase and monovalent streptavidin. Nat. Protoc. *3*, 534–545.

Johnson, K.G., Tenney, A.P., Ghose, A., Duckworth, A.M., Higashi, M.E., Parfitt, K., Marcu, O., Heslip, T.R., Marsh, J.L., Schwarz, T.L., et al. (2006). The HSPGs Syndecan and Dallylike bind the receptor phosphatase LAR and exert distinct effects on synaptic development. Neuron *49*, 517–531.

Kaufmann, N., DeProto, J., Ranjan, R., Wan, H., and Van Vactor, D. (2002). Drosophila liprin-alpha and the receptor phosphatase Dlar control synapse morphogenesis. Neuron *34*, 27–38.

Kettleborough, C.A., Saldanha, J., Ansell, K.H., and Bendig, M.M. (1993). Optimization of primers for cloning libraries of mouse immunoglobulin genes using the polymerase chain reaction. Eur. J. Immunol. *23*, 206–211.

Ko, J.S., Pramanik, G., Um, J.W., Shim, J.S., Lee, D., Kim, K.H., Chung, G.-Y., Condomitti, G., Kim, H.M., Kim, H., et al. (2015). PTPσ functions as a presynaptic receptor for the glypican-4/LRRTM4 complex and is essential for excitatory synaptic transmission. Proc. Natl. Acad. Sci. USA *112*, 1874–1879.

Koch, S.M., and Ullian, E.M. (2010). Neuronal pentraxins mediate silent synapse conversion in the developing visual system. J. Neurosci. *30*, 5404–5414.

Kucukdereli, H., Allen, N.J., Lee, A.T., Feng, A., Ozlu, M.I., Conatser, L.M., Chakraborty, C., Workman, G., Weaver, M., Sage, E.H., et al. (2011). Control of excitatory CNS synaptogenesis by astrocyte-secreted proteins Hevin and SPARC. Proc. Natl. Acad. Sci. USA *108*, E440–E449.

Lai, M., Hughes, E.G., Peng, X., Zhou, L., Gleichman, A.J., Shu, H., Matà, S., Kremens, D., Vitaliani, R., Geschwind, M.D., et al. (2009). AMPA receptor antibodies in limbic encephalitis alter synaptic receptor location. Ann. Neurol. *65*, 424–434.

Lang, B.T., Cregg, J.M., DePaul, M.A., Tran, A.P., Xu, K., Dyck, S.M., Madalena, K.M., Brown, B.P., Weng, Y.-L., Li, S., et al. (2015). Modulation of the proteoglycan receptor PTPσ promotes recovery after spinal cord injury. Nature *518*, 404–408.

Lee, S.J., Wei, M., Zhang, C., Maxeiner, S., Pak, C., Calado Botelho, S., Trotter, J., Sterky, F.H., and Südhof, T.C. (2017). Presynaptic neuronal pentraxin receptor organizes excitatory and inhibitory synapses. J. Neurosci. *37*, 1062–1080.

Lesch, K.P., Timmesfeld, N., Renner, T.J., Halperin, R., Röser, C., Nguyen, T.T., Craig, D.W., Romanos, J., Heine, M., Meyer, J., et al. (2008). Molecular genetics of adult ADHD: converging evidence from genome-wide association and extended pedigree linkage studies. J. Neural Transm. (Vienna) *115*, 1573–1585.

Marquardt, T., Ashery-Padan, R., Andrejewski, N., Scardigli, R., Guillemot, F., and Gruss, P. (2001). Pax6 is required for the multipotent state of retinal progenitor cells. Cell *105*, 43–55.

McCarthy, K.D., and de Vellis, J. (1980). Preparation of separate astroglial and oligodendroglial cell cultures from rat cerebral tissue. J. Cell Biol. *85*, 890–902.

Meathrel, K., Adamek, T., Batt, J., Rotin, D., and Doering, L.C. (2002). Protein tyrosine phosphatase σ-deficient mice show aberrant cytoarchitecture and structural abnormalities in the central nervous system. J. Neurosci. Res. *70*, 24–35.

Myszka, D.G. (1999). Improving biosensor analysis. J. Mol. Recognit. *12*, 279–284.

O'Brien, R.J., Xu, D., Petralia, R.S., Steward, O., Huganir, R.L., and Worley, P. (1999). Synaptic clustering of AMPA receptors by the extracellular immediate-early gene product Narp. Neuron *23*, 309–323.

Parikshak, N.N., Luo, R., Zhang, A., Won, H., Lowe, J.K., Chandran, V., Horvath, S., and Geschwind, D.H. (2013). Integrative functional genomic analyses implicate specific molecular pathways and circuits in autism. Cell *155*, 1008–1021.

Pinto, D., Pagnamenta, A.T., Klei, L., Anney, R., Merico, D., Regan, R., Conroy, J., Magalhaes, T.R., Correia, C., Abrahams, B.S., et al. (2010). Functional impact of global rare copy number variation in autism spectrum disorders. Nature *466*, 368–372.

Potkin, S.G., Macciardi, F., Guffanti, G., Fallon, J.H., Wang, Q., Turner, J.A., Lakatos, A., Miles, M.F., Lander, A., Vawter, M.P., and Xie, X. (2010). Identifying gene regulatory networks in schizophrenia. Neuroimage *53*, 839–847.

Sapir, T., Geiman, E.J., Wang, Z., Velasquez, T., Mitsui, S., Yoshihara, Y., Frank, E., Alvarez, F.J., and Goulding, M. (2004). Pax6 and engrailed 1 regulate two distinct aspects of renshaw cell development. J. Neurosci. *24*, 1255–1264.

Shishikura, M., Nakamura, F., Yamashita, N., Uetani, N., Iwakura, Y., and Goshima, Y. (2016). Expression of receptor protein tyrosine phosphatase δ, PTPδ, in mouse central nervous system. Brain Res. *1642*, 244–254.

Sia, G.M., Béïque, J.C., Rumbaugh, G., Cho, R., Worley, P.F., and Huganir, R.L. (2007). Interaction of the N-terminal domain of the AMPA receptor GluR4 subunit with the neuronal pentraxin NP1 mediates GluR4 synaptic recruitment. Neuron *55*, 87–102.

Siddiqui, T.J., Tari, P.K., Connor, S.A., Zhang, P., Dobie, F.A., She, K., Kawabe, H., Wang, Y.T., Brose, N., and Craig, A.M. (2013). An LRRTM4-HSPG complex mediates excitatory synapse development on dentate gyrus granule cells. Neuron *79*, 680–695.

Singh, S.K., Stogsdill, J.A., Pulimood, N.S., Dingsdale, H., Kim, Y.H., Pilaz, L.J., Kim, I.H., Manhaes, A.C., Rodrigues, W.S., Jr., Pamukcu, A., et al. (2016). Astrocytes assemble thalamocortical synapses by bridging NRX1α and NL1 via Hevin. Cell *164*, 183–196.

Takahashi, H., and Craig, A.M. (2013). Protein tyrosine phosphatases PTPδ, PTPσ, and LAR: presynaptic hubs for synapse organization. Trends Neurosci. *36*, 522–534.

Takahashi, H., Arstikaitis, P., Prasad, T., Bartlett, T.E., Wang, Y.T., Murphy, T.H., and Craig, A.M. (2011). Postsynaptic TrkC and presynaptic PTPσ function as a bidirectional excitatory synaptic organizing complex. Neuron *69*, 287–303.

Tien, A.-C., Tsai, H.-H., Molofsky, A.V., McMahon, M., Foo, L.C., Kaul, A., Dougherty, J.D., Heintz, N., Gutmann, D.H., Barres, B.A., and Rowitch, D.H. (2012). Regulated temporal-spatial astrocyte precursor cell proliferation involves BRAF signalling in mammalian spinal cord. Development *139*, 2477–2487.

Uetani, N., Kato, K., Ogura, H., Mizuno, K., Kawano, K., Mikoshiba, K., Yakura, H., Asano, M., and Iwakura, Y. (2000). Impaired learning with enhanced hippocampal long-term potentiation in PTPdelta-deficient mice. EMBO J. *19*, 2775–2785.

Uetani, N., Chagnon, M.J., Kennedy, T.E., Iwakura, Y., and Tremblay, M.L. (2006). Mammalian motoneuron axon targeting requires receptor protein tyrosine phosphatases σ and δ. J. Neurosci. *26*, 5872–5880.

Ullian, E.M., Sapperstein, S.K., Christopherson, K.S., and Barres, B.A. (2001). Control of synapse number by glia. Science *291*, 657–661.

Wallace, M.J., Fladd, C., Batt, J., and Rotin, D. (1998). The second catalytic domain of protein tyrosine phosphatase δ (PTP δ) binds to and inhibits the first catalytic domain of PTP ς. Mol. Cell. Biol. *18*, 2608–2616.

Winzeler, A., and Wang, J.T. (2013). Purification and culture of retinal ganglion cells from rodents. Cold Spring Harb. Protoc. *2013*, 643–652.

Xiao, M.-F., Xu, D., Craig, M.T., Pelkey, K.A., Chien, C.-C., Shi, Y., Zhang, J., Resnick, S., Pletnikova, O., Salmon, D., et al. (2017). NPTX2 and cognitive dysfunction in Alzheimer's disease. eLife *6*, e23798.

Xie, Y., Massa, S.M., Ensslen-Craig, S.E., Major, D.L., Yang, T., Tisi, M.A., Derevyanny, V.D., Runge, W.O., Mehta, B.P., Moore, L.A., et al. (2006). Protein-tyrosine phosphatase (PTP) wedge domain peptides: a novel approach for inhibition of PTP function and augmentation of protein-tyrosine kinase function. J. Biol. Chem. *281*, 16482–16492.

Xu, D., Hopf, C., Reddy, R., Cho, R.W., Guo, L., Lanahan, A., Petralia, R.S., Wenthold, R.J., O'Brien, R.J., and Worley, P. (2003). Narp and NP1 form heterocomplexes that function in developmental and activity-dependent synaptic plasticity. Neuron *39*, 513–528.

Yamagata, A., Sato, Y., Goto-Ito, S., Uemura, T., Maeda, A., Shiroshima, T., Yoshida, T., and Fukai, S. (2015). Structure of Slitrk2-PTPδ complex reveals mechanisms for splicing-dependent trans-synaptic adhesion. Sci. Rep. *5*, 9686.

Zhen, M., and Jin, Y. (1999). The liprin protein SYD-2 regulates the differentiation of presynaptic termini in C. elegans. Nature *401*, 371–375.

STAR★METHODS

KEY RESOURCES TABLE

REAGENT or RESOURCE	SOURCE	IDENTIFIER
Antibodies		
Rat anti-HA tag	Roche	CAT# 11867423001; RRID: AB_390918
Rabbit anti-HA tag	Sigma	CAT# H6908; RRID: AB_260070
Rabbit anti-FLAG	Sigma	CAT# F7425; RRID: AB_439687
Rabbit polyclonal anti-Glypican 4	Proteintech	CAT# 13048-1-AP; RRID: AB_10640157
Rat monoclonal anti-Homer	Serotec	CAT# AHP736; RRID: AB_324009
Rabbit polyclonal anti-Homer	Santa Cruz	CAT# sc-20807; RRID: AB_2120997
Mouse monoclonal anti-PSD95	Thermo Fisher Scientific	CAT# MA1-045; RRID: AB_325399
Rabbit polyclonal anti-PSD95	Life Technologies	CAT# 51-6900; RRID: AB_2533914
Mouse monoclonal anti-Bassoon	Enzo	CAT# VAM-PS003; RRID: AB_2066982
Rabbit polyclonal anti-Bassoon	Synaptic Systems	CAT# 141 002; RRID :AB_887698
Guinea pig polyclonal anti-VGlut1	Millipore	CAT# AB5905; RRID: AB_2301751
Guinea pig polyclonal anti-VGlut2	Millipore	CAT# AB2251; RRID: AB_2665454
Mouse monoclonal anti-β-tubulin	Thermo Fisher Scientific	CAT# MA5-16308; RRID: AB_2537819
Mouse monoclonal anti-NP1	BD Trans. Lab	CAT# 610369; RRID: AB_397754
Rabbit polyclonal anti-NP1	Thermo Fisher Scientific	CAT# PA514136; RRID: AB_2236166
Rat monoclonal anti-RPTPδ	Dr. F. Nakamura	Shishikura et al., 2016
Mouse monoclonal anti-GluA1	Millipore	CAT# MAB2263; RRID: AB_1977459
Rabbit polyclonal anti-GluA1	Millipore	CAT# AB1504; RRID: AB_2113602
Mouse monoclonal anti-NeuN	Millipore	CAT# MAB377; RRID: AB_2298772
Chicken polyclonal anti-GFP	Millipore	CAT# 06-896; RRID: AB_11214044
Goat anti-Rat Alexa 488	Molecular Probes	CAT# A11006; RRID: AB_141373
Goat anti-Rat Alexa 594	Molecular Probes	CAT# A11007; RRID: AB_141374
Goat anti-Rat Alexa 680	Molecular Probes	CAT# A21096; RRID: AB_141554
Goat anti-Mouse Alexa 488	Molecular Probes	CAT# A11029; RRID: AB_138404
Goat anti-Mouse Alexa 555	Molecular Probes	CAT# A21424; RRID: AB_141780
Goat anti-Mouse Alexa 594	Molecular Probes	CAT# A11032; RRID: AB_141672
Goat anti-Mouse Alexa 680	Molecular Probes	CAT# A21057; RRID: AB_141436
Goat anti-Rabbit Alexa 488	Molecular Probes	CAT# A11034; RRID: AB_2576217
Goat anti-Rabbit Alexa 594	Molecular Probes	CAT# A11037; RRID: AB_2534095
Goat anti-Rabbit Alexa 647	Molecular Probes	CAT# A21245; RRID: AB_2535813
Goat anti-Rabbit Alexa 680	Molecular Probes	CAT# A21109; RRID: AB_2535758
Goat anti-Guinea pig Alexa 488	Molecular Probes	CAT# A11073; RRID: AB_142018
Goat anti-Guinea pig Alexa 594	Molecular Probes	CAT# A11076; RRID: AB_141930
Goat anti-Guinea pig Alexa 647	Molecular Probes	CAT# A21450; RRID: AB_141882
Goat anti-Chicken Alexa 488	Molecular Probes	CAT# A11039; RRID: AB_142924
Goat anti-Mouse (Fab specific)-FITC	Sigma	CAT# F4018; RRID: AB_259572
Chemicals, Peptides, and Recombinant Proteins		
His-tagged glypican 4	This study, Allen lab	Allen et al., 2012
Alexa 488-tagged NP1	This study, Aricescu lab	N/A
GluA1-Fab	This study, Aricescu lab	N/A
Mut-Fab	This study, Aricescu lab	N/A
Dulbeco's modified phosphate buffered saline (DPBS)	HyClone	CAT# SH30264
Papain	Worthington	CAT# PAP2 3176

(*Continued on next page*)

Continued

REAGENT or RESOURCE	SOURCE	IDENTIFIER
Trypsin inhibitor	Worthington	CAT# LS003086
Isolectin	Vector	CAT# L-1100
Trypsin	Sigma	CAT# T9935
Poly-D-Lysine	Sigma	CAT# P6407
Laminin	Cultrex Trevigen	CAT# 3400-010-01
DMEM	Thermo Fisher Scientific	CAT# 11960044
Neurobasal	Thermo Fisher Scientific	CAT# 21103049
Penicillin-Streptomycin	Thermo Fisher Scientific	CAT# 15140-122
Glutamax	Thermo Fisher Scientific	CAT# 35050-061
Sodium pyruvate	Thermo Fisher Scientific	CAT# 11360-070
FBS	Thermo Fisher Scientific	CAT# 10437028
N-acetyl-L-cysteine	Sigma	CAT# A8199
Insulin	Sigma	CAT# I1882
Triiodo-thyronine	Sigma	CAT# T6397
Transferrin	Sigma	CAT# T1147
BSA	Sigma	CAT# A4161
Progesterone	Sigma	CAT# P6149
Putrescine	Sigma	CAT# P5780
Sodium selenite	Sigma	CAT# S9133
Forskolin	Sigma	CAT# F6886
FUDR	Sigma	CAT# F0503
AraC	Sigma	CAT# C1768
Hydrocortisone	Sigma	CAT# H0888
B27/NS21	Winzeler and Wang, 2013	N/A
BDNF	Peprotech	CAT# 450-02
CNTF	Peprotech	CAT# 450-13
Complete Protease Inhibitor cocktail	Sigma	CAT# 04693132001
Mouse Thrombospondin 1	R&D Systems	CAT# NP_035710
RPTP wedge sigma peptide	Salk Peptide Core	N/A
RPTP wedge scrambled sigma peptide	Salk Peptide Core	N/A
RPTP wedge LAR peptide	Salk Peptide Core	N/A
RPTP wedge delta peptide	Salk Peptide Core	N/A
Reducing sample buffer	Thermo Fisher Scientific	CAT# 39000
1% caesin	Biorad	CAT# 1610782
TBS	Bio-world	CAT# 105300272
Actinomycin D	Thermo Fisher Scientific	CAT# 11805017
Cell tracker red CMPTX dye	Thermo Fisher Scientific	CAT# C34552
16% PFA solution	EMS	CAT# 50980487
PFA powder	EMS	CAT# 19208
L-lysine	Sigma	CAT# L5501
Goat serum	Thermo Fisher Scientific	CAT# 16210072
SlowFade Gold with DAPI mounting media	Thermo Fisher Scientific	CAT# S36939
Triton X-100	Sigma	CAT# T9284
DAPI	Millipore	CAT# 5.08741.0001
Cytoseal mounting media	Thermo Fisher Scientific	CAT# 8312-4
Ketamine	Victor Medical Company	N/A
Xylazine	Anased	N/A

(*Continued on next page*)

Continued

REAGENT or RESOURCE	SOURCE	IDENTIFIER
Critical Commercial Assays		
Lipofectamine 2000	Thermo Fisher Scientific	CAT# 11668-019
Bradford assay	Bio-Fad	CAT# 5000203
QIAGEN maxi kit	QIAGEN	CAT# 12362
QIAGEN RNeasy mini kit	QIAGEN	CAT# 74104
Affymetrix Rat Genome 230 2.0 arrays	Affymetrix	CAT# 900505
Power Sybr green Cell-to-Ct kit	Thermo Fisher Scientific	CAT# 4402954
RNAscope 2.5 HD—BROWN Manual Assay	ACDbio	CAT# 322300
RNAscope 2.5 HD—multiplex fluorescent Manual Assay	ACDbio	CAT# 320850
Deposited Data		
Affymetrix microarray data	Gene Expression Omnibus	GEO: GSE86595
Experimental Models: Cell Lines		
HEK293 cell line	ATCC	CAT# CRL-1573; RRID: CVCL_0045
HEK293T cell line	ATCC	CAT# CRL-3216; RRID: CVCL_0063
T11D7e2 Hybridoma	ATCC	CAT# TIB-103; RRID: CVCL_F769
Experimental Models: Organisms/Strains		
Rats: CD Sprague-Dawley	Charles Rivers	Stock #001; RRID: RGD_10395233
Experimental Models: Mice		
C57Bl6/J	Jackson Laboratory	Jax# 000664; RRID: IMSR_JAX:000664
C57BL/6N-Gpc4tm2a(EUCOMM)Wtsi/J	Jackson Laboratory	Jax# 025147; MGI:4432991
B6.129S4-Gt(ROSA)26Sortm1(FLP1)Dym/RainJ	Jackson Laboratory	Jax# 009086; RRID: IMSR_JAX:009086
B6.Cg-Gt(ROSA)26Sortm14(CAG-tdTomato)Hze/J	Jackson Laboratory	Jax# 007914; RRID: IMSR_JAX:007914
B6;FVB-Tg(Aldh1l1-cre)JD1884Htz/J	Jackson Laboratory	Jax# 023748; RRID: IMSR_JAX:023748
B6.Cg-Tg(Gfap-cre)77.6Mvs/2J	Jackson Laboratory	Jax# 024098; RRID: IMSR_JAX:024098
B6.Cg-Tg(Gfap-cre)73.12Mvs/J	Jackson Laboratory	Jax# 012886; RRID: IMSR_JAX:012886
Ptprdtm2a(KOMP)Wtsi	EMMA Mouse Repository	EM:06697; MGI:4458607
RORα-IRES-Cre	Prof. D. O'Leary	(Chou et al., 2013)
Pax6-α-Cre	Prof. D. O'Leary	(Marquardt et al., 2001)
B6;129S5-Gpc4tm1Lex/Mmucd	MMRRC	032331-UCD; RRID: MMRRC_032331-UCD
Oligonucleotides		
siRNA targeting rat NP1	Dharmacon	CAT# LQ-098200-02
siControl non-targeting siRNA	Dharmacon	CAT# D-001810-10
qPCR primer GAPDH forward TGCCACTCAGAAGA CTGTGG	IDT	N/A
qPCR primer GAPDH reverse GCATGTCAGATCCA CAATGG	IDT	N/A
qPCR primer NP1 forward TGCAAAGATCACTTCGTCACC	IDT	N/A
qPCR primer NP1 reverse CCAACACACCTTCAAGATCCA	IDT	N/A
qPCR primer NP2 forward AGCGTCTCCTGGA CTTGTAGC	IDT	N/A
qPCR primer NP2 reverse CTTCCCACCAAAG AACAAAGC	IDT	N/A
qPCR primer NPR forward CAACCATCGGCTTG TAGATGA	IDT	N/A
qPCR primer NPR reverse GGAGGAGACCCTTC TCCAGTT	IDT	N/A

(*Continued on next page*)

Continued		
REAGENT or RESOURCE	SOURCE	IDENTIFIER
RNAscope probe: negative control	ACDbio	CAT# 310043
RNAscope probe: double negative control	ACDbio	CAT# 320751
RNAscope probe: triple negative control	ACDbio	CAT# 320871
RNAscope probe: Aldh1l1	ACDbio	CAT# 405891
RNAscope probe: Aldh1l1 channel 2	ACDbio	CAT# 405891-C2
RNAscope probe: Gpc4	ACDbio	CAT# 442821
RNAscope probe: PTPRD	ACDbio	CAT# 474651
RNAscope probe: Tubb3 channel 3	ACDbio	CAT# 423398-C3
Recombinant DNA		
Rat full length glypican 4 with an N-terminal 6 Histidine (His)	This study, Allen lab	Allen et al., 2012
Mouse NP1, full length	This study, Aricescu lab	N/A
Human RPTPσ (full length, short isoform, N-terminally HA-tagged)	This study, Aricescu lab	N/A
Human RPTPδ (full length, short isoform, N-terminally HA-tagged)	This study, Aricescu lab	N/A
Human LAR (full length, N-terminally HA-tagged)	This study, Aricescu lab	N/A
pEGFP-N1, Clontech	Addgene	CAT# 6085-1
Rat RPTPσ	Dharmacon	CAT# MRN1768-202786786
Mouse RPTPδ	Shishikura et al., 2016	N/A
Mouse GluA1, GluA2, GluA3 & GluA4 ATD	This study, Aricescu lab	N/A
Mouse GluA1, GluA3 & GluA4 full length	This study, Aricescu lab	N/A
Rat GluA2, full length	This study, Aricescu lab	N/A
Software and Algorithms		
Imaris	Bitplane	RRID: SCR_007370
ImageJ (Fiji)	NIH	RRID: SCR_003070
Zen	Zeiss	RRID: SCR_013672
AxioVision	Zeiss	RRID: SCR_002677
SDS 2.4	Applied Biosystems	N/A
Expression Console	Affymetrix	N/A
Sigmaplot	Systat	RRID: SCR_003210
Metamorph	Molecular Devices	RRID: SCR_002368
Odyssey Image Studio	Li-Cor	RRID: SCR_014211
Scrubber 2.0	BioLogic Software	http://www.biologic.com.au/
GraphPad Prism	Prism	RRID: SCR_002798
Other		
Glass coverslips, 13mm	Carolina Biological Supply Company	CAT# 633029
Cell culture inserts	Thermo Fisher Scientific	CAT# 353104
Centrifuge column	Thermo Fisher Scientific	CAT# 89896
Vivaspin centrifugal concentrator	Sartorius	CAT# 14558502
4%–12% bolt gels	Thermo Fisher Scientific	CAT# NW04120
PVDF membranes, Immobilon-FL	Millipore	CAT# IPFL00005
Odyssey Infrared Imager	Li-Cor	N/A
Glass slides	Fisher	CAT# 125442
Fluorescence microscope	Zeiss	Axio Imager.Z2
OCT media, Scigen	Fisher	CAT# 4583
TFM tissue freezing media	General Data Healthcare	CAT# TFM-5

(*Continued on next page*)

Continued		
REAGENT or RESOURCE	SOURCE	IDENTIFIER
Cryostat	Hacker Industries	OTF5000
Superfrost Plus glass slides	Fisher	CAT# 1255015
Glass coverslips, 1.5	Fisher	CAT# 12544E
Confocal Microscope	Zeiss	LSM710
Confocal Microscope	Zeiss	LSM780
Confocal Microscope	Zeiss	LSM880
Confocal Microscope	Leica	TCS SP8
HybEZ hybridization system	ACDbio	N/A

CONTACT FOR REAGENT AND RESOURCE SHARING

Further information and requests for resources and reagents should be directed to and will be fulfilled by the Lead Contact, Nicola J. Allen (nallen@salk.edu).

EXPERIMENTAL MODELS

Animals

All animal work was approved by the Salk Institute Institutional Animal Care and Use Committee (IACUC).

Rats

Sprague Dawley rats (Charles Rivers) were maintained in the Salk Institute animal facility, under a 12 hour light:dark cycle with *ad libitum* access to food and water. Rat pups (both male and female) were used at post-natal day (P) 1-2 for preparation of primary cortical astrocyte cultures, and at P5-P7 for preparation of purified immunopanned retinal ganglion cell (RGC) neuronal cultures.

Mice

Mice were maintained in the Salk Institute animal facility, under a 12 hour light:dark cycle with *ad libitum* access to food and water. The following mouse lines were used:

Gpc4 KO

The mouse strain used for this research project, B6;129S5-Gpc4tm1Lex/Mmucd, identification number 032331-UCD, was obtained from the Mutant Mouse Regional Resource Center, a NCRR-NIH funded strain repository, and was donated to the MMRRC by Genentech, Inc. This is the same mouse line we previously characterized (Allen et al., 2012). Mice were backcrossed to C57Bl6/J background (Jax: 000664) for at least 6 generations prior to conducting experiments. For the generation of experimental mice, mice were bred as Gpc4+/− (het) female x Gpc4+/y (WT) male. All experiments were performed using male WT and KO littermates (Gpc4 is on the X chromosome, so experiments compared Gpc4+/y with Gpc4-/y).

Gpc4 Conditional KO

C57BL/6N-Gpc4tm2a(EUCOMM)Wtsi/J were obtained from Jackson Labs (025147). To generate the tm2c mice with a floxed Gpc4 allele, tm2a mice were crossed to the Rosa Flp1 line (Jax: 009086) to remove the neomycin cassette. This produces a line where exon 3 (ENSMUSE00000310079) of Gpc4 (ENSMUST00000033450.2) is floxed. Upon exposure to cre recombinase this will remove exon 3 leading to a frameshift and premature stop codon, which is predicted to lead to nonsense mediated decay of the mRNA. Characterization of this line is shown in Figure 6. Mice were maintained as homozygous for floxed Gpc4 on a C57Bl6/J background, and crossed to mice expressing cre recombinase (see below) for experiments.

RPTPδ Conditional KO

We thank the Wellcome Trust Sanger Institute Mouse Genetics Project (Sanger MGP) and its funders for providing the mutant mouse line (Ptprdtm2a(KOMP)Wtsi). Funding and associated primary phenotypic information may be found at http://www.sanger.ac.uk/science/collaboration/mouse-resource-portal. To generate the tm2c mice with a floxed RPTPδ allele, tm2a mice were crossed to the Rosa Flp1 line (Jax: 009086) to remove the neomycin cassette. This produces a line where exon 22 (ENSMUSE00000526394) of the consensus sequence (ENSMUST00000173376.7) is floxed, which is within the D1 phosphatase domain of RPTPδ (Figure S7A). Upon exposure to cre recombinase this will remove exon 22 leading to a frameshift and premature stop codon, which is predicted to lead to nonsense mediated decay of the mRNA. Characterization of this line is shown in Figures 7 and S7. This is a similar targeting strategy as used to generate mice with a null allele of RPTPδ, where exon 20 was targeted at the start of the phosphatase domain (Uetani et al., 2000). Mice were maintained as homozygous for floxed RPTPδ on a C57Bl6/J background, and crossed to mice expressing cre recombinase for experiments.

RORα-IRES-Cre

RORα-IRES-Cre mice were obtained from Dennis O'Leary at the Salk Institute, and described in Chou et al. (2013). Mice were backcrossed to C57Bl6/J (Jax:000664) for 3-4 generations before being crossed to the RPTPδ flox line. Expression of cre recombinase in

the thalamus was confirmed by crossing the RORα cre mouse with a tdTomato reporter mouse line (Jax: 007914) (Figures S7D–S7F). For the generation of experimental mice, mice were bred as RPTPδ$^{f/f}$;cre-ve x RPTPδ$^{f/f}$;RORα cre+ve. Homozygous flox RPTPδ mice positive or negative for RORα cre were compared in each experiment; both male and female mice were used.

Pax6-α-Cre

Pax6-α-Cre mice were generated by Peter Gruss lab at the Max Planck Institute of Biophysical Chemistry, Gottigen, Germany as described in Marquardt et al. (2001) and obtained from Dennis O'Leary lab at the Salk Institute. Mice were backcrossed to C57Bl6/J (Jax:000664) for 3-4 generations before being crossed to the RPTPδ line. Expression of cre recombinase in the retina was confirmed by crossing Pax6-α-cre mice with a GFP reporter mouse line (Figures S7B and S7C). For the generation of experimental mice, mice were bred as RPTPδ$^{f/f}$;cre-ve x RPTPδ$^{f/f}$;Pax6-α-cre+ve. Homozygous flox RPTPδ mice positive or negative for Pax6-α-cre were compared in each experiment; both male and female mice were used.

GFAP-Cre 77.6

GFAP-Cre 77.6 mice were obtained from Jackson Labs (024098). Expression of cre recombinase in astrocytes was determined by crossing the cre mouse with a tdTomato reporter mouse line (Jax: 007914) (Figures S6A and S6B).

GFAP-Cre 73.12

GFAP-Cre 73.12 mice were obtained from Jackson Labs (012886). Expression of cre recombinase in astrocytes was determined by crossing the cre mouse with a tdTomato reporter mouse line (Jax: 007914) (Figures S6A and S6B).

Aldh1l1-Cre

Aldh1l1-Cre mice were obtained from Jackson Labs (023748). Expression of cre recombinase in astrocytes was determined by crossing the cre mouse with a tdTomato reporter mouse line (Jax: 007914) (Figures S6A and S6B). For the generation of experimental mice, mice were bred as Gpc4$^{f/f}$ cre-ve female x Aldh1l1cre+ve male. Hemizygous flox Gpc4 male mice (Gpc4$^{f/y}$) positive or negative for Aldh1l1 cre were compared in each experiment.

Tissue Collection

Mice were collected at P6-7 unless stated otherwise in the text. For each experiment WT and KO littermates were collected and analyzed at the same time for comparison.

Cell Culture

Retinal Ganglion Cell (RGC) Neuron Purification and Culture

RGC purification and culture was performed as described (Allen et al., 2012; Ullian et al., 2001; Winzeler and Wang, 2013). Briefly, retinas from P5-P7 rat pups of both sexes were removed and placed in DPBS (HyClone Cat. SH30264). Retinas were digested with Papain (Worthington Cat. PAP2 3176; 50 units) for 30 min at 34C, triturated with Low OVO (15 mg/ml trypsin inhibitor (Worthington Cat. LS003086)), then High OVO (30 mg/ml trypsin inhibitor (Worthington cat. LS003086)) solutions. The cell suspension was then added to lectin (Vector Cat. L-1100) coated Petri dishes to pull down microglia and fibroblast cells for 5-10 min at room temp. The remaining cells were then added to T11D7 hybridoma supernatant coated Petri dishes for 40 min at room temp, which specifically binds RGCs. After washing off the non-binding cells with DPBS, pure RGCs were released by trypsin treatment (Sigma Cat. T9935) to cleave cell-antibody bond, and collected. RGCs were plated on glass coverslips (Carolina Biological Supply Company Cat. 633029) coated with PDL (Sigma Cat. P6407) and laminin (Cultrex Trevigen Cat. 3400-010-01) at a density of 20,000-30,000 cells/well in a 24 well plate or 10,000 cells/well in 48 well plates. RGCs were maintained in the following media: 50% DMEM (Life tech Cat. 11960044); 50% Neurobasal (Life Tech Cat. 21103049); Penicillin-Streptomycin (LifeTech Cat. 15140-122); glutamax (Life Tech Cat. 35050-061); sodium pyruvate (Life Tech Cat. 11360-070); N-acetyl-L-cysteine (Sigma Cat. A8199); insulin (Sigma Cat. I1882); triiodo-thyronine (Sigma Cat. T6397); SATO (containing: transferrin (Sigma T-1147); BSA (Sigma A-4161); progesterone (Sigma P6149); putrescine (Sigma P5780); sodium selenite (Sigma S9133)); and B27 (see Winzeler and Wang, 2013 for recipe). For complete growth media, the media was supplemented with BDNF (Peprotech Cat. 450-02), CNTF (Peprotech Cat. 450-13), and forskolin (Sigma Cat. F6886). The next day, half of the media was replaced with media containing FUDR (13 μg/μl final concentration Sigma Cat. F0503) to inhibit fibroblast growth. Cells were fed by replacing half of the media with fresh equilibrated media every 3-4 days. RGCs were maintained at 37C/10%CO2 and kept in culture for at least 7 days prior to treatment to allow for full process outgrowth.

Astrocyte Preparation and Culture

Primary astrocytes from rat cortex were prepared as described (Allen et al., 2012; McCarthy and de Vellis, 1980). Briefly, the cerebral cortex from P1-P2 rat pups were removed and placed in DPBS (HyClone Cat. SH30264). The meninges and hippocampi were removed and discarded. The remaining cortices were diced and digested with Papain (Worthington Cat. LS003126; 330 units) for 1 hr and 15 min in 37C 10% CO2 cell culture incubator. Cells were triturated in Low OVO and then High OVO containing solutions, and plated in PDL coated 75cm tissue culture flasks. 3 days after plating, flasks were manually shaken to remove upper cell layers which contained mostly non astrocytic cells. 2 days after shake off, ARA-C (10 μM final concentration; Sigma Cat. C1768) was added for 48 hours to inhibit the other proliferating cells, which divide faster than astrocytes. Finally, astrocytes were plated in 15 cm cell culture plates coated with PDL at 2-3 million cells/dish and passaged once a week. Astrocytes were maintained at 37C/10%CO2 and kept in culture for 3-4 weeks. Astrocyte culture medium was DMEM (Life tech Cat. 11960044) supplemented with 10% Heat inactivated FBS (LifeTech Cat. 10437028), Penicillin-Streptomycin (LifeTech Cat. 15140-122), glutamax (Life Tech Cat. 35050-061), sodium pyruvate (Life Tech Cat. 11360-070), hydrocortisone (Sigma Cat. H0888), and N-acetyl-L-cysteine (Sigma Cat. A8199).

Depending on the specific requirement of the experiments, astrocytes were either plated on cell culture inserts (Falcon Cat. 353104) at 50,000 cell/ insert or kept in 15 cm dishes and used to prepare astrocyte conditioned media (ACM).

HEK293 Cells

HEK293 cells (ATCC Cat. CRL-1573, without the T antigen) were obtained from the Evans lab at the Salk Institute. Cell lines were maintained at 37C/5%CO2 in the following media: DMEM (Life tech Cat. 11960044) supplemented with 10% Heat inactivated FBS (LifeTech Cat. 10437028), 1% Penicillin-Streptomycin (LifeTech Cat. 15140-122), 1% glutamax (Life Tech Cat. 35050-061) and 1% sodium pyruvate (LifeTech Cat. 11360-070), and passaged once a week.

HEK293T Cells

HEK293T cells (ATCC Cat CRL-3216) were cultured in DMEM (GIBCO Cat. 31966-021) supplemented with 10% Fetal Bovine Serum (FBS) (GIBCO Cat. No. 10270-106), 1x MEM Non-Essential Amino Acids (GIBCO Cat. 11140-035) and 2mM L-Glutamine (GIBCO Cat. 25030-024).

METHOD DETAILS

cDNA Constructs

The following cDNA constructs were used, and transfected into cells using lipofectamine 2000 (Life Tech Cat. 11668-019) unless stated otherwise in the text, according to the manufacturer's instructions.

GFP: pEGFP-N1 mammalian expression vector (Clontech, Addgene cat. 6085-1)

Glypican 4: Rat full length glypican 4 with an N-terminal 6 Histidine (His) tag sub-cloned into pAPtag5 vector (GenHunter) between the SfiI and XhoI sites (Allen et al., 2012).

The full-length RPTP constructs were sub-cloned into the pHLsec vector between AgeI and KpnI restriction sites (Aricescu et al., 2006), downstream of the secretion signal sequence provided by the vector and a sequence encoding the HA-tag (YPYDVPDYA).

Human RPTPσ (full length, short isoform, N-terminally HA-tagged; UniProt ID: Q13332-7, residues: 30-1501);

Human RPTPδ: (full length, short isoform, N-terminally HA-tagged; UniProt ID: Q3KPI9, residues: 21-1496);

Human LAR: (full length, N-terminally HA-tagged; UniProt ID: P10586, residues: 30-1907).

Mouse RPTPδ: construct used to validate the specificity of the RPTPδ antibody (Figure S7K) was as described in Shishikura et al. (2016).

Rat RPTPσ: Dharmacon (Cat. MRN1768-202786786)

Mouse neuronal pentraxin 1: (NP1, UniProt ID: Q62443, residues: 23-432) was synthesized and codon optimized for expression in human cells, and cloned into the pHL-Avitag3 vector (Aricescu et al., 2006) between AgeI and KpnI sites.

Recombinant Proteins

His-Tagged Glypican 4

His-tagged glypican 4 was purified as described (Allen et al., 2012). Briefly, HEK293 cells were transfected with His-glypican 4 cDNA using lipofectamine 2000 (Life Tech Cat. 11668-019), according to the manufacturer's instructions. Cells were then placed in conditioning media (see ACM preparation) and incubated for 3 days. The secreted glypican 4 was purified by incubating conditioning media with Ni-coated agarose beads over night at 4C. The next day the beads were washed 5 times with cold DPBS supplemented with protease inhibitors cocktail (Roche Cat. 04693132001) to remove nonspecific binding and loaded onto a centrifuge column (Thermo Cat. 89896). Glypican 4 was eluted from the beads by washing the column with DPBS with 250 mM imidazole (high concentration of imidazole disrupts the bond between Histidine and Nickel). Finally, 2 media substitution steps were performed using concentrators (Sartorius Cat. 14558502) to achieve purified glypican 4 diluted in sterile DPBS. Amount and quality of protein were assessed by western blot (data not shown), and the amount of glypican 4 to treat RGCs was matched to the amount measured in 25 μg of ACM, as described in Allen et al. (2012). Purified glypican 4 was kept at 4C and used within 3-4 weeks.

Recombinant Mouse Thrombospondin1 (TSP1)

Recombinant mouse Thrombospondin1 (TSP1) was purchased from RandD (Cat. NP_035710); aliquoted, snap frozen and stored at −80C. The amount of TSP1 added to cells was experimentally determined by adding different doses to RGCs and performing immunostaining for synaptic markers. The amount which induced the most robust synapse formation was chosen for subsequent experiments and was 0.25 μg/ml.

RPTP Wedge Domain-Blocking Peptides

RPTP wedge domain-blocking peptides (Lang et al., 2015) were synthesized in the Salk Peptide Synthesis Core using standard Fmoc chemistry, and subsequently HPLC-purified to > 95% and converted to the acetate salt at 21st Century Biochemicals, Inc. The lyophilized peptides were rehydrated in sterile double distilled water, aliquoted and snap frozen. Aliquots were kept at −80C until use. 2.5 μM final concentrations were used from each peptide. Each peptide has a TAT sequence (GRKKRRQRRR) which makes it cell permeant.

The following sequences of peptides were used:

Sigma: GRKKRRQRRRCDMAEHMERLKANDSLKLSQEYESI-amide

Scrambled Sigma: GRKKRRQRRRCIREDDSLMLYALAQEKKESNMHES-amide

LAR: GRKKRRQRRRCDLADNIERLKANDGLKFSQEYESI-amide

Delta: GRKKRRQRRRCELADHIERLKANDNLKFSQEYESI-amide

Alexa488-Tagged NP1

Full length mouse neuronal pentraxin 1 (NP1, UniProt ID: Q62443, residues: 23-432) was synthesized and codon optimized for expression in human cells. The construct was cloned into the pHL-Avitag3 vector (Aricescu et al., 2006) between AgeI and KpnI sites and transfected into HEK293T cells using 1mg/ml Polyethylenimine (PEI) (Sigma Cat. 408727) as described (Aricescu et al., 2006). Conditioned medium was harvested two days after transfection, centrifuged at 5000xg for 30 minutes and sterile filtered (0.22 μm Stericup, Millipore Cat. SCGPU05RE/Steri). The protein was affinity-purified using Talon beads (Clontech Cat. 635503) followed by size exclusion chromatography on a Superose 6 Increase 3.2/300 column (GE Healthcare Cat. 29-0915-98). Peak fractions were pooled together and concentrated to 1 mg/ml. 100 μL pure protein was labeled with Alexa Fluor 488 using a kit from Invitrogen (Cat. A20181). Labeled protein was protected from light, aliquoted and flash-frozen in liquid nitrogen until use.

GluA1-Fab Generation

Hybridoma Generation

Custom peptide antigens corresponding to helix α9 and the loop leading up to helix α10 of the amino-terminal domain (ATD) of mouse AMPA-type glutamate receptor subunit 1 (GluA1; *C*DTIPARIMQQWRTSDARDHTRVDWKR, 27aa; UniProt ID: P23818; residues 242-267) were synthesized and conjugated to the immunogenic keyhole limpet hemocyanin (KLH) via an N-terminal cysteine. Five BALB/c mice were intraperitoneally injected with 50 μg immunogen emulsified with Freund's Complete Adjuvant. Two weeks later, mice received a first boost of 25 μg immunogen in Freund's Incomplete Adjuvant. Two subsequent boosters were given at three week intervals. Test bleeds were collected a day before initial immunization and seven days after each booster injection. Serum samples were screened against recombinant mouse heteromeric GluA1/GluA2 AMPA receptors expressed on the surface of live HEK293T cells (Lai et al., 2009). The host animal with positive serum antibodies was used as a splenic B cell donor to produce a hybridoma for mouse monoclonal anti-GluA1 antibodies.

Anti-GluA1 Fab Fragment Generation

Total RNA was isolated from cultured GluA1 hybridoma generated above, and first-strand cDNA synthesized from the purified RNA by RT-PCR with random hexamers (SuperScript™ III First-Strand Synthesis System, Thermo Fisher Cat. 18080051). cDNA constructs coding for the γ heavy chain, fused C-terminally with a hexa-histidine (His6) tag, and the κ light chain of mouse IgG antibody Fab fragments were amplified by PCR (Pyrobest Polymerase, Takara Bio Cat. R005A) using a set of degenerate primers (Kettleborough et al., 1993). The resulting PCR products were cloned into the pHLsec vector (Aricescu et al., 2006).

GluA1-Fab Fragment γ Heavy Chain

The following degenerate primer pair was used for the PCR amplification of mouse anti-GluA1 Fab fragment γ heavy chain (degenerate bases underlined and variations indicated beneath):

FP: 5′-CGCACCGGTCAGCTGCAGCAGTCTGGA-3′
T GT A

RP: 5′-ATACTCGAGTCATTAATGGTGGTGATGGTGATGAGTACCGCAGTC TCTGGGGACGATCTTCTTGTCGACCTTGGTGCTG CTGGCCGGGTG-3′

GluA1-Fab Fragment κ Light Chain

The following degenerate primer pair was used for the PCR amplification of mouse anti-GluA1 Fab fragment κ light chain (degenerate bases underlined and variations indicated beneath):

FP: 5′-CGCACCGGTGATGTTGTGATGACCCAAACTCCA-3′
AC T

RP: 5′-ATACTCGAGTCATTAACACTCATTCCTGTTGAAGC-3′

Mut-Fab Fagment

A control protein was engineered by introducing a mutation to the complementarity-determining region (CDR) of the Fab fragment. Three residues on the hypervariable H3 loop of the γ heavy chain were replaced with an elongated sequence (CGRWDDMDY to CGRNWEGWYMDY). cDNA coding for the mutant γ heavy chain was cloned into the pHLsec vector (Aricescu et al., 2006).

Production and Purification of Fab Fragments

For large-scale protein production, proteins were expressed by transient transfection in HEK293T cells in a 1:1 heavy chain:light chain stoichiometric ratio. Five days post-transfection, the conditioned medium was collected and buffer-exchanged using a QuixStand benchtop diafiltration system (GE Healthcare, Cat. 56-4107-78) and proteins were purified by immobilized metal-affinity chromatography (IMAC) using pre-packed Ni-Sepharose columns (HisTrap™ HP, GE Healthcare, Cat. 17-5248-02). Proteins were concentrated and further purified by size-exclusion chromatography (SEC; Superdex® 200 16/60 PG HiLoad column, GE Healthcare, Cat. 28989335) in Dulbecco's phosphate-buffered saline (DPBS) with Ca^{2+} and Mg^{2+} (Sigma-Aldrich, Cat. D8662).

GluA1-Fab Fragment Specificity Analysis

Surface Plasmon Resonance (SPR)

cDNA constructs coding for the amino-terminal domains of mouse GluA1 (UniProt ID: P23818, residues: 19-393), GluA2 (UniProt ID: P23819, residues: 22-400), GluA3 (UniProt ID: Q9Z2W9, residues: 24-404) and GluA4 (UniProt ID: Q9Z2W8, residues 21-401) were cloned into the pHLsec-Avitag3 vector (Aricescu et al., 2006) between the AgeI and KpnI sites, resulting in proteins carrying C-terminal biotin ligase (Avitag) (Howarth and Ting, 2008) and hexa-His tags. Constructs were co-transfected with pDisplay-BirA-ER

(Addgene plasmid 20856) to express an ER-resident biotin ligase for *in vivo* biotinylation in HEK293T cells in small-scale 6-well plates in a 3:1 pHLsec:pDisplay stoichiometric ratio. A concentration of 100 μM D-biotin was maintained in the expression medium to ensure near-complete biotinylation of the recognition sequence. After 48 hours of expression, conditioned medium was collected and dialyzed against 10mM Tris pH 7.4, 150mM sodium chloride, 2mM calcium chloride, 2mM magnesium chloride and 0.005% (v/v) Tween-20.

SPR experiments were performed on a Biacore™ T200 machine (GE Healthcare) operated at a data collection frequency of 10 Hz; i.e., a temporal resolution of 0.1 s. Streptavidin (Sigma-Aldrich Cat. S4762) was chemically coupled via amine coupling chemistry onto CM5 chips to a response unit (RU) level of 2000 RU. Then, the biotinylated ATD constructs were captured to a level of 500 RU. The immobilized ligand sensor surface was equilibrated in SPR running buffer supplemented with 1.0g/L bovine serum albumin (BSA) to attain a stable baseline. 14 concentrations of GluA1-Fab and Mut-Fab were prepared in a two-fold dilution series from a 10 μM stock concentration. Injections were performed in order of ascending concentration, followed by descending concentration, i.e., each concentration was sampled in duplicate within the same experiment. Each analyte sample was injected for 180 s at a flow rate of 10 μl/min, followed by a 200 s dissociation phase. The surfaces were regenerated using a 600 s injection of 50mM L-Arginine/L-Glutamate at 20 μl/min. For every two analyte binding cycles, a buffer injection was performed, allowing for double referencing of the binding responses (Myszka, 1999). No regeneration was performed after buffer injections. Equilibrium binding analysis was performed using Scrubber 2.0 (BioLogic Software, http://www.biologic.com.au/) and data was fitted to a 1:1 Langmuir binding model in Prism 6 (Graphpad). Dissociation constants of GluA1-Fab and Mut-Fab and the maximum response were evaluated using SigmaPlot (Systat Software Inc.).

Immunofluorescence

cDNA constructs of the mouse GluA1 (*flip* isoform, UniProt ID: P23818, residues: 19-907), rat GluA2 (identical to the mouse ortholog in the extracellular regions, *flip* isoform, UniProt ID: P19491, residues: 22-883), mouse GluA3 (*flip* isoform, UniProt ID: Q9Z2W9, residues: 24-888) and mouse GluA4 (*flip* isoform, UniProt ID: Q9Z2W8, residues 21-902) subunits were cloned into the pHLsec vector (Aricescu et al., 2006) between the AgeI and the XhoI sites. N-terminal HA-tag (YPYDVPDYA, for GluA1, GluA3 and GluA4) or FLAG-tag (DYKDDDDK, for GluA2) sequences were present immediately upstream of the first mature polypeptide residues. Plasmids were transfected into HEK293T cells using Lipofectamine 2000 (Thermo Fisher Scientific, Cat. 11668019). One day post transfection, cells were incubated with 5μM GluA1-Fab, rabbit anti-HA (Sigma-Aldrich, Cat. H6908, 10 μg/ml) or rabbit anti-Flag (Sigma-Aldrich, Cat. F7425, 5 μg/ml) antibodies in tissue culture medium, on ice, for one hour. Afterward, cells were washed with Dulbecco's phosphate-buffered saline (DPBS) with Ca^{2+} and Mg^{2+} (Sigma-Aldrich, Cat. D8662), and then fixed for 5 minutes at room temperature with 3% paraformaldehyde (Sigma-Aldrich, Cat. P6148). Cells were then blocked with antibody buffer (150mM NaCl; 50mM Tris pH 7.4; 100mM L-lysine; 1% BSA) for 30 minutes at room temperature, and incubated with secondary antibodies, goat anti-mouse IgG (Fab specific)-FITC (Sigma-Aldrich, Cat. F4018, dilution 1:750) or Alexa Fluor 647 goat anti-rabbit IgG (H+L) (Invitrogen, CAt. A21245, 5 μg/ml) in antibody buffer supplemented with 10% goat serum, for 1 hour at room temperature. Unbound antibodies were removed by washing with DPBS (three times, 5 minutes each, at room temperature), with one final wash containing Hoechst 33342 nuclear stain (stock 10mg/ml, use 1:100,000, Sigma-Aldrich, Cat. B2261). Immunofluorescence was detected using a Leica TCS SP8 WLL Confocal SMD microscope and images were processed in Fiji (ImageJ).

Treating RGC Neurons with Fab

2.5 μM final concentration of GluA1-Fab or Mut-Fab were used to treat RGCs.

Treating RGC Neurons with Purified Alexa488-Tagged NP1 and Fab

RGCs were treated with NP1 at a final concentration of 0.4 μg/ml (0.008 μM assuming M.W. of 47 kDa) together with 2.5 μM final concentration of GluA1-Fab or Mut-Fab for 24 hours prior to fixation and imaging. 24 hours later cell tracker red (Life Tech Cat. C34552) was added to the cell culture media (see surface immunostaining of RGCs) for 30 min at 37C, followed by 3 washes with DPBS and 5 min fixation with 4% PFA at room temp. Then coverslips were washed 3 times with PBS and mounted on slides and imaged as described in the cell surface immunostaining section (see below).

Preparation of Astrocyte Conditioned Media (ACM)

Astrocytes were plated on 15 cm tissue culture plates and allowed to reach ~90% confluency. Cells were washed 3X times with warm DPBS to remove serum containing cell culture media and placed in a low protein conditioning media containing (50% DMEM, 50% Neurobasal media; Penicillin-Streptomycin; Glutamax and sodium pyruvate). Astrocytes were incubated for 3-5 days, after which media containing the secreted proteins was collected and concentrated 50-fold using 10 kDa cutoff concentrators (Sartorius Cat. 14558502). Protein concentration was measured using the Bradford method. The amount of ACM fed to RGCs was 50 μg/ml.

Western Blot

Samples were heated in reducing loading dye (Thermo Cat. 39000) for 45 min at 55C. Samples were resolved on 4%–12% bis-tris or bolt gels (Invitrogen Cat. NW04120) for 30-40 min at 150-200V. Proteins were transferred to PVDF membranes at 100V for 1 hr, then blocked in 1% casein (Biorad Cat. 1610782) in TBS (Bioworld Cat. 105300272) blocking buffer for 1 hr at room temp on a shaker. Primary antibodies were applied overnight at 4C diluted in blocking buffer. The next day, membranes were washed 3X 10 min

with TBS-0.1%Tween and secondary antibody conjugated to Alexa fluor 680 was applied for 2 hr at room temp. Bands were visualized using the Odyssey Infrared Imager (Li-Cor) and band intensity analyzed using the Image Studio software (Li-Cor).

RNA Analysis from RGCs *In Vitro*

Microarray

For microarray experiments RGCs were grown in 6-well plates at a density of 250,000 cells/well, 2 wells were pooled per experimental condition. RGCs were treated with Gpc4 or TSP1 for 12 hours before collection of RNA. RNA was harvested and purified using the QIAGEN RNeasy mini kit with on column DNA digestion (QIAGEN Cat. 74104), following manufacturer instructions. RNA was processed, hybridized and scanned following manufacturer instructions for Affymetrix Rat Genome 230 2.0 arrays (Affymetrix Cat. 900505). The experiment was repeated on 2 separate RGC cultures. Data was processed and analyzed using Affymetrix Expression Console software. Raw data has been deposited at GEO. Full data is provided in Table S1.

qRT-PCR

For qPCR experiments RGCs were grown in 48 well plates, 3 wells were pooled per experimental condition. RGCs were treated with Gpc4 for the time indicated in the text (1 hour, 4 hours, 12 hours or 18 hours). RGCs were then lysed and cDNA prepared using the Cell-to-Ct kit (Life Tech Cat. 4402954). Reverse transcription reaction contained the following steps: 1 cycle at 37°C for 60 minutes, 1 cycle at 95°C for 5 min. The cDNA was then used for the qPCR reaction. Rat Glyceraldehyde 3-phosphate dehydrogenase (GAPDH) was used as a control housekeeping gene to normalize the Ct values obtained for the candidate gene primers. The qPCR reaction was performed using the power SYBR green PCR master mix (Life Tech Cat. 4402954) and contained: one enzyme activation step at 95°C for 10 minutes, 40 PCR cycles at 95°C for 15 s and at 60°C for one minute. Data was analyzed using the SDS 2.4 software (Applied Biosystems).

Immunostaining RGCs *In Vitro*

Treatment of RGCs with Synaptogenic Factors

RGCs were treated with purified proteins or ACM or astrocyte inserts for 6 days to ensure maximal effect on synapse formation and GluA1 clustering, unless stated otherwise in the text. In each experiment the control group is RGCs grown in cell culture media alone.

Treatment of RGCs with Transcription Inhibitor

RGCs were treated with Actinomycin D (ActD) (Fisher Cat. 11805017) for 4 or 12 hours. Actinomycin D concentration was 1 μg/ml.

Knockdown of NP1 in RGCs Using siRNA

siRNA against rat NP1 was purchased from Dharmacon (set of 4 ON-target plus siRNA smart pool, Cat. LQ-098200-02-0002). ON-TARGETplus Non-targeting Pool (Cat. D-001810-10-05) was used as control and labeled siControl. RGCs at DIV 7-8 were transfected with siNP1 or siControl together with GFP to mark transfected cells using lipofectamine 2000 reagent according to manufacturer instructions. The next day cells were treated with Gpc4 or vehicle for additional 6 days (as above).

Cell Surface iImmunostaining

To visualize cell surface proteins without permeabilizing the cells, RGCs were incubated with primary antibody along with fluorescent cell tracker red (Life Tech Cat. C34552) to visualize cell processes for 30 min at 37C in cell culture incubator. After that, cells were washed 3 times with 34C DPBS, fixed for exactly 5 min at room temp with 34C 4% PFA (EMS Cat. 50980487), and blocked with antibody buffer (150 mM NaCl; 50 mM Tris; 100 mM L-lysine; 1% BSA; pH 7.4) supplemented with 50% heat inactivated normal goat serum (Life tech Cat. 16210072) for 30 min at room temp. The short fixation time as well as absence of Triton X-100 from the blocking buffer were necessary to prevent permeabilization of the cells. Finally, secondary antibody conjugated to Alexa fluor 488 diluted in antibody buffer with 10% goat serum was applied for 1 hr at room temp. Coverslips were washed 3x with PBS and mounted using the SlowFade Gold with DAPI mounting media (Life Tech Cat. S36939) on glass slides (Fisher Cat. 125442) and sealed with clear nail polish. Mounted coverslips were imaged using fluorescent microscope (Ziess; Axio Imager.Z2) at 63X magnification. 16 bit images at 1388 X 1040 pixels were acquired using AxioCam HR3 camera (Zeiss). All experimental groups per given experiment were imaged on the same day using set exposure as determined for the positive control group in each experiment (RGCs+ astrocytes; RGCs + ACM; RGCs + Gpc4; RGCs +TSP1; RGCs siControl+Gpc4). Cells were detected using the cell tracker red channel, and an image of the cell surface puncta was obtained. 20-30 cells across 3 coverslips were imaged for each group. For GluA1 surface staining experiments images were analyzed using the Integrated Morphometry application of the Metamorph software (Molecular devices). For each cell, a region of interest containing the cell body and proximal dendrites was outlined and cropped. Positive puncta were determined by manually thresholding the GluA1 channel, then the number of puncta within that region was counted. All cells were thresholded in the same way. For NP1 surface staining, analysis was performed using the Puncta Analyzer plugin in ImageJ. Similarly to GluA1 analysis, a region of interest was cropped, and then number of puncta counted for each cell. Each experiment was performed at least 3 times using different RGC preps.

Synaptic Staining

To visualize pre and post synaptic compartments RGCs were washed with 34C DPBS, then fixed at room temp with 4% PFA (EMS Cat. 50980487) for 10 min. Cells were then washed 3 times with PBS and blocked and permeabilized in antibody buffer (150 mM NaCl; 50 mM Tris; 100 mM L-lysine; 1% BSA; pH 7.4) supplemented with 50% heat inactivated normal goat serum (Life tech Cat. 16210072) and 0.2% Triton X-100 (Sigma Cat. T9284) for 30 min at room temp. Primary antibody was applied overnight at 4C diluted in antibody buffer with 10% goat serum. The next day, cells were washed 3 times with PBS and secondary antibody conjugated to

Alexa fluor was applied for 1 hr at room temp. Coverslips were washed 3x with PBS and mounted using the SlowFade Gold with DAPI mounting media (Life Tech Cat. S36939) on glass slides (Fisher Cat. 125442) and sealed with clear nail polish. Mounted coverslips were imaged using fluorescent microscope (Ziess; Axio Imager.Z2) at 63X magnification. 16 bit images at 1388 X 1040 pixels were acquired using AxioCam HR3 camera (Zeiss). All experimental groups per given experiment were imaged on the same day using set exposure as determined for the positive control group in each experiment (RGCs+ astrocytes; RGCs + ACM; RGCs + Gpc4; RGCs +TSP1; RGCs siControl+Gpc4). Cells were detected using the DAPI channel, and image of pre and postsynaptic puncta was obtained for each cell. For each group 20-30 cells across 3 coverslips were imaged. Images were analyzed using the Puncta Analyzer plugin of ImageJ as described (Allen et al., 2012). For each cell, a region of interest was determined as a circle containing the cell body and proximal dendrites. Positive pre and post synaptic puncta were determined by thresholding each individual channel, and a synapse was then calculated as the colocalization of pre and post synaptic puncta. All cells were thresholded in the same way. All experiments were repeated at least 3 times using different RGC preps.

Imaging and Analysis of Synapses within Transfected Axons in RGCs

For experiments testing the number of synapses which colocalize with axons expressing RPTPs (Figure 3), RGCs were transfected with cDNA expressing an HA-tagged RPTP of interest at DIV 7 using lipofectamine 2000. GFP transfection was used as a control condition. The next day RGCs were treated with purified His-Gpc4 for 6 days to induce synapse formation. RGCs were then immunostained with antibodies against pre- and post-synaptic markers, as well as an antibody against the HA tag to visualize the axons overexpressing the RPTP. Cells were imaged using fluorescent microscope (Ziess; Axio Imager.Z2) at 63X magnification. 16 bit images at 1388 X 1040 pixels were acquired using AxioCam HR3 camera (Zeiss). Expressing axons were visualized in the green channel, and were selected by morphology as long processes wrapping around a non-expressing cell body (the non-expressing cell body did not show staining in the green channel). For each cell a z stack image was taken (optical slice 0.5 um) to cover the entire axon wrapped around the cell. All experimental groups per given experiment were imaged on the same day using set exposure as determined for the positive control group in each experiment (GFP expressing RGCs + Gpc4). 3D z stack images were analyzed using Imaris software (Bitplane). The GFP or RPTP expressing axon was selected based on intensity in the green channel and cropped. Pre and post synaptic puncta were selected based on size and intensity by thresholding each image in the same way. Then number of synapses (the amount of colocalized pre and post synaptic puncta) was counted for each cell. Finally, only the synapses which overlapped with the axon area were selected for each cell. To account for the differences in size of analyzed axonal area, the total volume of the axon was calculated for each cell. No significant difference between the total axonal volumes was found between the experimental groups (data not shown). Example images in Figure 3 and Figure S3A show maximal intensity projection images. All experiments were repeated at least 3 times using different RGC preps.

Mouse Brain Tissue Preparation and Immunostaining

Tissue Collection and Processing

Mice were anaesthetized by I.P. injection of 100 mg/kg Ketamine (Victor Medical Company)/20 mg/kg Xylazine (Anased) mix and transcardially perfused with PBS. To obtain fresh frozen tissue used for RNAscope *in situ* experiments and immunostaining, brains were removed and immediately embedded in OCT media (Scigen Cat. 4583), frozen in dry ice-ethanol slurry solution, and stored at −80C until use. To obtain fixed tissue used for immunostaining in Figures 7A, 8, S6C, S7B, and S7D–S7F, PBS perfusion was immediately followed by perfusing the mice with 4% PFA. Brains and/or eyes were removed and incubated in 4% PFA overnight at 4C, then washed 3X 5 min with PBS, and cryoprotected in 30% sucrose for 2-3 days, before being embedded in TFM media (General data healthcare Cat. TFM-5), frozen in dry ice-ethanol slurry solution, and stored at −80C until use. Brains were sectioned using a cryostat (Hacker Industries OTF5000) in sagittal or coronal orientations depending on experimental needs at a slice thickness of 16 μm. Eyes were sectioned through the lens-optic nerve axis at a slice thickness of 16 μm. Sections were mounted on Superfrost plus slides (Fisher Cat. 1255015).

Immunostaining and Imaging in Mouse Brain Tissue

Immunostaining of fresh frozen tissue was performed immediately following brain sectioning. Sections were fixed for 8 min with methanol at −20C, then washed with PBS (3X 5 min) and fixed for 8 min with 4% PFA at room temp, then washed with PBS (3X 5 min). Sections were then blocked for 1 hr at room temp in blocking buffer containing antibody buffer (100 mM L-lysine and 0.3% Triton X-100 in PBS) supplemented with 10% heat inactivated normal goat serum. Primary antibodies diluted in antibody buffer with 5% goat serum were incubated overnight at 4C. The next day sections were washed 3X 5 min with PBS with 0.2% Triton X-100 and secondary antibodies conjugated to Alexa fluor were applied for 2 hr at room temp. Slides were mounted with the SlowFade Gold with DAPI mounting media (Life Tech Cat. S36939), covered with 1.5 glass coverslip (Fisher Cat. 12544E) and sealed with clear nail polish. Slides were imaged using Zeiss LSM 780 confocal microscope at 63X magnification. Experiments shown in Figures 6J–6M slides were imaged using Zeiss LSM 880 confocal microscope using same magnification and imaging parameters. All imaging of a given experiment was obtained on the same day (WT and KO), with set exposure as determined for the control group (WT genotype). For each section a 1420 X 1420 pixel 3 μm thick z stack image was obtained (optical slice 0.33 μm; 9 slices per 3 μm stack). 3D z stack images were analyzed using Imaris software (Bitplane). Images were background subtracted in the same way and positive puncta of NP1, RPTPδ or VGlut2 were selected by size and intensity by thresholding the images in the same way for each section. Then colocalization between NP1 and VGlut2 or RPTPδ and VGlut2 was calculated. Puncta were considered colocalized if the distance between them was $\leq$ 0.5 μm. Number of colocalized puncta was obtained and compared between the experimental groups. A minimum of

3 sections per mouse were imaged for each brain region, and the experiment repeated in at least 3 WT and KO littermate pairs. Example images show single z plane from the same location in the stack for both genotypes.

For immunostaining of PFA fixed tissue, following cryosectioning, the slides containing the sections were blocked for 1 hr at room temp in blocking buffer containing antibody buffer (100 mM L-lysine and 0.3% Triton X-100 in PBS) supplemented with 10% heat inactivated normal goat serum. Primary antibodies diluted in antibody buffer with 5% goat serum were incubated overnight at 4C. The next day slides were washed 3X 5 min with PBS with 0.2% Triton X-100 and secondary antibodies conjugated to Alexa fluor were applied for 2 hr at room temp. Slides were mounted with the SlowFade Gold with DAPI mounting media (Life Tech Cat. S36939), covered with 1.5 glass coverslip (Fisher Cat. 12544E) and sealed with clear nail polish. For experiments shown in Figures 7A, S6C, S7B, and S7D–S7F slides were imaged with fluorescent microscope (Zeiss Axio Imager.Z2) using the Apotome.2 module (Zeiss) at 10x, 20x or 40x magnifications. 16 bit images at 1388 X 1040 pixels were acquired using AxioCam HR3 camera (Zeiss). Z stack images (optical slice 1 μm; 5-7 μm stack) were obtained for each image. Set exposure was used when comparing WT and KO mice per given experiment. Example images show maximal intensity projection images.

Experiments testing RORα cre expression pattern in thalamo-cortical axons (Figures S7E and S7F, lower panels) were imaged using Zeiss LSM 780 confocal microscope at 63X magnification. For each section a 1420 X 1420 pixel 3 μm thick z stack image was obtained (optical slice 0.33 μm; 9 slices per 3 μm stack).

For experiments described in Figure 8 slides were imaged using Zeiss LSM 880 confocal microscope at 63X magnification. For each section a 1420 X 1420 pixel 3 μm thick z stack image was obtained (optical slice 0.33 μm; 9 slices per 3 μm stack). Set exposure was used when comparing WT and KO mice per given experiment. Images were analyzed using the Imaris software (Bitplane) in the same way as described above for fresh frozen NP1-VGlut2 colocalization. Example images show single z plane from the same location in the stack for both genotypes.

RNAscope In Situ *Hybridization*

RNAscope *in situ* hybridization experiments were performed according to manufacturer's instructions for fresh frozen tissue. The chromogenic assay shown in Figures 5A, 5B, 7J, and S4A was performed using the RNAscope 2.5 HD—BROWN Manual Assay kit (Acdbio Cat. 322300). Briefly, fresh frozen mouse brain tissue was sectioned at 16 μm, mounted on Superfrost plus slides (Fisher Cat. 1255015) and dried at −20C for 1 hr. Slides were stored at −80C until use. Slides were fixed for 15 min at 4C with 4% PFA, and then sequentially dehydrated with 50, 70 and 100% ethanol. Sections were then permeabilized with pretreatment reagent for 10-20 min (Protease plus Cat. 322330) and specific probe was applied to each slide. Slides were incubated with the probes for 2 hr at 40C in the hybridization oven (HybEZ hybridization system; ACDbio). After that, 6 amplification steps and 1 detection step were performed as per manufacturer instruction, and mRNA signal was detected with Dab reagent (ACDbio Cat. 322300). DAPI (1:1000; Millipore Cat 5.08741.0001) was applied to visualize the cell nuclei. Finally, slides were dehydrated again with sequential ethanol washes and cleared with xylene. Slides were mounted with Cytoseal mounting media (Thermo Cat. 8312-4) covered with 1.5 glass coverslip (Fisher Cat. 12544E) and left to dry overnight at room temp. Slides were imaged using the Axio Imager.Z2 fluorescent microscope (Zeiss). A 16 bit 1388 X 1040 pixel images were acquired using AxioCam HR3 camera (Zeiss). The Dab signal was imaged using the brightfield settings at 10x magnification; cell nuclei were imaged using the DAPI channel. Set exposure was used when comparing signal between WT and KO mice for a given experiment.

Fluorescent RNAscope In Situ *Hybridization*

The fluorescent *in situ* assay shown in Figures 5C, 5E, 6I, S4B, S4C, and S7G–S7I was performed using the RNAscope 2.5 HD—multiplex fluorescent Manual Assay kit (ACDbio Cat. 320850). In this assay tissue was prepared and pretreated in the same way as in the chromogenic assay (see above). After incubation with target probes, 3 amplification steps and 1 detection step were performed following manufacturer instructions. Slides were mounted using the SlowFade Gold with DAPI mounting media (Life Tech Cat. S36939) covered with 1.5 glass coverslip (Fisher Cat. 12544E) and sealed with clear nail polish. Slides were imaged using the Axio Imager.Z2 fluorescent microscope (Zeiss). A 16 bit 1388 X 1040 pixel images were acquired using AxioCam HR3 camera (Zeiss). Fluorescent signal was imaged at 20x magnification using the Apotome.2 module (Zeiss). Z stack images (optical slice 1 μm) were obtained for each image. Set exposure was used when comparing signal between WT and KO mice for a given experiment. For cell type specific expression quantification experiments shown in Figures 5C–5E slides were imaged using the Zeiss LSM 710 confocal microscope at 20X magnification. 16 bit images at 0.16 μm/pixel were generated. A z stack (optical slice 1 μm, 6 slices total) was obtained for each image. Example images show maximal intensity projections for each group.

Analysis and Quantification of Cell-Specific Expression of Gpc4 mRNA

For analysis shown in Figures 5C–5E 3 mice were used for each brain region, and for each mouse 3 brain sections were processed, imaged and analyzed. For analysis 2 regions of interest (ROI) of 130X130 μm were selected and cropped from each image. The images were thresholded to reveal positive signal in the same way for each image and the number of cells was then manually counted using the cell counter option of Fiji (ImageJ). The number of Gpc4 expressing cells was counted as well as the number of cells positive for both Gpc4 and Aldh1l1 (astrocyte specific expression) or Gpc4 and Tubb3 positive cells (neuronal specific expression). Finally a fraction of Gpc4+Aldh1l1 positive cells was plotted out of total Gpc4 expressing cells and compared to the fraction of Gpc4+Tubb3 positive cells.

QUANTIFICATION AND STATISTICAL ANALYSIS

All data is presented as mean ± s.e.m. Statistical analysis was performed using Sigma plot software (Systat software inc.). Multiple group comparisons were done using one-way Analysis of Variance (ANOVA) with post hoc Dunn's or Dunnet's tests. Pairwise comparisons were done by t test. When data did not pass normal distribution test, multiple comparisons were done by Kruskal-Wallis ANOVA on ranks and pairwise comparisons were done with Mann-Whitney Rank Sum test. P value $\leq$ 0.05 was considered statistically significant. The sample sizes, statistical tests used and significance are presented in each figure legend.

DATA AND SOFTWARE AVAILABILITY

The microarray data have been deposited in GEO: GSE86595.

Neuron, Volume *96*

Supplemental Information

Astrocyte-Secreted Glypican 4 Regulates Release of Neuronal Pentraxin 1 from Axons to Induce Functional Synapse Formation

Isabella Farhy-Tselnicker, Adriana C.M. van Casteren, Aletheia Lee, Veronica T. Chang, A. Radu Aricescu, and Nicola J. Allen

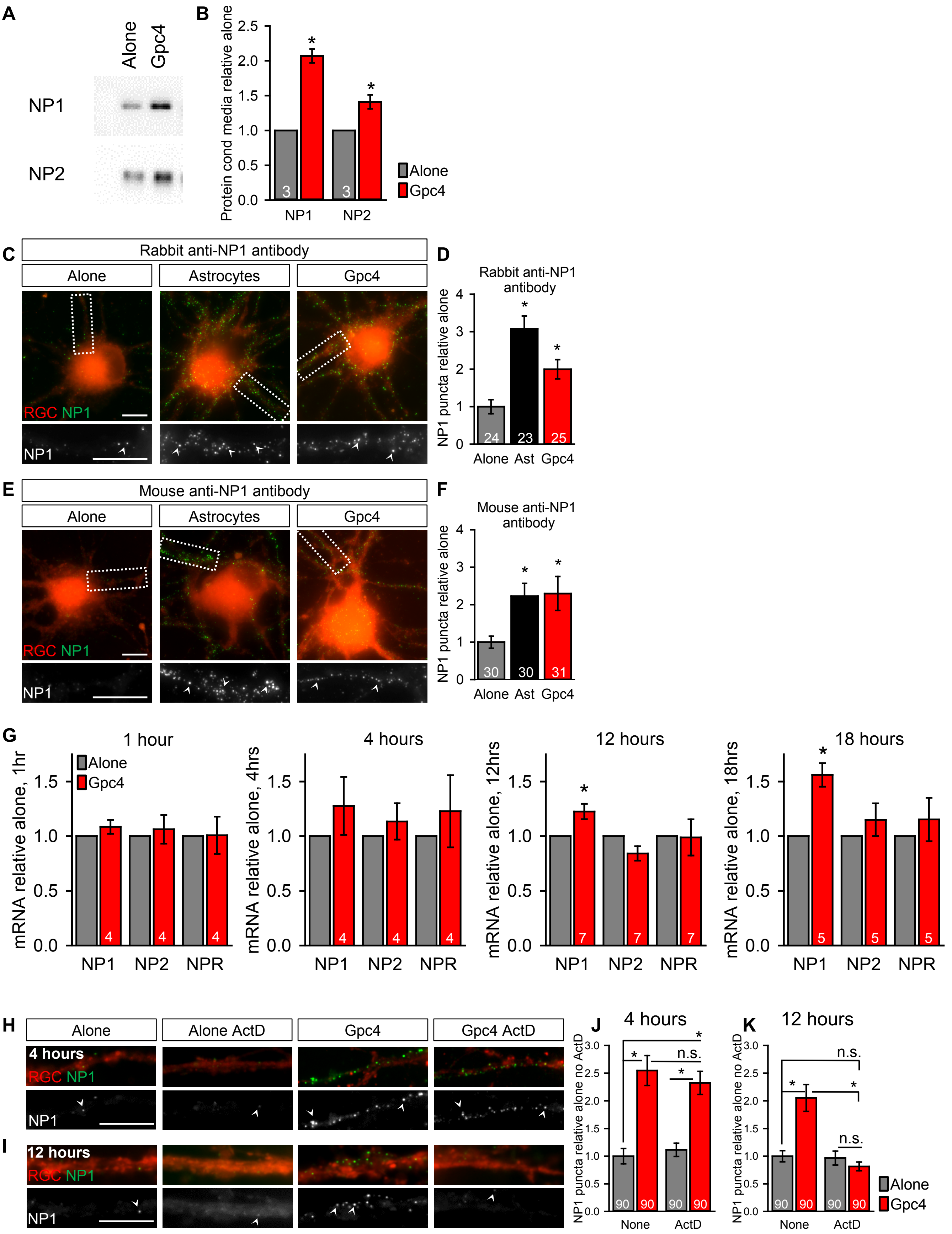
A
Alone
Gpc4
NP1
NP2
B
Protein cond media relative alone
NP1
NP2
Alone
Gpc4
C
Rabbit anti-NP1 antibody
Alone
Astrocytes
Gpc4
RGC NP1
NP1
D
Rabbit anti-NP1 antibody
NP1 puncta relative alone
Alone Ast Gpc4
E
Mouse anti-NP1 antibody
Alone
Astrocytes
Gpc4
RGC NP1
NP1
F
Mouse anti-NP1 antibody
NP1 puncta relative alone
Alone Ast Gpc4
G
1 hour
4 hours
12 hours
18 hours
mRNA relative alone, 1hr
mRNA relative alone, 4hrs
mRNA relative alone, 12hrs
mRNA relative alone, 18hrs
Alone
Gpc4
NP1 NP2 NPR
H
Alone
Alone ActD
Gpc4
Gpc4 ActD
4 hours
RGC NP1
NP1
I
12 hours
RGC NP1
NP1
J
4 hours
NP1 puncta relative alone no ActD
None ActD
n.s.
K
12 hours
NP1 puncta relative alone no ActD
None ActD
n.s.
Alone
Gpc4

Fig S1

Figure S1 (Relates to Figure 1). Gpc4 upregulates release of the AMPAR clustering factor NP1 from RGC neurons. A, B. RGCs treated with soluble Gpc4 for 4 days increase secretion of neuronal pentraxins into the conditioning media (CM). **A.** Western blot of RGC CM from neurons grown +/- Gpc4, blotted against NP1 or NP2. Approximate band size is 50 KDa. **B.** Quantification of Western blots in A, normalized to RGC alone. N=3 experiments. **C-F.** RGCs treated with an astrocyte feeder layer or soluble Gpc4 for 6 days show increased surface accumulation of NP1, demonstrated with two different anti-NP1 antibodies. The data in the main figure is with mouse anti-NP1 from BD Transduction Labs. **C,D** Rabbit anti-NP1. **E,F** Mouse anti-NP1. **C,E.** Example images: green surface NP1, red labels whole cell. Inset shows enlarged dendritic region from box, surface NP1 white. **D,F.** Quantification of C,E, number of surface NP1 puncta per RGC normalized to alone condition, from one representative experiment. For **C-F** Scale bar = 10µm. Arrowheads mark example puncta of NP1. Graphs show mean±s.e.m., number of cells per group inside the bar. **G.** qRT-PCR analysis of mRNA levels for NP1, NP2 and NPR in RGCs treated with soluble Gpc4 for varying times (1 hour, 4 hours, 12 hours, 18 hours). Graphs show mean±s.e.m., number of experiments inside the bar. **H-K.** RGCs treated with the transcription inhibitor Actinomycin D (ActD) show increased surface accumulation of NP1 in response to soluble Gpc4 after 4 hours (**H,J**) but not after 12 hours treatment (**I,K**). **H,I** Example dendrite images. Top: green surface NP1, red labels whole process. Bottom: single channel NP1, white. Scale bar = 10µm. Arrowheads mark example puncta of NP1. **J,K** Quantification of H and I respectively, number of surface NP1 puncta per RGC normalized to alone condition. N=3 experiments for each treatment time. Graphs show mean±s.e.m. number of cells per group inside the bar. * $p \leq 0.05$, by t-test when comparing 2 groups or one-way ANOVA when comparing 3 or more groups.

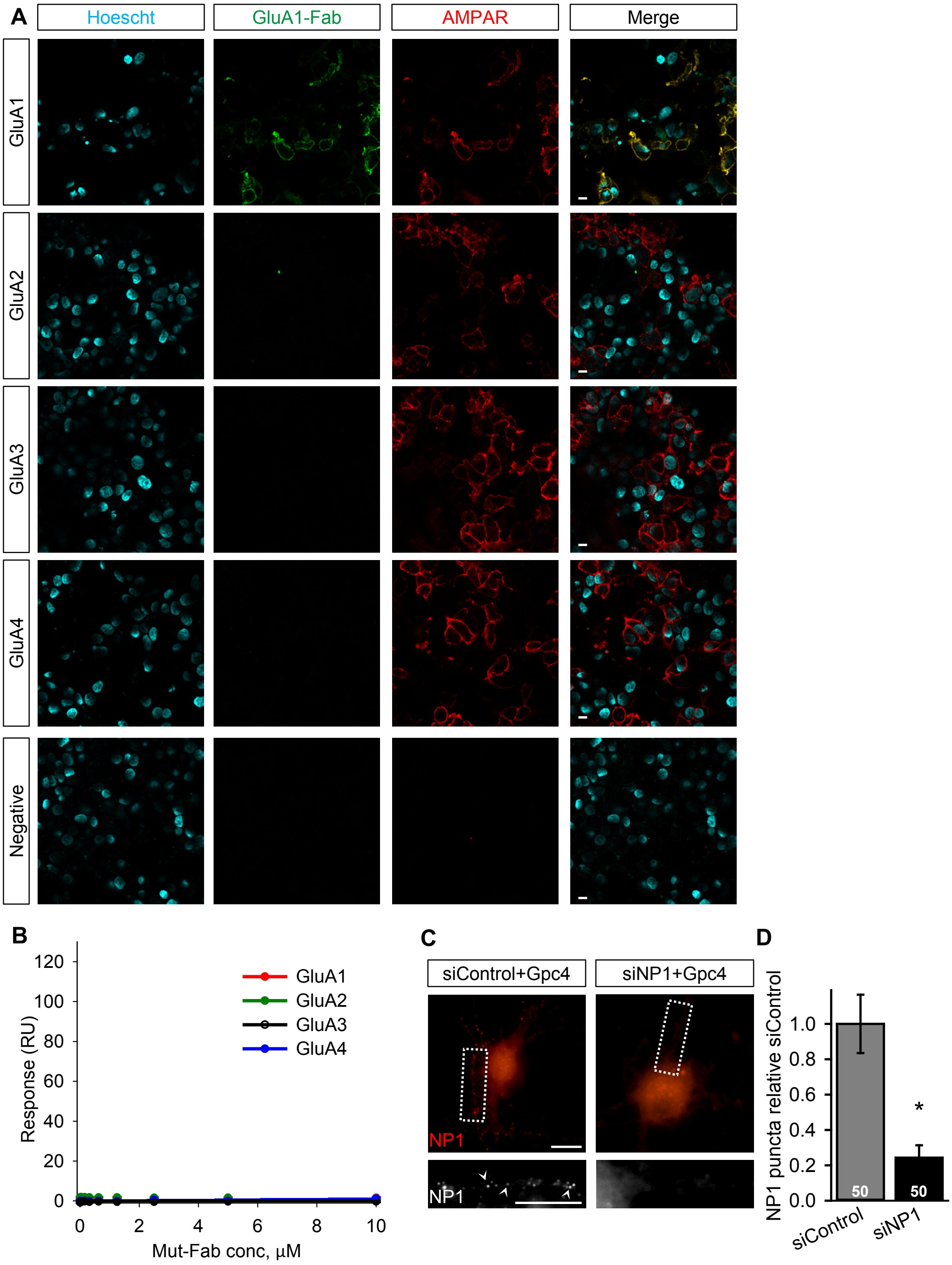
A
Hoescht
GluA1-Fab
AMPAR
Merge
GluA1
GluA2
GluA3
GluA4
Negative
B
Response (RU)
120
100
80
60
40
20
0
GluA1
GluA2
GluA3
GluA4
0
2
4
6
8
10
Mut-Fab conc, µM
C
siControl+Gpc4
siNP1+Gpc4
NP1
NP1
D
NP1 puncta relative siControl
1.0
0.8
0.6
0.4
0.2
0.0
*
50
50
siControl
siNP1

Fig S2

Figure S2 (Relates to Figure 2). **NP1-GluA1 interaction is necessary for Gpc4 to induce structural synapse formation. A,B.** The GluA1-Fab is specific for the N-terminal domain of GluA1 over other AMPAR subunits. **A.** Example images of HEK293T cells transfected with HA-tagged (GluA1, GluA3, GluA4) or FLAG-tagged (GluA2) AMPAR and co-immunostained for surface expression of the receptor and GluA1-Fab. Cell nuclei labeled with Hoescht (cyan) are shown on the left panels, followed by GluA1-Fab (green), AMPAR (red) and the merged image shown on the right. Note GluA1-Fab staining only visible in cells expressing the GluA1 subunit (top row). Negative control shows no signal (bottom row of images). Scale bar = 10μm. **B.** Surface plasmon resonance analysis of Mut-Fab binding. The Mut-Fab does not bind GluA1,2,3 or 4 AMPARs. **C-D.** siRNA against NP1 causes a significant decrease in surface NP1 accumulation in RGCs. **C**. Example images red: surface NP1. Scale bar = 10μm. Inset shows enlarged dendritic region from box, surface NP1 white. Scale bar = 10μm. Arrowheads mark example puncta of NP1. **D**. Quantification of C, number of surface NP1 puncta per RGC normalized to siControl+Gpc4 condition. N=2 experiments. Graphs show mean±s.e.m., number of cells per group inside the bar. * $p \leq 0.05$, by t-test.

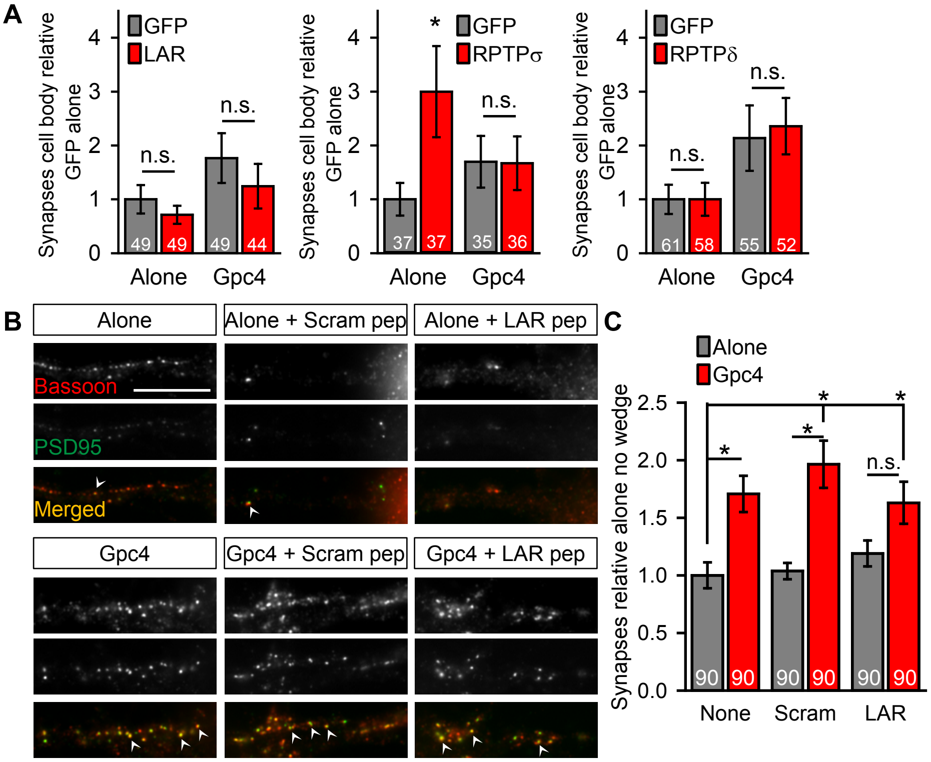
A
Synapses cell body relative GFP alone
GFP
LAR
RPTPσ
RPTPδ
n.s.
*
Alone
Gpc4
49
44
37
35
36
61
58
55
52
B
Alone
Alone + Scram pep
Alone + LAR pep
Bassoon
PSD95
Merged
Gpc4
Gpc4 + Scram pep
Gpc4 + LAR pep
C
Alone
Gpc4
Synapses relative alone no wedge
90
None
Scram
LAR

Fig S3

Figure S3 (Relates to Figure 3). Glypican 4 acts through presynaptic RPTPs to increase synapse formation. A. Expression of RPTP δ, σ or LAR in RGC dendrites does not alter the number of synapses formed in response to Gpc4. N = 3 experiments for each RPTP. **B-C.** Blocking LAR function with cell permeant wedge peptides does not alter Gpc4-mediated synapse formation. **B.** Example dendrite images, Bassoon red, PSD95 green, scale bar = 10μm. Arrowheads mark example synapses. **C.** Quantification of B, synapse number per cell normalized to alone no peptide condition, N = 3 experiments. Graphs show mean±s.e.m., number of cells per group inside the bar. * $p \leq 0.05$, by one-way ANOVA.

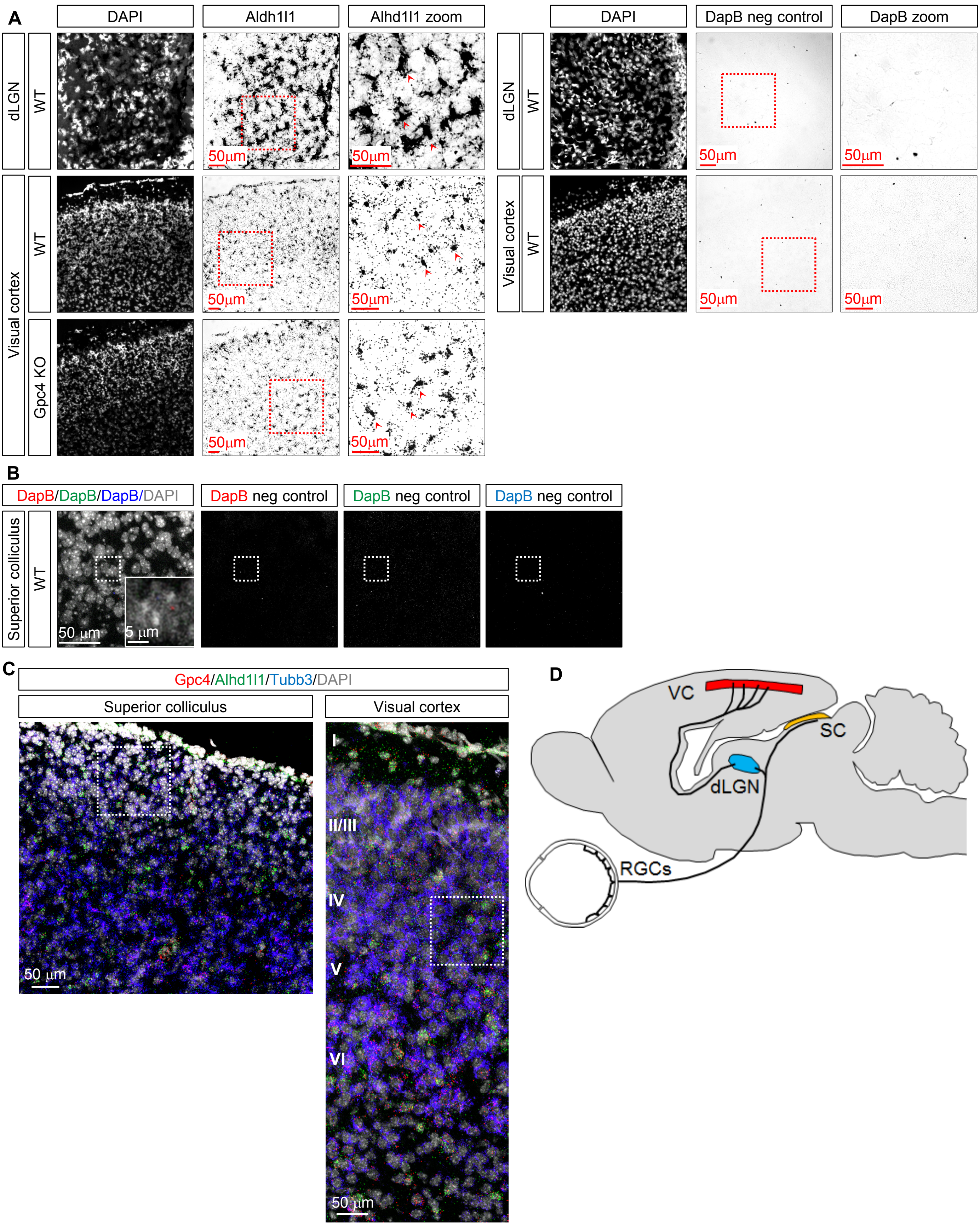
A
DAPI
Aldh1l1
Alhd1l1 zoom
DAPI
DapB neg control
DapB zoom
dLGN
WT
Visual cortex
WT
Gpc4 KO
50µm
B
DapB/DapB/DapB/DAPI
DapB neg control
DapB neg control
DapB neg control
Superior colliculus
WT
50 µm
5 µm
C
Gpc4/Alhd1l1/Tubb3/DAPI
Superior colliculus
Visual cortex
I
II/III
IV
V
VI
50 µm
D
VC
SC
dLGN
RGCs

Fig S4

Figure S4 (Relates to Figure 5). Expression of Gpc4 in the developing visual system. A. Left side, ISH in the developing visual system at P6 shows expression of mRNA for the astrocyte marker Aldh1l1, demonstrating astrocytes are present at the time when synapse formation is initiated – in the dLGN (top), and visual cortex of WT (middle) and Gpc4 KO (bottom). Right side, negative control probe, showing absence of signal in the dLGN (top) and visual cortex (bottom). In each set, left panels show DAPI to mark cell nuclei, middle panels are low power images of the mRNA probe, scale bar = 50μm, right panels are high power images of the region outlined by the red box in the low power image, red arrowheads mark positive cells, scale bar = 50μm. Note the different image size for VC and dLGN. **B.** Triple ISH using the negative control probes, showing absence of signal in each channel. DAPI (grey) marks cell nuclei. Inset shows zoom in of boxed region. Scale bar = 50μm, zoomed in box scale bar = 5μm. **C.** Overview of triple ISH images of superior colliculus (left) and visual cortex (right). DAPI (grey, nuclei), Gpc4 (red), Aldh1l1 (green, astrocytes), Tubb3 (blue, neurons). Box shows the area used for quantification described in Fig 5C-F. **D.** Diagram of the mouse visual system showing RGC axons project from the retina to the superior colliculus (SC) and dorsal lateral geniculate nucleus of the thalamus (dLGN). Visual cortex (VC) is innervated by axons projecting from dLGN neurons. Adapted from (Berson, 2003).

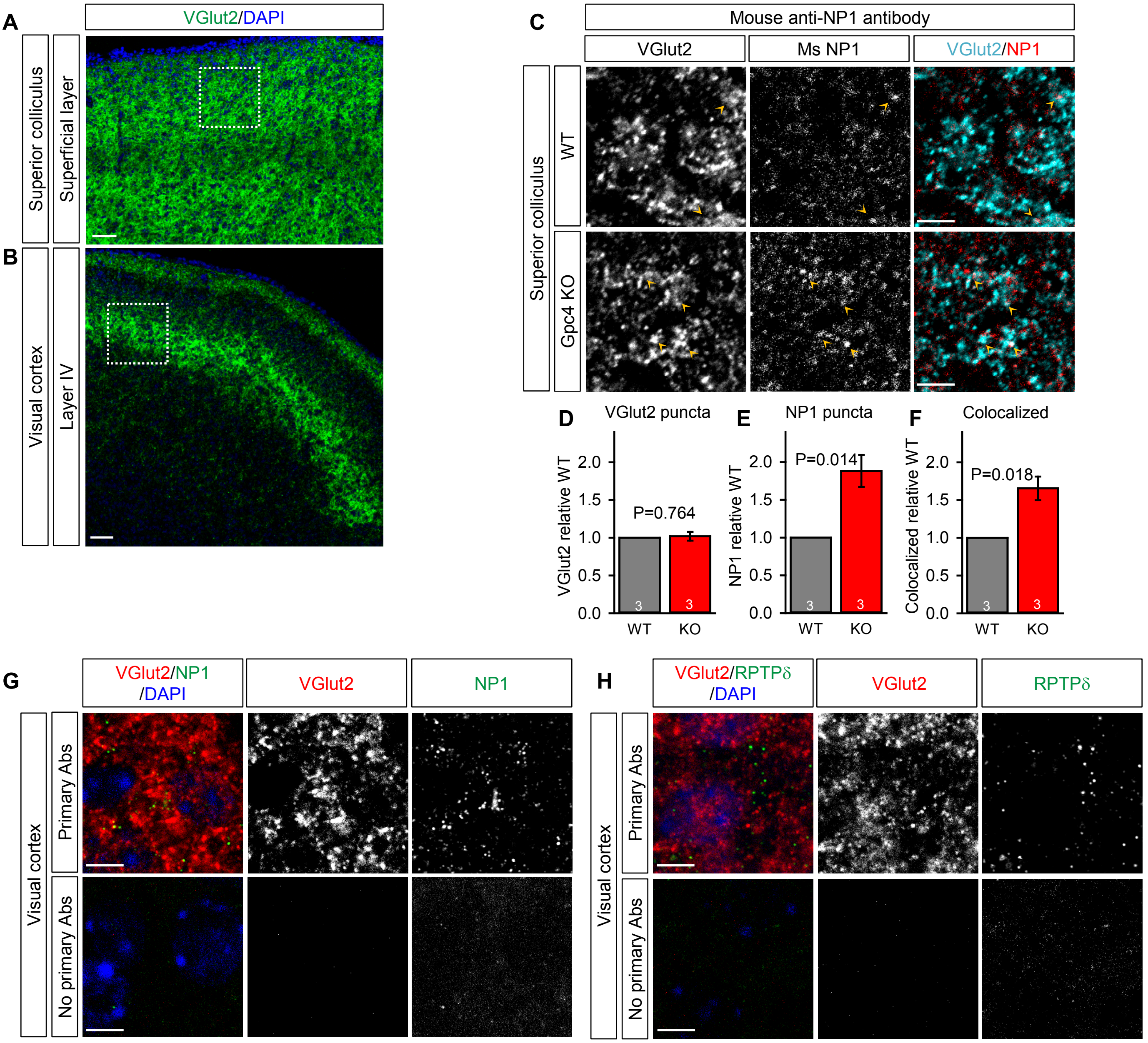
A
VGlut2/DAPI
Superior colliculus
Superficial layer
B
Visual cortex
Layer IV
C
Mouse anti-NP1 antibody
VGlut2
Ms NP1
VGlut2/NP1
Superior colliculus
WT
Gpc4 KO
D
VGlut2 puncta
VGlut2 relative WT
P=0.764
E
NP1 puncta
NP1 relative WT
P=0.014
F
Colocalized
Colocalized relative WT
P=0.018
0.0
0.5
1.0
1.5
2.0
3
WT
KO
G
VGlut2/NP1
/DAPI
VGlut2
NP1
Visual cortex
Primary Abs
No primary Abs
H
VGlut2/RPTPδ
/DAPI
VGlut2
RPTPδ
Visual cortex
Primary Abs
No primary Abs

Fig S5

Figure S5 (Relates to Figure 6). Gpc4 KO mice have increased overlap of NP1 with presynaptic terminals in the developing visual system *in vivo*. A-B. Low power confocal images of VGlut2 staining in superficial layers of the superior colliculus (A, SC) and layer 4 of the visual cortex (B, VC). White boxes mark example areas from which high power images in Fig 6-8 were taken. **C-F.** Increased colocalization between NP1 and the presynaptic marker VGlut2 in Gpc4 KO mice in the SC at P6, suggests reduced release of NP1 in the absence of Gpc4. Experiment carried out with mouse anti-NP1 from BD Transduction Labs. NP1 staining in the main figure is conducted with rabbit anti-NP1 from Thermo Fisher. The same result is seen with both anti-NP1 antibodies. **C.** Example images from the SC of WT (top) and Gpc4 KO (bottom), VGlut2 in cyan and NP1 in red. Representative colocalized puncta marked with arrowheads. Scale bar = 5 μm. **D,E,F.** Quantification of VGlut2, NP1 and colocalized puncta respectively, normalized to WT. Graphs show mean±s.e.m., number of mice inside the bar. Statistical analysis by t-test, P value shown on each graph. **G-H.** Representative images taken from the visual cortex with (top) and without (bottom) primary antibodies. VGlut2 red, NP1 green (G), RPTPδ green (H); DAPI in blue. Imaging the secondary antibody only condition with the same settings used for the primary antibody condition revealed no signal. Scale bar = 5 μm.

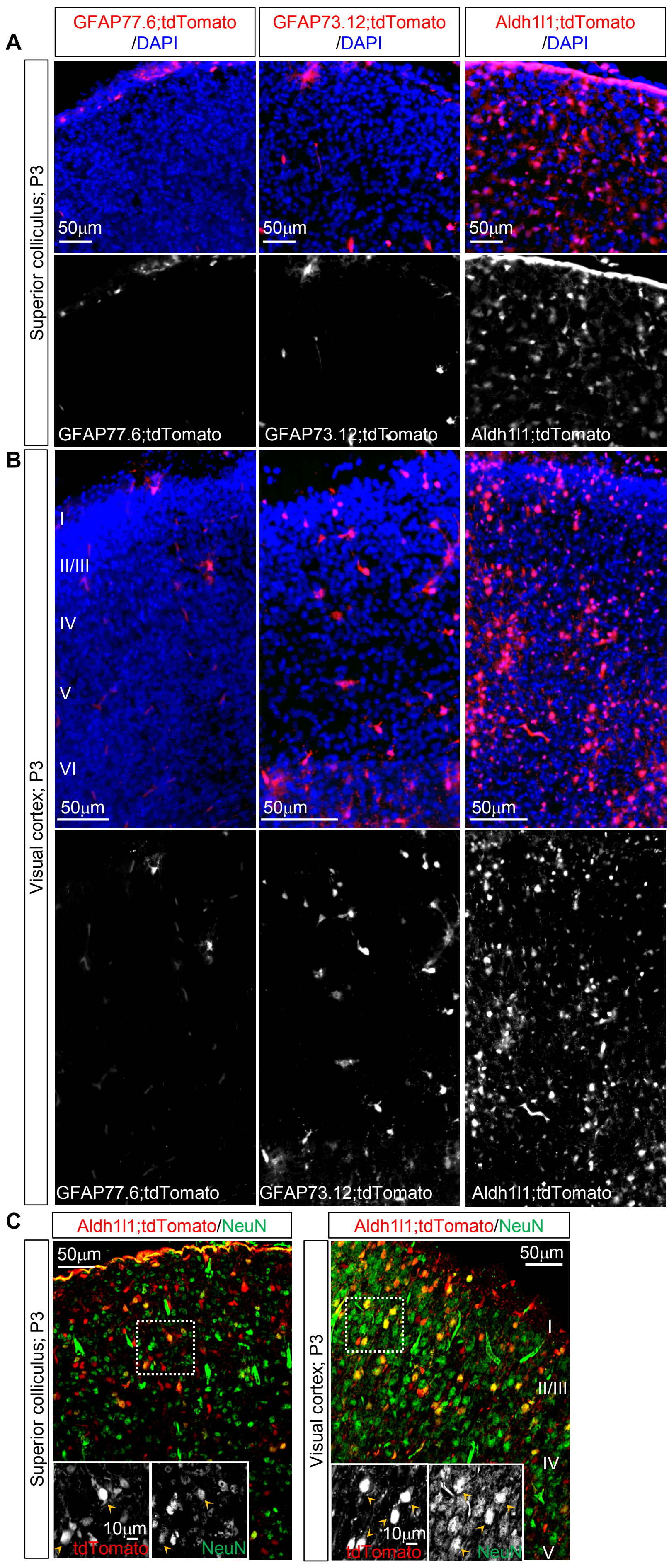
A
GFAP77.6;tdTomato/DAPI
GFAP73.12;tdTomato/DAPI
Aldh1l1;tdTomato/DAPI
Superior colliculus; P3
50μm
50μm
50μm
GFAP77.6;tdTomato
GFAP73.12;tdTomato
Aldh1l1;tdTomato
B
Visual cortex; P3
I
II/III
IV
V
VI
50μm
50μm
50μm
GFAP77.6;tdTomato
GFAP73.12;tdTomato
Aldh1l1;tdTomato
C
Aldh1l1;tdTomato/NeuN
Aldh1l1;tdTomato/NeuN
Superior colliculus; P3
50μm
10μm
tdTomato
NeuN
Visual cortex; P3
50μm
I
II/III
IV
V
10μm
tdTomato
NeuN

Fig. S6

Figure S6 (Relates to Figure 6). Characterization of astrocyte specific cre mouse lines. A-B. 3 different astrocyte specific cre lines GFAPcre77.6 (left); GFAPcre73.12 (middle) and Aldh1l1cre (right) were crossed to a tdTomato reporter line to visualize regions of cre expression in the SC (**A**) and VC (**B**) at P3. No substantial tdTomato expression was detected in GFAPcre77.6 in either brain region (left). No tdTomato expression was detected in GFAPcre73.12 SC (middle), whereas several tdTomato positive cells were detected in the VC. Robust tdTomato expression was detected in Aldh1l1cre line in both brain regions (right). DAPI (blue) marks cell nuclei, red marks tdTomato expressing cre positive cells. Below each merged image is a single channel tdTomato image. **C.** Immunostaining for the neuronal marker NeuN in Aldh1l1cre;tdTomato mouse brain, in the SC (left) and VC (right). Insets show zoom in box for single channel tdTomato (left) and NeuN (right). Scale bar = 50 μm, zoomed in box scale bar = 10 μm. Limited overlap of tdTomato with NeuN was observed in SC, while greater overlap was detected in VC. Arrowheads mark example overlapped cells.

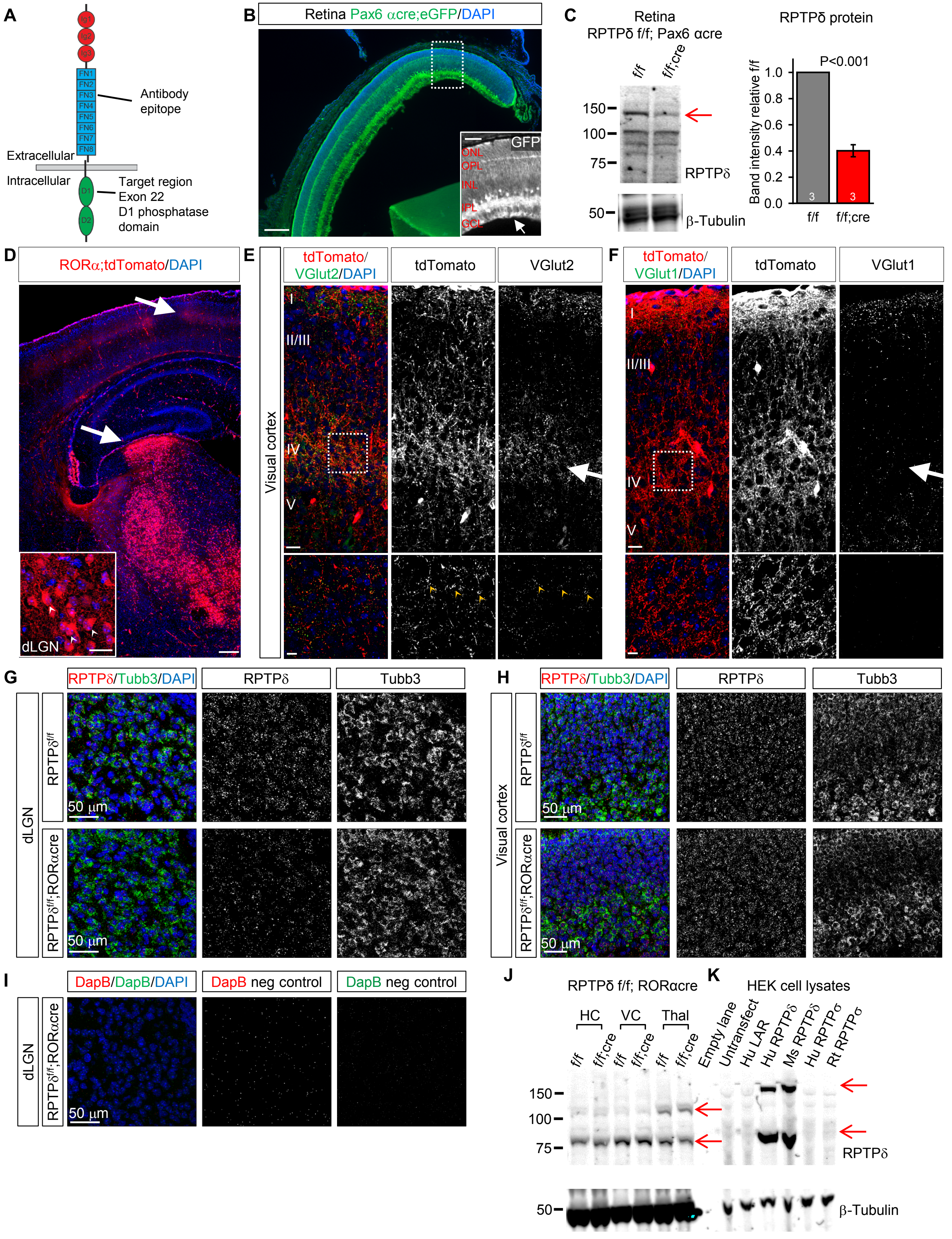

A
Ig1
Ig2
Ig3
FN1
FN2
FN3
FN4
FN5
FN6
FN7
FN8
Antibody epitope
Extracellular
Intracellular
D1
D2
Target region
Exon 22
D1 phosphatase domain
B
Retina Pax6 αcre;eGFP/DAPI
GFP
ONL
OPL
INL
IPL
GCL
C
Retina
RPTPδ f/f; Pax6 αcre
f/f
f/f;cre
150
100
75
50
RPTPδ
β-Tubulin
RPTPδ protein
P<0.001
Band intensity relative f/f
1.0
0.8
0.6
0.4
0.2
0.0
3
3
f/f
f/f;cre
D
RORα;tdTomato/DAPI
dLGN
E
Visual cortex
tdTomato/VGlut2/DAPI
tdTomato
VGlut2
I
II/III
IV
V
F
tdTomato/VGlut1/DAPI
tdTomato
VGlut1
I
II/III
IV
V
G
RPTPδ/Tubb3/DAPI
RPTPδ
Tubb3
dLGN
RPTPδf/f
RPTPδf/f;RORαcre
50 µm
50 µm
H
RPTPδ/Tubb3/DAPI
RPTPδ
Tubb3
Visual cortex
RPTPδf/f
RPTPδf/f;RORαcre
50 µm
50 µm
I
DapB/DapB/DAPI
DapB neg control
DapB neg control
dLGN
RPTPδf/f;RORαcre
50 µm
J
RPTPδ f/f; RORαcre
HC
VC
Thal
f/f
f/f;cre
f/f
f/f;cre
f/f
f/f;cre
150
100
75
50
K
HEK cell lysates
Empty lane
Untransfect
Hu LAR
Hu RPTPδ
Ms RPTPδ
Hu RPTPσ
Rt RPTPσ
RPTPδ
β-Tubulin

Fig. S7

Figure S7 (Relates to Figure 7). RPTPδ KO mice have increased overlap of NP1 with presynaptic terminals in the developing visual system *in vivo*. A. Diagram: structure of RPTPδ adapted from (Takahashi and Craig, 2013) showing the flox target region (D1 phosphatase domain), as well as the FN3 domain (target of the antibody). **B-C.** Validation of Pax6-a-cre line, demonstrating expression of cre recombinase in the developing retina. **B.** Pax6-a-cre was crossed to an eGFP reporter line to visualize regions of cre expression in the retina. GFP positive signal was observed in the ganglion cell layer (GCL), inner plexiform layer (IPL), a subset of cells in the inner nuclear layer (INL) and in the outer plexiform layer (OPL). Scale bar = 200 μm. GFP single channel inset shows a zoomed in image of the box. Arrow points to the ganglion cell layer neurons positive for GFP expression. Scale bar = 50 μm. **C.** Decrease in the amount of RPTPδ protein in retina lysates from RPTPδ fl/fl Pax6-α-cre +ve mice detected by western blot using the specific anti-RPTPδ antibody. Only the C isoform (~150 KDa bands) is expressed in the retina (Shishikura et al., 2016). 20μg retinal protein lysate was loaded in each lane. An example blot is shown on the left and quantification of band intensities is on the right. β-Tubulin antibody was used as loading control (bottom panel) shown in a separate blot. Graph shows mean±s.e.m., number of mice per genotype inside the bar. Statistical analysis by t-test, P value shown on the graph. **D-F.** Validation of RORα cre line, demonstrating expression of cre recombinase in the developing thalamus including thalamocortical projection neurons. RORα cre was crossed to a tdTomato reporter line to visualize regions of cre expression. **D.** Low power image of RORα cre +ve; tdTomato +ve forebrain at P6, tdTomato red and DAPI to mark nuclei in blue. Arrows mark tdTomato expression in the dLGN and thalamocortical axon projections in the visual cortex. Scale bar = 200μm. Inset shows zoomed in region of dLGN neurons expressing tdTomato. Scale bar = 20μm. **E-F.** Colabeling of thalamocortical projections in RORα cre mouse marked with tdTomato expression, along with presynaptic excitatory markers VGlut2 and VGlut1. Low power image shows all cortical layers. Scale bar = 20μm, inset shows boxed region in layer 4. Scale bar = 10μm. Arrows show thalamoroctical projections to layer 4. **E.** Extensive overlap of tdTomato expressing axon terminals with the thalamocortical presynaptic marker VGlut2. TdTomato in red, VGlut2 in green, DAPI in blue. Right panels show single channel images as labeled. Arrowheads mark example colocalized puncta. **F.** No overlap of tdTomato expressing axon terminals with the intracortical presynaptic marker VGlut1. TdTomato in red, VGlut1 in green, DAPI in blue. Right panels show single channel images as labeled. **G-I** RPTPδ mRNA is expressed in the developing visual system, and the mRNA levels are reduced in the thalamus but not in the cortex when crossing RPTPδ fl/fl mice to RORα cre line. Double ISH for RPTPδ mRNA along with the neuronal marker Tubb3 in the developing visual system at P6. **G.** RPTPδ expression in RPTPδ fl/fl cre –ve (top) and RPTPδ fl/fl RORα cre +ve (bottom) in dLGN. **H.** RPTPδ expression in RPTPδ fl/fl cre –ve (top) and RPTPδ fl/fl RORα cre +ve (bottom) in the VC. RPTPδ in red; Tubb3 in green, and DAPI to mark cell nuclei in blue. Scale bar = 50μm. **I.** No signal was detected using the negative control probe. **J.** Decrease of the amount of RPTPδ protein in thalamus lysates of RPTPδ fl/fl RORα cre +ve mice detected by western blot using the specific anti-RPTPδ antibody (compare lanes marked Thal ~80KDa bands; D isoform). No change in the amount of RPTPδ in hippocampal or cortical lysates (compare HC and VC lanes ~80KDa bands). C and D isoforms are detected in the thalamus, only D isoforms are observed in HC and VC. Note decrease only in D isoform level in the thalamus. **K.** To confirm the specificity of the anti-RPTPδ antibody for RPTPδ over σ and LAR, HEK cells were transfected with different RPTPs and lysates probed by western blot. 10μg lysate was loaded in each lane, β-Tubulin antibody was used as loading control (bottom panel) shown in a separate blot. Anti RPTPδ antibody successfully detected mouse (Ms) and human (Hu) RPTPδ in HEK cell lysate and did not detect the other family members LAR or RPTPs, human or rat (Rt). Samples shown in **J** and **K** were probed on the same blot.

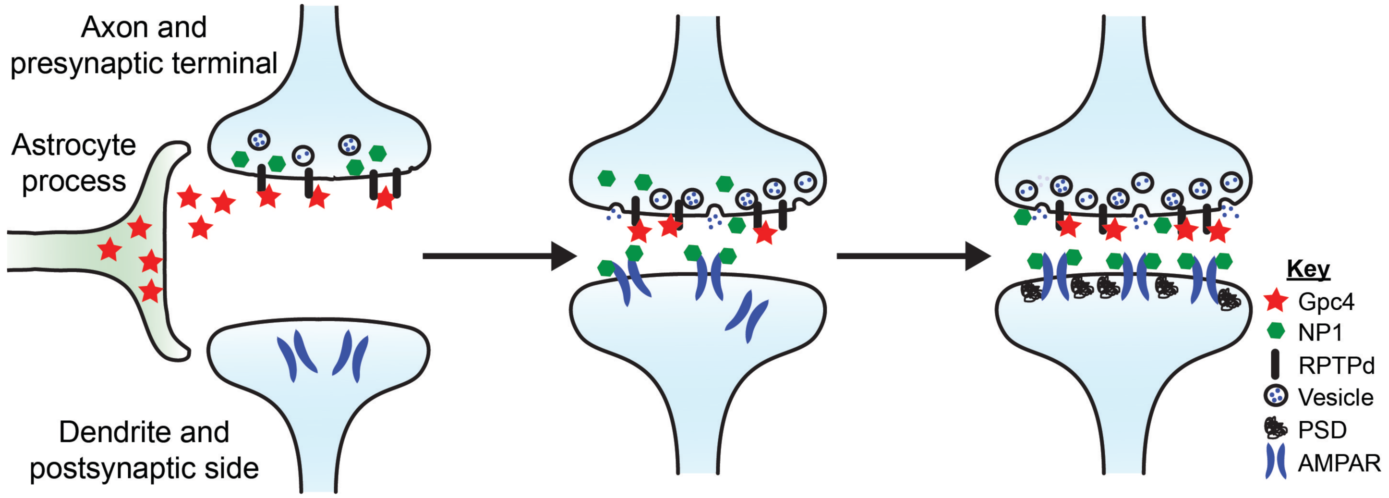
Axon and presynaptic terminal
Astrocyte process
Dendrite and postsynaptic side
Key
Gpc4
NP1
RPTPd
Vesicle
PSD
AMPAR

Fig. S8

Figure S8. Model of Gpc4 mechanism of action. Astrocyte-secreted glypican 4 (Gpc4) binds to RPTPδ on axons, leading to release of the AMPA receptor clustering factor neuronal pentraxin 1 (NP1) from presynaptic terminals. NP1 binds to the extracellular region of GluA1 AMPARs on nearby dendrites, stabilizing them on the dendritic surface and inducing nascent synapse formation.

Gene Symbol	Description	Alone (log2)	Gpc4 (log2)	TSP1 (log2)	Gpc4 vs Alone Fold	Gpc4 vs Alone p	TSP1 vs Alone Fold	TSP1 vs Alone p
Gria1	Glutamate receptor, ionotropic, AMPA 1	7.24	7.26	7.20	1.01	0.85	-1.03	0.75
Gria2	Glutamate receptor, ionotropic, AMPA 2	10.63	10.38	10.54	-1.19	0.14	-1.06	0.12
Gria3	Glutamate receptor, ionotropic, AMPA 3	6.77	6.65	6.72	-1.09	0.36	-1.04	0.75
Gria4	Glutamate receptor, ionotropic, AMPA 4	8.95	8.97	9.05	1.01	0.92	1.07	0.62
Cacng2	Stargazin	9.06	8.72	8.83	-1.26	0.25	-1.17	0.16
**Cacng3**	TARP gamma 3	7.52	6.99	7.21	***-1.44***	***0.047***	-1.25	0.20
Cacng4	TARP gamma 4	5.61	5.59	5.50	-1.02	0.90	-1.08	0.65
**Cacng5**	TARP gamma 5	4.37	4.63	4.50	***1.20***	***0.041***	1.10	0.40
Cacng7	TARP gamma 7	5.49	5.64	5.39	1.11	0.08	-1.07	0.22
Cacng8	TARP gamma 8	4.51	4.40	4.33	-1.08	0.66	-1.13	0.47
Cdh2	N cadherin	10.53	10.44	10.59	-1.06	0.26	1.04	0.46
Cnih	Cornichon	11.03	11.10	11.05	1.05	0.45	1.01	0.52
Cnih2	Cornichon 2	9.98	9.95	9.91	-1.02	0.40	-1.05	0.54
Cnih4	Cornichon 4	8.33	8.36	8.47	1.02	0.78	1.10	0.23
Dlg1	SAP97	6.56	6.64	6.46	1.06	0.31	-1.07	0.38
Dlg3	SAP102	5.02	5.05	5.22	1.02	0.80	1.15	0.35
Dlg4	PSD95	9.15	9.14	9.10	-1.01	0.21	-1.04	0.49
Epb41l1	4.1N	9.89	9.84	9.85	-1.03	0.67	-1.02	0.72
Grip1	Glutamate receptor interacting protein 1	9.19	9.12	9.32	-1.05	0.07	1.09	0.43
**Grip2**	Glutamate receptor interacting protein 2	6.15	6.18	6.18	1.02	0.81	***1.03***	***0.040***
**Nptx1**	Neuronal pentraxin I	11.31	12.18	11.23	***1.83***	***0.00029***	-1.06	0.69
Nptxr	Neuronal pentraxin receptor	10.72	10.74	10.76	1.02	0.11	1.03	0.30
Nsf	N-ethylmaleimide-sensitive factor	11.67	11.67	11.60	-1.00	0.98	-1.05	0.42
Pick1	Protein interacting with PRKCA 1	9.02	8.98	8.95	-1.03	0.53	-1.05	0.33
**Syndig1**	Synapse differentiation inducing 1	6.81	7.65	6.74	***1.79***	***0.010***	-1.06	0.53
**Tspan7**	Tetraspanin 7	12.17	12.20	12.20	1.02	0.10	**1.02**	**0.05**

Table S2 (Relates to Figure 1). Expression level of genes related to AMPAR trafficking and clustering in RGC neurons that are untreated or treated with Gpc4 or TSP1 for 12 hours, taken from full Affymetrix microarray data set in Table S1. Candidate genes significantly altered ($P \leq 0.05$) by Gpc4 or TSP1 treatment are shown in bold.

Receptor	Probe Set ID	Alone	Gpc4	TSP1
RPTPδ	1374591_at	564	522	590
LAR	1368036_at	1194	1088	1126
RPTPσ	1387901_at	3236	2945	2880

Table S3 (Relates to Figure 3). Expression level of type 2a RPTP family members in rat RGC neurons *in vitro*, taken from full Affymetrix microarray data set in Table S1.
